# Stepwise 6H^+^/6e^–^ Electron-Coupled Proton Buffers Based on Fe and Redox-Active Ligands

**DOI:** 10.1021/acs.inorgchem.5c02720

**Published:** 2025-09-22

**Authors:** Rajdeep Sarma, Tong Wu, Daniel Ye, Yi Lin Qiu, Serim Park, Emma Cohen, Jin Xiong, Maxime A. Siegler, Yisong Guo, Isaac Garcia-Bosch

**Affiliations:** † Department of Chemistry, 6612 Carnegie Mellon University, Pittsburgh, Pennsylvania 15213, United States; ‡ 1466 Johns Hopkins University, Baltimore, Maryland 21218, United States

## Abstract

Herein, we report electron-coupled-proton buffers (ECPBs) based on Fe and redox-active *ortho*-phenylenediamine (opda) ligands that perform stepwise and reversible 6H^+^/6e^–^ transformations. Four of the Fe complexes involved in the PCET transformation (namely ^**X**^ **6** ^**2+**^, ^**X**^ **8H**_**2**_^**2+**^, ^**X**^ **10H**_**4**_^**2+**^ and ^**X**^ **12H**_**6**_^**2+**^) were structurally and/or spectroscopically characterized. The reductive protonation of ^**X**^ **6** ^**2+**^ to ^**X**^ **12H**_**6**_^**2+**^ and the oxidative deprotonation of ^**X**^ **12H**_**6**_^**2+**^ to ^**X**^ **6** ^**2+**^ were carried out using PCET reagents, which indicate that these 6H^+^/6e^–^ transformations occurred in a 2H^+^/2e^–^ fashion, accumulating the intermediate species ^**X**^ **8H**_**2**_^**2+**^and ^**X**^ **10H**_**4**_^**2+**^. The thermochemistry of the 2H^+^/2e^–^ and overall 6H^+^/6e^–^ transformations was studied by open-circuit potential measurements and comproportionation reactions. Interestingly, the Fe-based ECPBs depicted redox unleveling, in which the average bond dissociation free energy (BDFE_avg_) of the 2H^+^/2e^–^ reductive protonation of ^**X**^ **6** ^**2+**^ to ^**X**^ **8H**_**2**_^**2+**^ was substantially higher than the BDFE_avg_ of the 6H^+^/6e^–^ conversion of ^**X**^ **6** ^**2+**^ to ^**X**^ **12H**_**6**_^**2+**^. We also show that the BDFE_avg_ of the PCET transformations involving the Fe system bearing unsubstituted opda are higher than the systems bound by 4,5-Me_2_-opda and 4,5-(MeO)_2_-opda, a manifestation of redox decompensation. The capability of the Fe-based ECPBs to accept and donate H-atom equivalents, as well as their ability to dehydrogenate organic substrates using O_2_ as oxidant in a decoupled fashion, was also evaluated.

## Graphical abstract

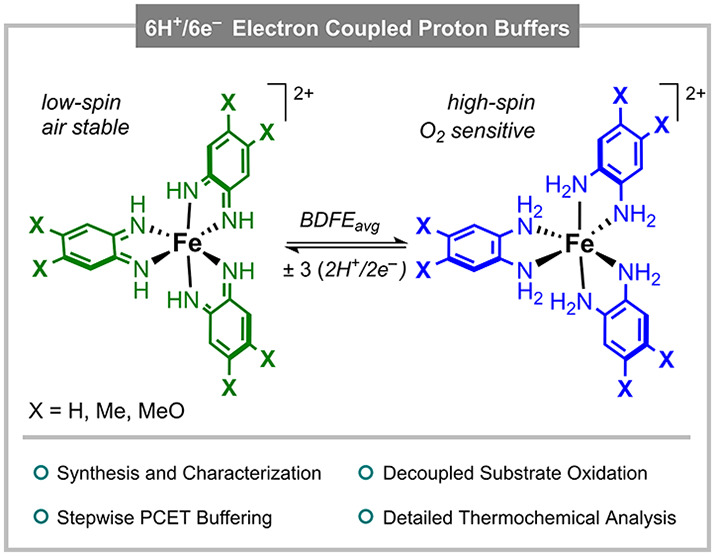

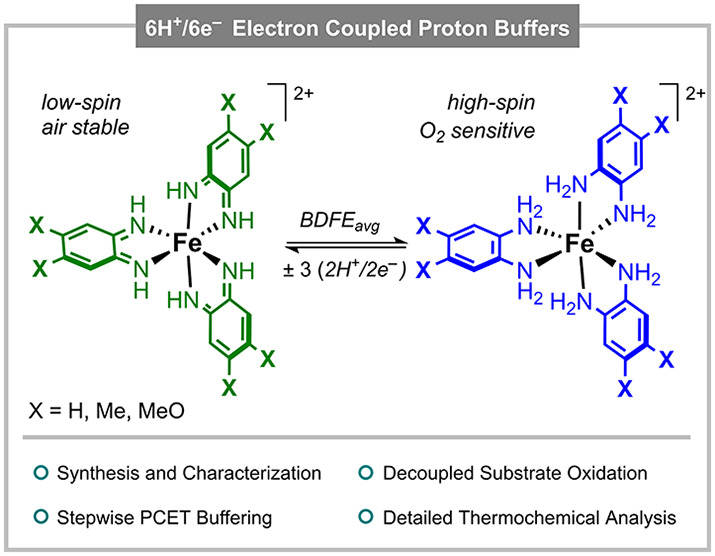

Proton-coupled electron transfer (PCET^1–4^ reactions are involved in a myriad of natural and industrial processes including the 4H^+^/4e^–^ reduction of O_2_ to H_2_O (and the reverse 4H^+^/4e^–^ oxidation of O_2_ to H_2_O),^5–7^ CO_2_ and H^+^ reduction,^8,9^ the functionalization of organic substrates,^10–13^ and redox processes.^14^ Electron-coupled proton buffers (ECPBs, a term coined by Symes and Cronin^15^ are organic and inorganic redox mediators capable of accepting, storing, and delivering protons and electrons in a reversible fashion. ECPBs are used by nature to handle the proton(s) and electrons(s) involved in water oxidation and cellular respiration. For example, the four protons and four electrons derived from water oxidation in photosystem II are transported to photosystem I by a pool of plastoquinone molecules, allowing to separate in time and space the generation of O_2_ with the biosynthesis of NADPH and ATP (Figure 1A).^16^ Artificial ECPBs have been recently used in electrochemical devices that allow to perform water electrolysis in a decoupled fashion (Figure 1B).^15,17^ The first ECPB developed for this purpose was based on a polyoxometalate based on Mo, but recent ECPBs based on the hydroquinone/quinone couple have also been reported. These ECPBs introduce an additional electrochemical step, which allows to separate, in time and space, the O_2_ evolving reaction (H_2_O is deprotonated/oxidized to O_2_ by the Pt electrode, and the resulting protons and electrons are used to reduce the ECPB) and the H_2_ evolving reaction (the protons and electrons stored in the ECPB are used to produce H_2_ by the Pt electrode).^18^ The main advantages of this technology is that the electrochemical cell operates at relatively low operational potentials (determined by the potential difference of the ECPBs and the Pt HER and OER), the gases synthesized are of high purity, and the increased durability of the cell components.

**Figure 1:**
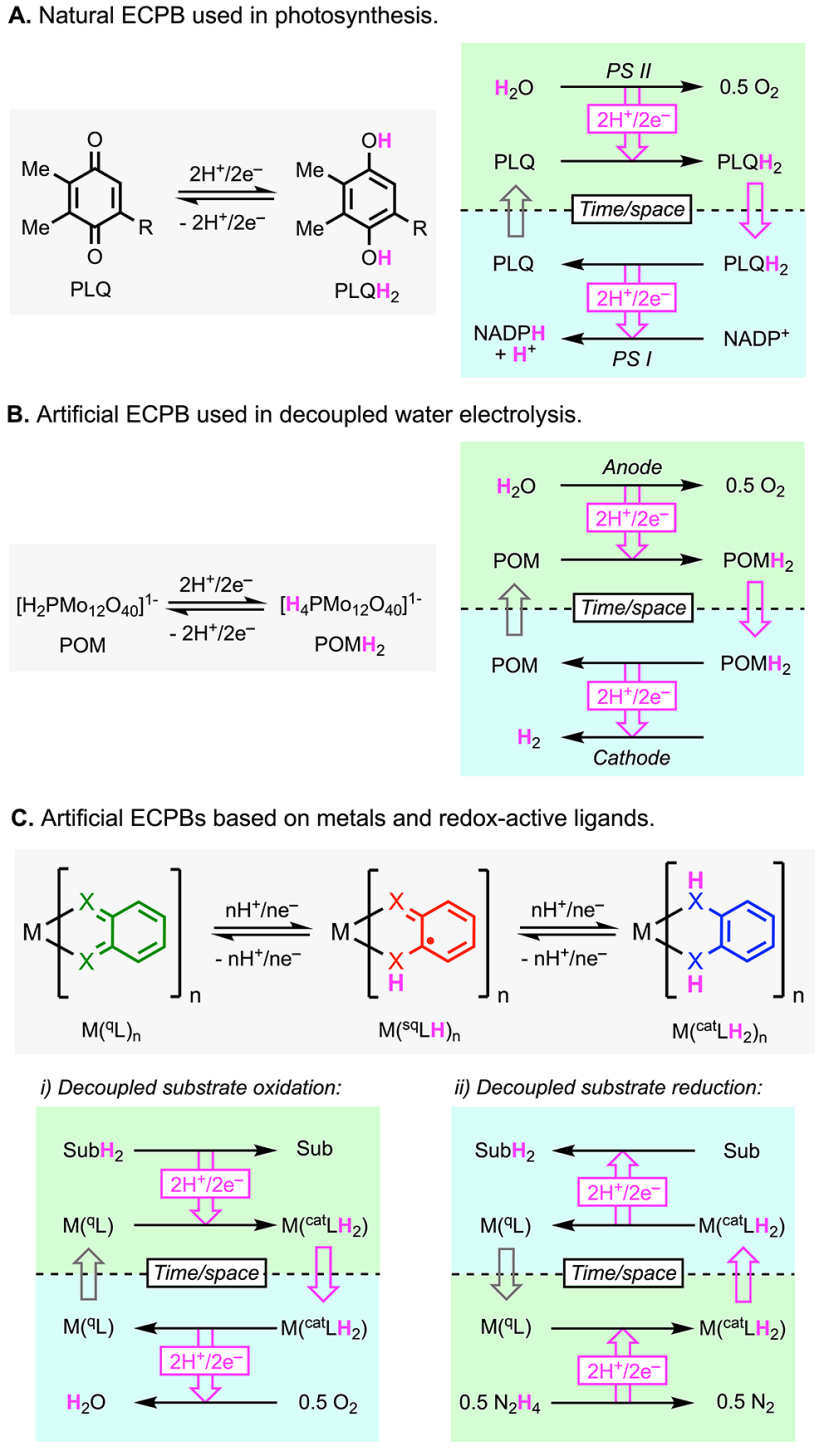
Natural (A) and artificial (B) electron-coupled proton buffers (ECPBs) that have inspired the development of ECPBs based on metals and redox-active ligands (C).

Our research lab is interested in developing ECPBs based on transition metals (e.g., Cu, Fe) and redox-active ligands to use them in PCET transformations (Figure 1C). Our systems do not behave like traditional ECPBs because in addition to accepting, storing, and delivering H atom equivalents, they can also activate small molecules. For example, our ECPBs can be used to separate, in time and space, the reductive protonation of O_2_ to H_2_O with the dehydrogenation of organic substrates (decoupled substrate oxidation). One of the advantages of this approach is that after reacting the ECPB with O_2_, the dehydrogenation of the substrate can be performed under anaerobic conditions, avoiding the direct interaction between O_2_ and the organic substrate that can lead to unwanted side reactions (e.g., dehydrogenation vs oxygenation of the substrate). Analogously, ECPBs can potentially be utilized in the decoupled hydrogenation of organic substrates in which the reductive protonation of the ECPB with H atom equivalents (e.g., hydrazine or H_2_) is separated in time and space with the EPCB-promoted hydrogenation of the organic substrate.

We have recently reported ECPBs based on Cu and redox-active ligands that promote reversible 4H^+^/4e^–^ transformations (Figure 2A).^19,20^ The ^**X,R**^ **5** ^**+**^/^**X,R**^ **1H**_**4**_^**+**^ systems reacted with H^+^/e^–^ donors and acceptors in a stoichiometric fashion to convert ^**X,R**^ **5** ^**+**^ to ^**X,R**^ **1H**_**4**_^**+**^, and ^**X,R**^ **1H**_**4**_^**+**^ to ^**X,R**^ **5** ^**+**^. The ability to buffer protons and electrons was demonstrated by reacting ^**X,R**^ **5** ^**+**^/^**X,R**^ **1H**_**4**_^**+**^ mixtures with substoichiometric amounts of PCET reagents, shifting the ^**X,R**^ **5** ^**+**^/^**X,R**^ **1H**_**4**_^**+**^ equilibria. The ^**X,R**^ **5** ^**+**^/^**X,R**^ **1H**_**4**_^**+**^ systems were also used in the decoupled dehydrogenation of organic substrates (e.g., hydroquinones to quinones) using O_2_ as terminal oxidant, a stoichiometric process that could be repeated for several cycles with minor mass balance loss. The thermochemistry of PCET processes involved in the 4H^+^/4e^–^ transformation between ^**X,R**^ **5** ^**+**^ and ^**X,R**^ **1H**_**4**_^**+**^ was also studied. Open-circuit potential measurements (a technique recently developed by Mayer and coworkers^21^ allowed for determining the average bond dissociation free energy (BDFE_avg_) of the 4 N–H bonds formed/broken in the interconversion between ^**X,R**^ **5** ^**+**^ and ^**X,R**^ **1H**_**4**_^**+**^. Interestingly, the BDFE_avg_ could be tuned by changing the substituents of the opda ligand, which led to small changes in the energetics associated with proton transfer (p*K*a) when compared to the energetics of electron transfer (redox potentials, *E*_1/2_), leading to thermochemical decompensation. We were also able to estimate the thermochemistry of the 1H^+^/1e^–^ reductive-protonation of ^**X,R**^ **5** ^**+**^ (BDFE_1_) and the 1H^+^/1e^–^ oxidative-deprotonation of ^**X,R**^ **1H**_**4**_^**+**^ (BDFE_4_), which differed significantly from the BDFE_avg_, an indication of redox unleveling.

**Figure 2:**
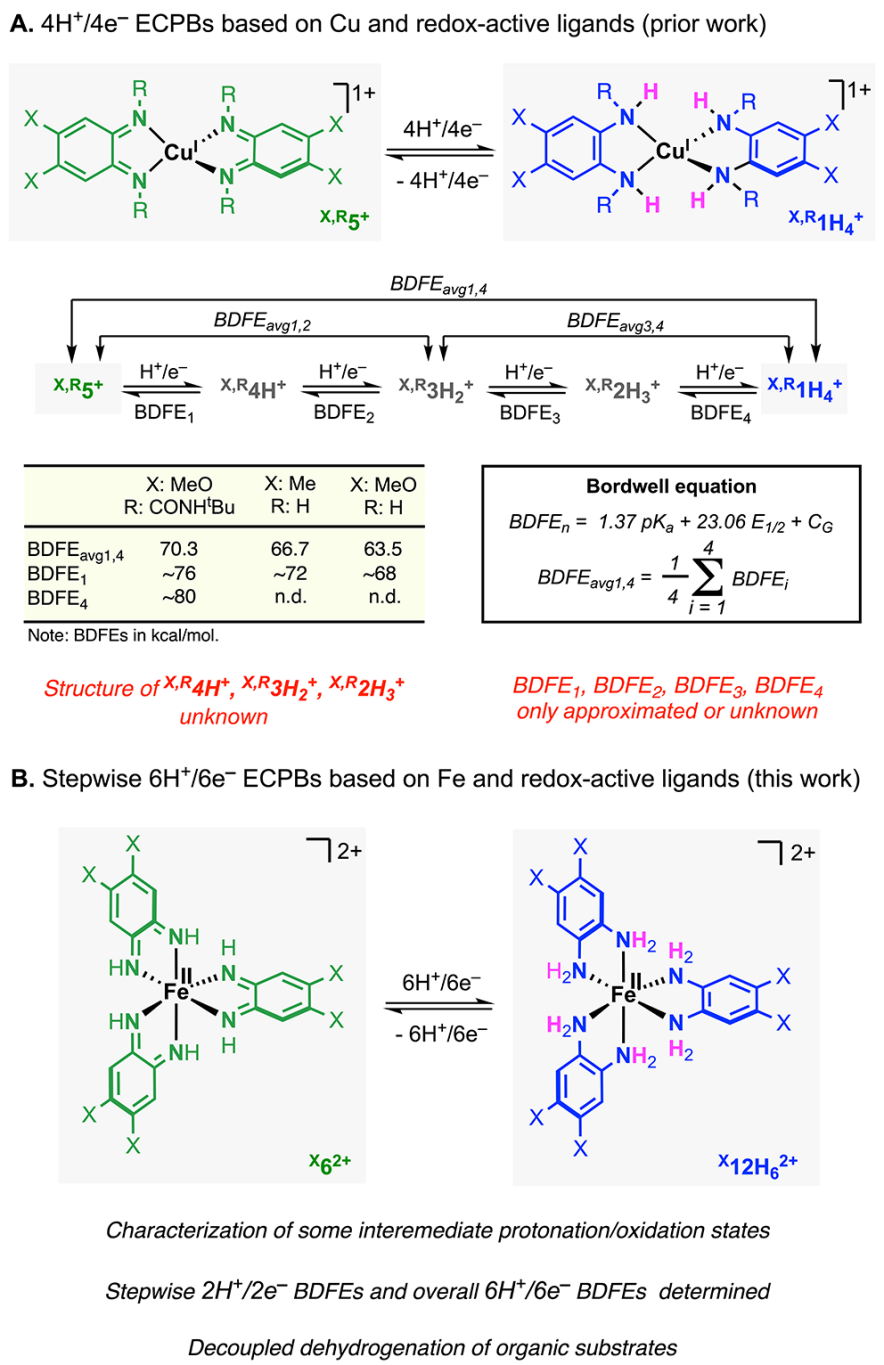
Cu-based ECPBs previously developed by our lab (A) and Fe-based ECPBs reported in this article (B).

Mechanistic studies suggested that the PCET reactivity of the Cu-based ECPBs involved fast disproportionation and ligand-exchange reactions, leading to unusual equilibria between ^**X,R**^ **5** ^**+**^/^**X,R**^ **1H**_**4**_^**+**^, complexes separated by 4 protons and 4 electrons. This behavior precluded the isolation and characterization of the three protonation/oxidation states between ^**X,R**^ **5** ^**+**^/^**X,R**^ **1H**_**4**_^**+**^ (namely ^**X,R**^ **4H** ^**+**^, ^**X,R**^ **3H**_**2**_^**+ X,R**^ **2H**_**3**_^**+**^), which also impeded a complete thermochemical analysis of the overall and stepwise PCET processes. Herein, we describe that the utilization of Fe^II^, instead of Cu^I^, not only allows to develop the first family of 6H^+^/6e^–^ ECPBs but also to stabilize some of the intermediate protonation/oxidation states, which permits to study the thermochemistry of the stepwise 2H^+^/2e^–^ and overall 6H^+^/6e^–^ PCET transformations involved in the ^**X,R**^ **6** ^**+**^/^**X,R**^ **12H**_**6**_^**+**^ interconversion (Figure 2B).

## Results and Discussion

### Synthesis and SC-XRD Characterization of the Fe Complexes

In 2013, Matsumoto, Chang and coworkers reported the synthesis and characterization of the iron complexes ^**H**^ **12H**_**6**_^**2+**^ and ^**H**^ **6** ^**2+**^ using perchlorate as counteranion.^22^ The authors prepared complex ^**H**^ **12H**_**6**_^**2+**^ by reacting 3 equiv of opda with Fe^II^(ClO_4_)_2_·6H_2_O in THF. Complex ^**H**^ **6** ^**2+**^ was prepared via oxidation of ^**H**^ **12H**_**6**_^**2+**^ with O_2_. In 2020, the same research group prepared ^**H**^ **6** ^**2+**^, ^**H**^ **8H**_**2**_^**2+**^, ^**H**^ **10H**_**4**_^**2+**^ using hexafluorophosphate as counteranion.^23^ Complex ^**H**^ **6** ^**2+**^ (with PF_6_^–^ counter-anions) was prepared via oxidation of ^**H**^ **12H**_**6**_^**2+**^ (with ClO_4_^–^ counter-anions) with O_2_ followed by counteranion exchange with NH_4_PF_6_. Complexes ^**H**^ **8H**_**2**_^**2+**^ and ^**H**^ **10H**_**4**_^**2+**^ were prepared in small quantities (∼10 mg) by reacting ^**H**^ **6** ^**2+**^ with stoichiometric amounts of hydrazine in THF.

For consistency, we decided to prepare all the iron complexes included in this paper using ClO_4_^–^ as counteranion. The fully reduced-protonated Fe complexes ^**H**^ **12H**_**6**_^**2+**^, ^**Me**^ **12H**_**6**_^**2+**^ and ^**MeO**^ **12H**_**6**_^**2+**^ were obtained by mixing Fe^II^(ClO_4_)_2_·6H_2_O with 3 equiv of the corresponding opda ligand (4,5-X-phenylenediamine, X = H, Me, and MeO) under anaerobic conditions in THF (Figure 3).^23^ Addition of the Fe^II^(ClO_4_)_2_·6H_2_O to the ligand led to precipitation of the desired ^**X**^ **12H**_**6**_^**2+**^ complexes as colorless powders (see Supporting Information for further details). Crystalline material suitable for single-crystal X-ray diffraction analysis (SC-XRD) was obtained by dissolving ^**X**^ **12H**_**6**_^**2+**^ in CH_3_CN and layering Et_2_O (see SC-XRD structure of ^**Me**^ **12H**_**6**_^**2+**^ in Figure 3).

**Figure 3:**
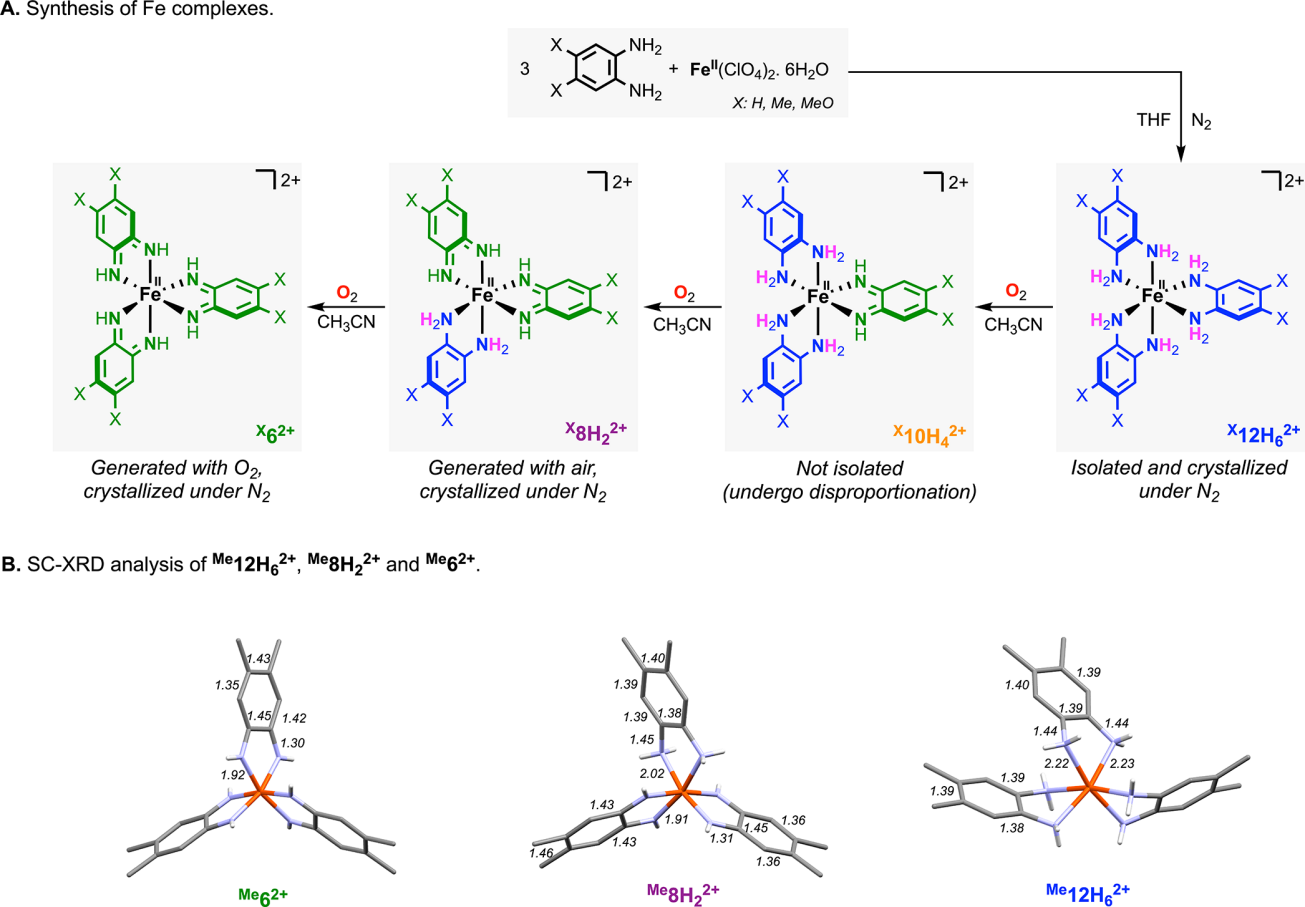
Synthesis and structures of Fe complexes. (A) Synthesis of Fe complex **1a**–**1c**, **3a**–**3c**, and **4a**–**4c**; (B) crystal structures of **1b**, **3b**, and **4b**. Note: the ClO_4_^–^ counter-anions, lattice solvent molecules, and some of the hydrogen atoms were omitted for clarity.

The ^**X**^ **12H**_**6**_^**2+**^ complexes were used as starting materials to synthesize ^**X**^ **10H**_**4**_^**2+**^, ^**X**^ **8H**_**2**_^**2+**^, and ^**X**^ **6** ^**2+**^ using O_2_ as oxidant. Attempts to isolate ^**X**^ **10H**_**4**_^**2+**^ were unsuccessful due to the tendency of these species to disproportionate to produce ^**X**^ **8H**_**2**_^**2+**^ and ^**X**^ **12H**_**6**_^**2+**^. The fully oxidized-deprotonated Fe complexes (^**H**^ **6** ^**2+**^, ^**Me**^ **6** ^**2+**^ and ^**MeO**^ **6** ^**2+**^) were prepared by bubbling O_2_ to CH_3_CN solutions of the corresponding ^**X**^ **12H**_**6**_^**2+**^ complex (Figure 3). The oxidation reactions were monitored by NMR, in which the formation of ^**X**^ **8H**_**2**_^**2+**^, ^**X**^ **10H**_**4**_^**2+**^ and ^**X**^ **12H**_**6**_^**2+**^ could be observed (see sections below for further discussion). Once we ensured that ^**X**^ **6** ^**2+**^ were fully formed, Et_2_O was added to the CH_3_CN solutions to precipitate the complexes (see Supporting Information). The resulting materials were recrystallized in CH_3_CN by Et_2_O vapor diffusion and analyzed by SC-XRD (see ^**Me**^ **6** ^**2+**^ in Figure 3).

The partially reduced complexes ^**H**^ **8H**_**2**_^**2+**^, ^**Me**^ **8H**_**2**_^**2+**^, and ^**MeO**^ **8H**_**2**_^**2+**^ were prepared by exposing CH_3_CN solutions of the corresponding ^**X**^ **12H**_**6**_^**2+**^ complex to air. The reactions were monitored by NMR to ensure the full formation of the ^**X**^ **8H**_**2**_^**2+**^ complexes and were precipitated by adding Et_2_O before the generation of ^**X**^ **6** ^**2+**^ (see Supporting Information). The resulting precipitates were recrystallized in CH_3_CN by Et_2_O vapor diffusion, and the crystals analyzed by SC-XRD (see Figure 3).

The SC-XRD structures of ^**Me**^ **12H**_**6**_^**2+**^, ^**Me**^ **8H**_**2**_^**2+**^ and ^**Me**^ **6** ^**2+**^ are depicted in Figure 3B (the SC-XRD structures for ^**H**^ **12H**_**6**_^**2+**^, ^**H**^ **8H**_**2**_^**2+**^, ^**Me**^ **6** ^**2+**^, ^**MeO**^ **12H**_**6**_^**2+**^, ^**MeO**^ **8H**_**2**_^**2+**^ and ^**MeO**^ **6** ^**2+**^ can be found in the Supporting Information). The unit cell of all the complexes contained one or more crystallization solvent molecules (THF or CH_3_CN) not coordinated to the iron center, as well as 2 perchlorate cations, which confirmed the dicationic nature of the iron complexes (see Supporting Information for further details). Analysis of the ligand C–N, C–C distances in complex ^**Me**^ **6** ^**2+**^ is consistent with its formulation as Fe^II^ complex bound by three quinone-like ligands (i.e., [Fe^II^(^q^L)_3_]^2+^). In ^**Me**^ **8H**_**2**_^**2+**^, the reductive protonation of one of the ligands to the catechol-like form induced dramatic changes in the C–C and C–N distances. In ^**H**^ **12H**_**6**_^**2+**^, the C–C and C–N distances measured for the three ligands are consistent with the formulation of this complex as an iron­(II) species bound by three catechol-like ligands (i.e., i.e., [Fe^II^(^cat^LH_2_)_3_]^2+^). Analysis of the Fe–N distances for the three complexes (^**Me**^ **12H**_**6**_^**2+**^, ^**Me**^ **8H**_**2**_^**2+**^ and ^**Me**^ **6** ^**2+**^) provide evidence on the oxidation state of the ligands and the spin state of the metal complexes. The Fe–N­(^q^L) distances in ^**Me**^ **6** ^**2+**^ are similar to the ones recorded in ^**Me**^ **8H**_**2**_^**2+**^ (1.92 vs 1.91 Å). Conversely, the Fe–N­(^cat^LH_2_) distances recorded for ^**Me**^ **8H**_**2**_^**2+**^ were substantially shorter than the ones found in ^**Me**^ **12H**_**6**_^**2+**^(2.02 vs 2.22 Å). These observations agree with the formulation of ^**Me**^ **6** ^**2+**^ and ^**Me**^ **8H**_**2**_^**2+**^ as low-spin ferrous complexes and ^**Me**^ **12H**_**6**_^**2+**^ as a high-spin species. Similar structural trends were observed for the MeO and H-substituted systems (see Supporting Information). The change in spin state for the ^**H**^ **12H**_**6**_^**2+**^ was further corroborated by NMR and Mössbauer measurements (see sections below).

### NMR Characterization of the Fe Complexes

The Fe complexes ^**X**^ **6** ^**2+**^, ^**X**^ **8H**_**2**_^**2+**^, ^**X**^ **10H**_**4**_ **^2+^,** and ^**X**^ **12H**_**6**_^**2+**^ were characterized by ^1^H NMR spectroscopy in CD_3_CN (see Figure 4 and Supporting Information). The NMR spectra of the diamagnetic complexes ^**X**^ **6** ^**2+**^ and ^**X**^ **8H**_**2**_^**2+**^ species were recorded from the isolated complexes. The spectra of the ^**X**^ **12H**_**6**_^**2+**^ complexes depicted broad peaks characteristic of a paramagnetic octahedral iron­(II) complex (*S* = 2). The paramagnetism of ^**Me**^ **12H**_**6**_^**2+**^ was confirmed by recording the NMR at different temperatures, which cause significant shifts and sharpening of the paramagnetic signals (see Supporting Information). The NMR spectra of ^**Me**^ **12H**_**6**_^**2+**^ at different concentrations in the presence of an internal standard were recorded, and the results suggested that the ^**X**^ **12H**_**6**_^**2+**^ complexes could be quantified by NMR in the 2–10 mM range despite their paramagnetism (see Supporting Information).

**Figure 4:**
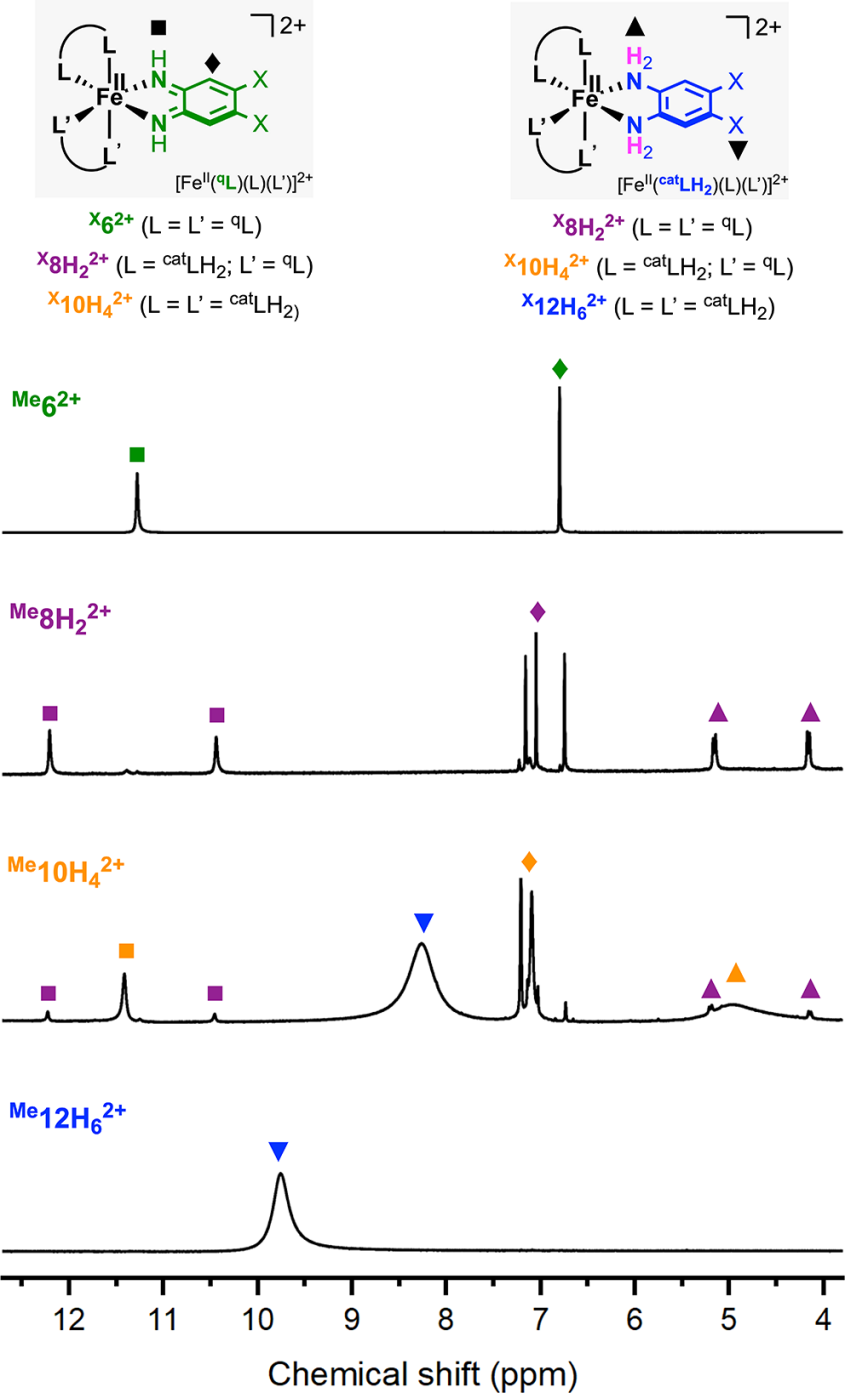
^1^H NMR spectra of ^**Me**^ **6** ^**2+**^, ^**Me**^ **8H**_**2**_^**2+**^, ^**Me**^ **10H**_**4**_^**2+**^ and ^**Me**^ **12H**_**6**_^**2+**^ (from top to bottom) recorded in CD_3_CN at room temperature. Note: The NMR spectrum of pure ^**Me**^ **10H**_**4**_^**2+**^ could not be obtained due to the tendency of this complex to disproportionate and form mixtures (see peaks corresponding to ^**Me**^ **8H**_**2**_^**2+**^ and ^**Me**^ **12H**_**6**_^**2**^ in the spectrum of ^**Me**^ **10H**_**4**_^**2+**^).

The NMR spectra of the diamagnetic complexes ^**X**^ **10H**_**4**_^**2+**^ was obtained by analyzing isolated solid materials derived from the oxygenation of ^**X**^ **12H**_**6**_^**2+**^ at short reaction times, which contained mixtures of ^**X**^ **12H**_**6**_^**2+**^, ^**X**^ **10H**_**4**_^**2+**^, ^**X**^ **8H**_**2**_ **^2+^,** and ^**X**^ **6** ^**2+**^ (see Supporting Information for further details). Our results are also in agreement with the NMR data recently reported by Chang and coworkers for the complexes ^**H**^ **12H**_**6**_^**2+**^, ^**H**^ **10H**_**4**_^**2+**^, ^**H**^ **8H**_**2**_ **^2+^,** and ^**H**^ **6** ^**2+**^, which was obtained in THF-*d*_8_ (see Supporting Information).^23^ The paramagnetism of ^**X**^ **12H**_**6**_^**2+**^ was confirmed by the Evans method (see details in Supporting Information).

### UV–Vis Characterization of the Fe Complexes

The UV–vis spectra of ^**X**^ **6** ^**2+**^, ^**X**^ **8H**_**2**_^**2+**^, ^**X**^ **10H**_**4**_ **^2+^,** and ^**X**^ **12H**_**6**_^**2+**^ were measured in CH_3_CN at room temperature (see Figure 5 and Supporting Information). The spectra of ^**X**^ **10H**_**4**_^**2+**^ was obtained by analyzing the isolated solid materials derived from the oxygenation of ^**X**^ **12H**_**6**_^**2+**^ at short reaction times, which contained mixtures of ^**X**^ **12H**_**6**_^**2+**^, ^**X**^ **10H**_**4**_ **^2+^,** and ^**X**^ **8H**_**2**_^**2+**^. The spectrum of ^**X**^ **10H**_**4**_^**2+**^ could be simulated by subtracting the spectra of ^**X**^ **8H**_**2**_^**2+**^ and ^**X**^ **6** ^**2+**^ based on the complex ratios observed by NMR (see further details in the Supporting Information). The spectra of ^**H**^ **12H**_**6**_^**2+**^, ^**H**^ **10H**_**2**_^**2+**^, ^**H**^ **8H**_**2**_ **^2+^,** and ^**H**^ **6** ^**2+**^ complexes agreed with the ones reported by Chang and coworkers.^23^ The low-spin complexes ^**X**^ **10H**_**4**_^**2+**^, ^**X**^ **8H**_**2**_ **^2+^,** and ^**X**^ **6** ^**2+**^ depicted strong absorption bands in the visible region that are assigned as metal-to-ligand charge transfer bands (MLCT) from the iron­(II) to the quinone-like ligands. Conversely, the high-spin complexes ^**X**^ **12H**_**6**_^**2+**^ depicted weak absorption bands in the visible range, assigned as spin-allowed d-d transitions. Variations on the ligand substituent in ^**X**^ **6** ^**2+**^, ^**X**^ **8H**_**2**_ **^2+^,** and ^**X**^ **10H**_**4**_^**2+**^ (X: H, Me, MeO) led to slight shifts of the MLCT absorption bands and substantial changes on the molar extinction coefficients, associated with the donating ability of the substituent (λ_max_: MeO > Me > H; ε: MeO > Me > H).^24^

**Figure 5:**
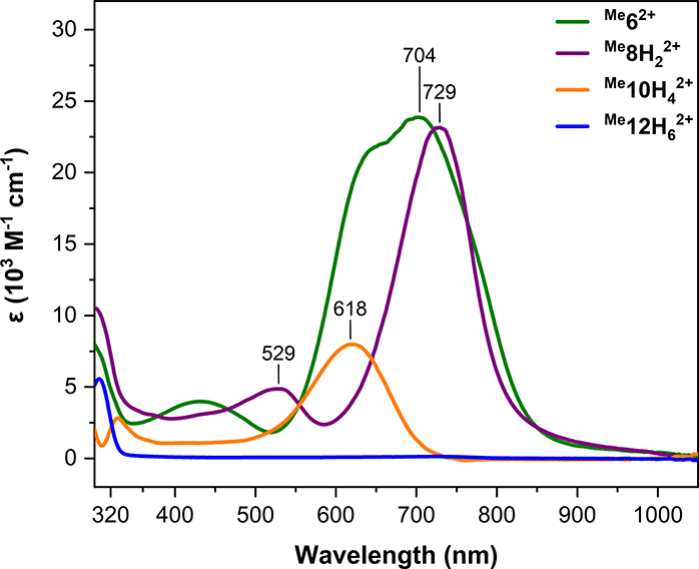
UV–vis spectra of complexes ^**Me**^ **6** ^**2+**^, ^**Me**^ **8H**_**2**_^**2+**^, ^**Me**^ **10H**_**4**_ **^2+^,** and ^**Me**^ **12H**_**6**_^**2+**^ (see Supporting Information for further details).

### Mössbauer Characterization of the Fe Complexes

The electronic structure of the Fe complexes was also analyzed by ^57^Fe Mössbauer (Mb) spectroscopy (Figure 6). Complex ^**Me**^ **12H**_**6**_^**2+**^ exhibits a quadrupole doublet under low-temperature-low-field condition, with an isomer shift (δ) of 1.21 mm/s and a quadrupole splitting (|Δ*E*_Q_|) of 2.01 mm/s, which are typical values of high-spin ferrous ions in octahedral ligand environment. In contrast, ^**Me**^ **8H**_**2**_^**2+**^ shows drastic changes compared to ^**Me**^ **12H**_**6**_^**2+**^– both δ and |Δ*E*_Q_| decreases to 0.16 and 1.43 mm/s, respectively, reminiscent of the parameters reported for a series of octahedral low-spin (*S* = 0) ferrous-dioximato complexes.^25^ The low-temperature-high-field spectrum further confirmed the diamagnetic properties of ^**Me**^ **8H**_**2**_^**2+**^, and determined the sign of quadrupole splitting to be negative, yielding Δ*E*_Q_ = −1.43 mm/s with the asymmetric parameter η = 0.7. Similarly, for the fully oxidized species, ^**Me**^ **6** ^**2+**^, parameters remain in the range for low-spin ferrous ions, which are δ = 0.09 mm/s, Δ*E*_Q_ = −1.00 mm/s and η = 0.4, while the slightly decreased Δ*E*_Q_ and η compared to the ^**Me**^ **8H**_**2**_^**2+**^ can be attributed to the more symmetric ligand field in ^**Me**^ **6** ^**2+**^ as all three bidentate ligands have been oxidized, consistent with the crystallographic results.

**Figure 6:**
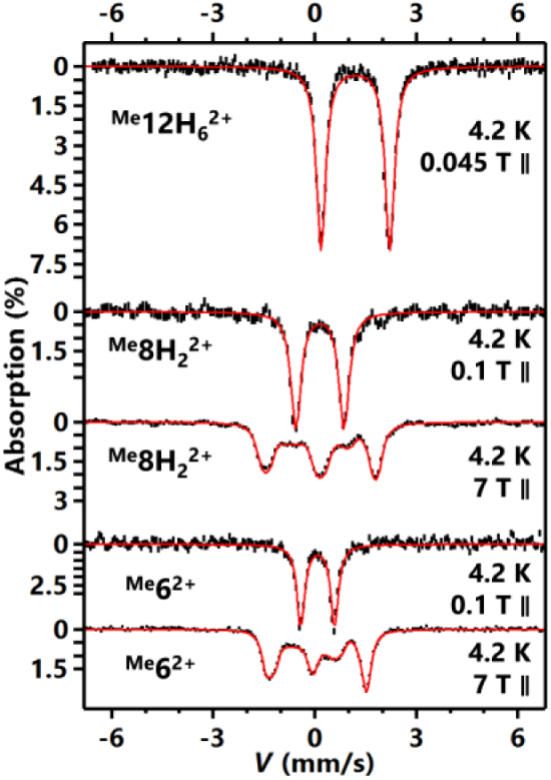
^57^Fe Mössbauer spectra for complexes ^**Me**^ **12H**_**6**_^**2+**^, ^**Me**^ **8H**_**2**_^**2+**^, and ^**Me**^ **6** ^**2+**^ under the measurement conditions indicated in the figure. The external static magnetic field is parallel to the γ-radiation. Black bars represent experimental uncertainties, while red lines show simulations. The simulation parameters are δ = 1.21 mm/s, |Δ*E*_Q_| = 2.01 mm/s for ^**Me**^ **12H**_**6**_^**2+**^; *S* = 0, δ = 0.16 mm/s, Δ*E*_Q_ = −1.43 mm/s, η = 0.7 for ^**Me**^ **8H**_**2**_^**2+**^; *S* = 0, δ = 0.09 mm/s, Δ*E*_Q_ = −1.00 mm/s, η = 0.4 for ^**Me**^ **6** ^**2+**^.

In addition, substituents effects on Mb parameters have been investigated for ^**X**^ **12H**_**6**_^**2+**^ and ^**X**^ **6** ^**2+**^ (X = H, Me, MeO; see Supporting Information). The δ values for ^**X**^ **6** ^**2+**^ species exhibit a positive shift (δ = 0.08, 0.09, 0.13 mm/s for H, Me, MeO, respectively) with increasing electron-donating strength of the substituents (MeO > Me ≈ H), an expected results due to the increase of the Fe 3d orbital electron population. However, for ^**X**^ **12H**_**6**_^**2+**^, the order of isomer shift values is Me > H ≈ MeO (δ = 1.21, 1.12, 1.13 mm/s for H, Me, MeO, respectively). Nevertheless, the variation of parameters within the same oxidation states is small, suggesting the Mb parameters mainly determined by the iron oxidation states.

### Stepwise Oxidative Deprotonation of ^Me^H_6_^2+^ to ^Me^6^2+^

The stepwise 6H^+^/6e^–^ reactivity of the Fe-based ECPB systems was confirmed by performing the oxidative deprotonation of complex ^**Me**^ **12H**_**6**_^**2+**^ with dioxygen (see Figure S27). The reactions were followed by ^1^H NMR in CD_3_CN to quantify all species (^**Me**^ **12H**_**6**_^**2+**^, ^**Me**^ **10H**_**4**_^**2+**^, ^**Me**^ **8H**_**2**_^**2+**^ and ^**Me**^ **6** ^**2+**^) involved in the reaction. First, ^**Me**^ **12H**_**6**_^**2+**^ was rapidly oxidized to form ^**Me**^ **10H**_**4**_^**2+**^ and ^**Me**^ **8H**_**2**_^**2+**^ (instantaneous color change from colorless to navy blue was observed). After ∼0.5 h, the concentration of ^**Me**^ **10H**_**4**_^**2+**^ started to decrease, ^**Me**^ **8H**_**2**_^**2+**^ continued accumulating; and ^**Me**^ **6** ^**2+**^ was observed in the spectrum (*t* = 7.9 h). ^**Me**^ **12H**_**6**_^**2+**^ and ^**Me**^ **10H**_**4**_^**2+**^ were fully consumed after ∼9 h. After that, ^**Me**^ **8H**_**2**_^**2+**^ was slowly oxidized to ^**Me**^ **6** ^**2+**^ (i.e., the last oxidation step took a couple of weeks).

Overall, the conversion of ^**Me**^ **12H**_**6**_^**2+**^ to ^**Me**^ **6** ^**2+**^ occurred with good yields (∼70%) and maintaining a good ECPB mass balance (ECPB mass balance = ([^**X**^ **6** ^**2+**^] + [^**X**^ **8H**_**2**_^**2+**^] + [^**X**^ **10H**_**4**_^**2+**^] + [^**X**^ **12H**_**6**_^**2+**^])/[^**X**^ **12H**_**6**_^**2+**^]_0_ × 100) throughout the reaction. The 6H^+^/6e^–^ oxidative deprotonation of ^**Me**^ **12H**_**6**_^**2+**^ to ^**Me**^ **6** ^**2+**^ occurred in a stepwise fashion, with accumulation and decay of ^**Me**^ **8H**_**2**_^**2+**^and ^**Me**^ **10H**_**4**_^**2+**^. However, we noticed that ^**Me**^ **10H**_**4**_^**2+**^ reached lower concentrations for shorter periods of times (up to 3 mM in a period of 9 h) when compared to ^**Me**^ **8H**_**2**_^**2+**^ (up to 8 mM for more than 300 h). We believe that ^**Me**^ **10H**_**4**_^**2+**^ undergoes a disproportionation reaction to form ^**Me**^ **8H**_**2**_^**2+**^and ^**Me**^ **12H**_**6**_^**2+**^. We will discuss the disproportionation reactions in detail in the sections below.

The oxygenation of ^**H**^ **12H**_**6**_^**2+**^ and ^**MeO**^ **12H**_**6**_^**2+**^ was also followed by NMR (see Supporting Information). When compared to ^**Me**^ **12H**_**6**_^**2+**^, the reactions were found to be slightly slower. The aerobic oxidation ^**H**^ **12H**_**6**_^**2+**^ and ^**MeO**^ **12H**_**6**_^**2+**^ systems also led poorer ECPB mass balances, which can be explained by the formation of oxidation products derived from *ortho*-phenylenediamine degradation (see Supporting Information for further details).

### Stepwise Reductive Protonation of ^Me^6^2+^ to ^Me^12H_6_^2+^

The low bond dissociation free energy of N_2_H_4_ (BDFE_avg_(4H^+^/4e^–^) = 39.1 kcal/mol in gas phase) suggests that it can be utilized as strong PCET reductant. In previous reports, Chang and coworkers used stoichiometric amounts of N_2_H_4_ to prepare ^**H**^ **8H**_**2**_^**2+**^, ^**H**^ **10H**_**4**_ **^2+^,** and ^**H**^ **12H**_**6**_^**2+**^ using ^**H**^ **6** ^**2+**^ as starting material.^23^ Mayer and coworkers also carried out the 2H^+^/2e^–^ reduction of a Ru^IV^-amido complex to the corresponding Ru^II^-amine complex using N_2_H_4_.^26^ The stepwise reductive protonation of ^**Me**^ **6** ^**2+**^ to ^**Me**^ **8H**_**2**_^**2+**^, ^**Me**^ **10H**_**4**_^**2+**^, and ^**Me**^ **12H**_**6**_^**2+**^ was followed by ^1^H NMR in CD_3_CN (see Figure S30). The reaction was carried out by titrating substoichiometric amounts of N_2_H_4_, which allowed for observing the ^**Me**^ **8H**_**2**_^**2+**^and ^**Me**^ **10H**_**4**_^**2+**^. The sequential reductive protonation of ^**Me**^ **6** ^**2+**^ with 1 equiv of N_2_H_4_ led to the formation of ^**Me**^ **8H**_**2**_^**2+**^ and ^**Me**^ **10H**_**4**_^**2+**^, with the latter being the main product. The full reduction of ^**Me**^ **8H**_**2**_^**2+**^ and ^**Me**^ **10H**_**4**_^**2+**^ was accomplished upon adding 1.5 to 2.0 equiv of N_2_H_4_ (i.e., the stoichiometry of the reaction indicates that 1.5 equiv of N_2_H_4_ are required to reduce ^**Me**^ **6** ^**2+**^ to ^**Me**^ **12H**_**6**_^**2+**^). To our surprise, the peaks corresponding to ^**Me**^ **12H**_**6**_^**2+**^ shifted during the reaction and the peak integration diminished drastically. We speculated that under the reaction conditions, the high-spin ^**Me**^ **12H**_**6**_^**2+**^ complex exchanged with the excess of N_2_H_4_, broadening the NMR peaks. In fact, addition of N_2_H_4_ to a solution of the isolated ^**Me**^ **12H**_**6**_^**2+**^ led to similar NMR changes (broadening and shifting of NMR peaks and diminishing of signal integration). To analyze the mass balance of the reaction we added 3 equiv of 4,4’-bipyridine (bpy) to the final ^**Me**^ **12H**_**6**_^**2+**^/N_2_H_4_ solution, which led to the formation of the low-spin [Fe­(bpy)_3_]^2+^ (∼80%) and 4,5-Me_2_-opda ligand (∼80%).

The reductive protonation of ^**H**^ **6** ^**2+**^and ^**MeO**^ **6** ^**2+**^ with N_2_H_4_ was also followed by NMR spectroscopy (see Supporting Information). Like in the reduction of ^**Me**^ **6** ^**2+**^, ^**H**^ **6** ^**2+**^ was rapidly reduced upon addition of N_2_H_4_, leading to the appearance of paramagnetic species associated with the generation of ^**H**^ **12H**_**6**_^**2+**^. Conversely, the reaction of ^**MeO**^ **6** ^**2+**^ with N_2_H_4_ was extremely slow and required additional equivalents of N_2_H_4_ for complete reduction (see Supporting Information). The mass balance of the reactions was calculated by adding 3 equiv of bpy, which led to the formation of [Fe­(bpy)_3_]^2+^ and the corresponding “free” opda ligands (∼70% for ^**H**^ **6** ^**2+**^ and ∼60% for ^**MeO**^ **6** ^**2+**^).

### BDFE Determination of the Fe-ECPB Systems: Reaction with PCET Reagents

The thermochemical tendency for a species to accept H· equivalents can be determined with the Bordwell equation, in which the BDFE of the bond formed during the reductive protonation of the oxidant is related to its basicity (p*K*_*a*_) and its redox potential (*E*_1/2_, see Figure 7).^1^ For reactions involving more than one PCET event, the stepwise BDFE values lead to an average BDFE value (BDFE_avg_) that can be experimentally obtained by open-circuit potential (OCP) measurements, a methodology recently reported by Mayer and coworkers.^21^ The conversion between ^**X**^ **6** ^**2+**^ and ^**X**^ **12H**_**6**_^**2+**^ involved a minimum of seven Fe species (namely ^**X**^ **6** ^**2+**^, ^**X**^ **7H** ^**2+**^, ^**X**^ **8H**_**2**_^**2+**^, ^**X**^ **9H**_**3**_^**2+**^, ^**X**^ **10H**_**4**_^**2+**^, ^**X**^ **11H**_**5**_ **^2+^,** and ^**X**^ **12H**_**6**_^**2+**^) and six 1H^+^/1e^–^ PCET events with a BDFE associated with each one of them (BDFE_1_ to BDFE_6_, see Figure 7). In the PCET reactivity of the Fe-ECPB systems reported in this article, we observed the formation only four iron species (namely ^**X**^ **6** ^**2+**^, ^**X**^ **8H**_**2**_^**2+**^, ^**X**^ **10H**_**4**_ **^2+^,** and ^**X**^ **12H**_**6**_^**2+**^) all bound by the catechol-like and quinone-like forms of the ligand (^cat^LH_2_ and ^q^L, see Figure 7). The iron semiquinone-like species derived from the 1H^+^/1e^–^ reductive protonation and/or oxidative deprotonation of ^**X**^ **6** ^**2+**^, ^**X**^ **8H**_**2**_^**2+**^, ^**X**^ **10H**_**4**_ **^2+^,** and ^**X**^ **12H**_**6**_^**2+**^ (namely ^**X**^ **7H** ^**2+**^, ^**X**^ **9H**_**3**_^**2+**^, ^**X**^ **11H**_**5**_^**2+**^) are proposed to be formed during the PCET transformations but do not accumulate in solution due to the propensity to undergo fast disproportionation reactions. Hence, we can study the PCET reactivity of the Fe-based ECPBs by determining the thermochemistry of the 2H^+^/2e^–^ PCET couples ^**X**^ **6** ^**2+**^/^**X**^ **8H**_**2**_^**2+**^ (BDFE_avg1,2_), ^**X**^ **8H**_**2**_^**2+**^/^**X**^ **10H**_**4**_^**2+**^ (BDFE_avg3,4_) and ^**X**^ **10H**_**4**_^**2+**^/^**X**^ **12H**_**6**_^**2+**^ (BDFE_avg5,6_), from which we can obtain the thermochemistry of the overall 6H^+^/6e^–^ transformation from ^**X**^ **6H** ^**2+**^ to ^**X**^ **12H**_**6**_^**2+**^ (BDFE_avg1,6_, see Figure 7).

**Figure 7:**
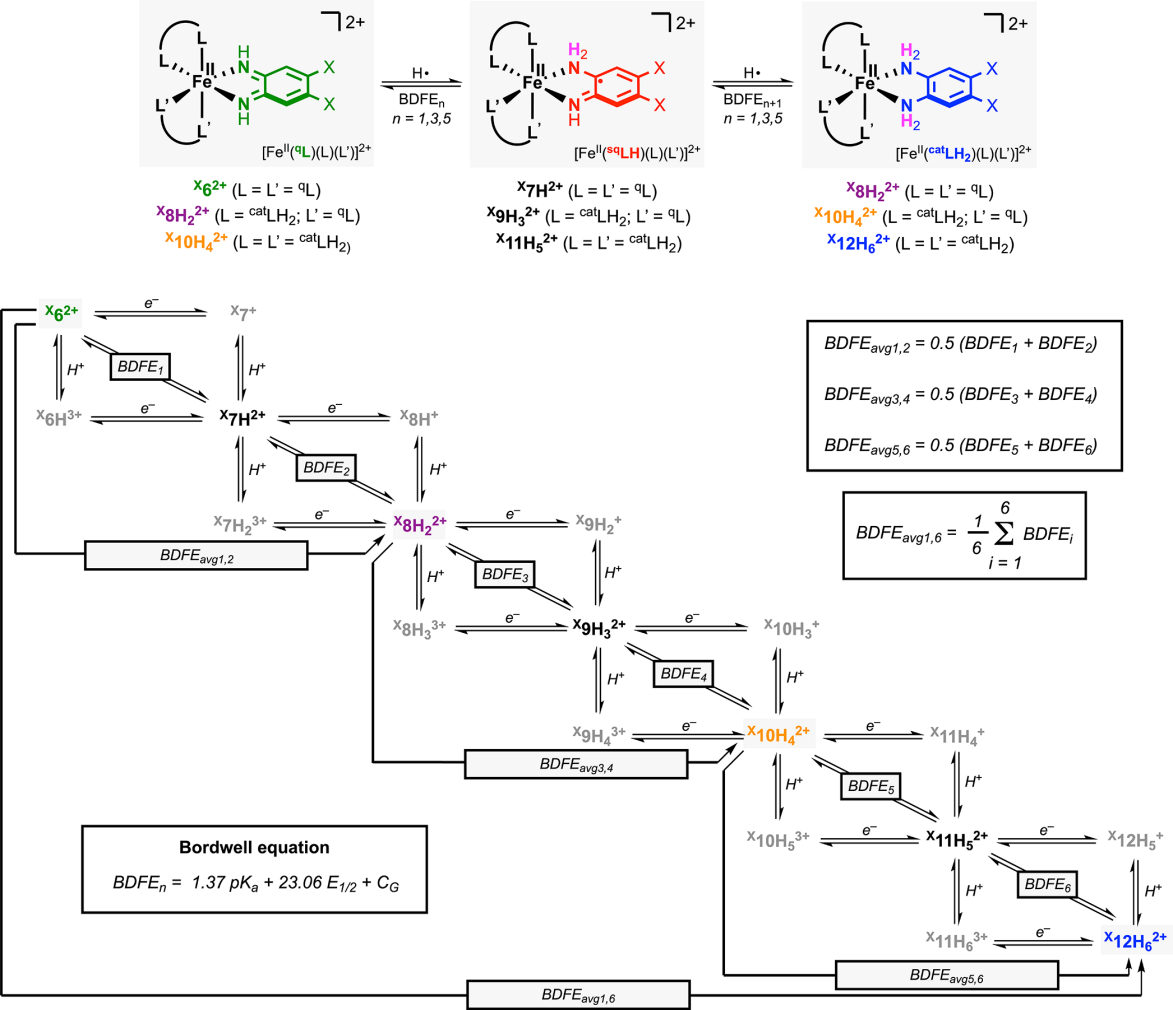
BDFE analysis for the species involved in the Fe-based ECPB systems.

To determine the BDFE_avg_ of the ^**X**^ **6** ^**2+**^/^**X**^ **8H**_**2**_^**2+**^, ^**X**^ **8H**_**2**_^**2+**^/^**X**^ **10H**_**4**_ **^2+^,** and ^**X**^ **10H**_**4**_^**2+**^/^**X**^ **12H**_**6**_^**2+**^ couples, we carried out the reaction of ^**X**^ **6** ^**2+**^ with PCET reagents with known BDFE such as substituted hydroquinones (2,6-X_2_-1,4-H_2_Q) and diphenylhydrazine (PhNHNHPh) (see Scheme 1 and Table 1).^1,20^ The reactions were followed by NMR spectroscopy using excess amounts of the PCET reagent (∼10 equiv), which allowed for determining the mass balance of the transformation based on the Fe complex and the PCET reagent (see Supporting Information for further experimental details). To our surprise, we observed that some of the PCET reagents would lead to partial reductive protonation of ^**X**^ **6** ^**2+**^ to ^**X**^ **8H**_**2**_^**2+**^ while others would lead to full formation of ^**X**^ **12H**_**6**_^**2+**^. For example, ^**Me**^ **6** ^**2+**^ did not react with 1,4-H_2_Q (BDFE_avg_ = 67.3 kcal/mol), was partially reduced to ^**Me**^ **8H**_**2**_^**2+**^ by 2,6-Me_2_-1,4-H_2_Q (BDFE_avg_ = 64.6 kcal/mol) and was reduced to ^**Me**^ **12H**_**6**_^**2+**^ by 2,6-(MeO)_2_-1,4-H_2_Q (BDFE_avg_ = 62.8 kcal/mol). These results suggests that the BDFE_avg_ of the ^**Me**^ **6** ^**2+**^ **/** ^**Me**^ **8H**_**2**_^**2+**^ couple is higher than 64.6 kcal/mol, while the BDFE_avg_ value for the ^**Me**^ **8H**_**2**_^**2+**^/^**Me**^ **10H**_**4**_^**2+**^ couple substantially lower than 64.6 kcal/mol. A similar behavior was observed for ^**H**^ **6** ^**2+**^ and ^**MeO**^ **6** ^**2+**^ (i.e., partial reduction with some PCET reagents and full reduction with others), with ^**H**^ **6** ^**2+**^ requiring PCET reagents with higher BDFE_avg_ than ^**Me**^ **6** ^**2+**^, and ^**MeO**^ **6** ^**2+**^ requiring PCET reagents with lower BDFE_avg_ than ^**Me**^ **6** ^**2+**^ (see Supporting Information for details).

**Scheme 1:**
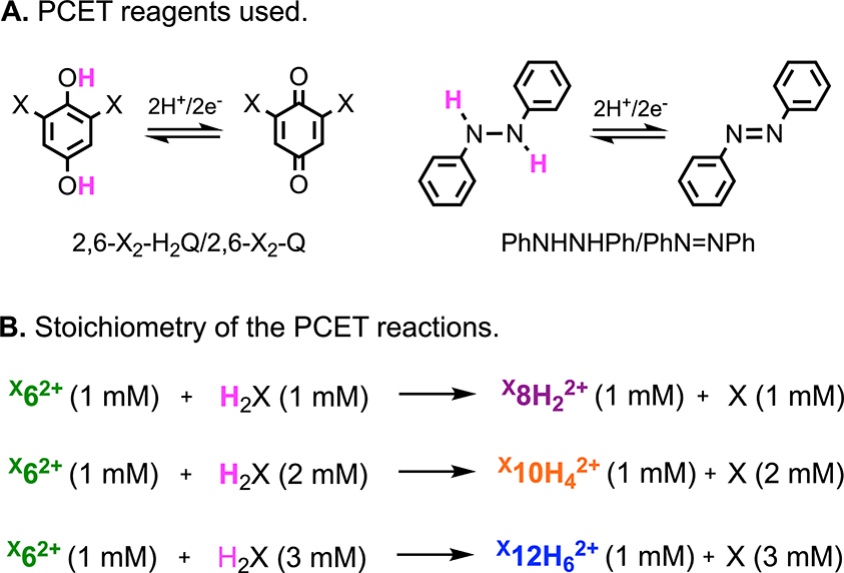
(A) PCET Reagents Used in the Reactivity Experiments. (B) Stoichiometry of the PCET Reactions

**Table 1:** Reactivity between the ^X^6^2+^ and PCET Reagents with Known BDFE_avg_ Values in CH_3_CN^1^ or DMF^20^ (See Supporting Information for Additional Details)

PCET reagent	BDFE_avg_	**^H^6^2+^**	**^Me^6^2+^**	**^MeO^6^2+^**
2,6-Cl_2_–H_2_Q	68.9^DMF^	n.r.	n.r.	n.r.
H_2_Q	67.3^ACN^	to ^**H**^ **8H**_**2**_^**2+**^	n.r.	n.r.
2,6-Me_2_–H_2_Q	64.6^ACN^	to ^**H**^ **12H**_**6**_^**2+**^	to ^**Me**^ **8H**_**2**_^**2+**^	n.r.
2,6-(MeO)_2_–H_2_Q	62.8^ACN^	to ^**H**^ **12H**_**6**_^**2+**^	to ^**Me**^ **12H**_**6**_^**2+**^	to ^**MeO**^ **8H**_**2**_^**2+**^
PhNHNHPh	60.9^ACN^	to ^**H**^ **12H**_**6**_^**2+**^	to ^**Me**^ **12H**_**6**_^**2+**^	to ^**MeO**^ **12H**_**6**_^**2+**^

### BDFE Determination of the Fe-ECPB Systems: Open Circuit Potential (OCP) Measurements

Mayer and coworkers have recently reported that the BDFE values of PCET couples obtained from OCP measurements are more reliable than the results obtained by traditional cyclic voltammetry in nonaqueous solvents.^21^ Moreover, OCP measurements can be applied to PCET couples separated by more than one proton and one electron, like hydroquinone/quinone couples. Since then, OCP measurements have not only been applied in the analysis of the BDFE_avg_ of organic PCET substates,^21^ but also for the BDFE determination of metal complexes^19,21^ and polyoxometalates.^27,28^ OCPs (E^o^, V vs H_2_) are electrochemical measurements carried out at different relative concentrations of the protonated/reduced form of the PCET reagent (H_n_X) and deprotonated/oxidized form (X), leading to linear plots that allow for determining the BDFE_avg_ of the PCET couples (intercept: potential (E^o^, V vs H_2_) when [H_n_X] = [X], BDFE = 23.06E^o^ + ΔG­(H·)) and the number of protons and electrons involved in the transformation (slope = −0.0592 V/n).

We have recently shown that OCP measurements can be performed to determine the BDFE_avg_ of 4H^+^/4e^–^ PCET couples of Cu-based ECPBs.^19,20^ For the Cu-based ECPBs, we observed OCP slopes characteristic of 4H^+^/4e^–^ transformations (∼−0.015; theoretical: −0.0592/4). The intercept of the OCP measurements depended on the Cu-based ECPB analyzed, consistent with the variations on the BDFE_avg_ of 4H^+^/4e^–^ upon ligand substitution (see Figure 2). We proposed that the ability of the Cu-ECPB systems to undergo fast 1H^+^/1e^–^ events to regenerate the equilibrium between the fully deprotonated/oxidized state (^**X,R**^ **5** ^**+**^) and the fully protonated/reduced state ^**X,R**^ **1H**_**4**_^**+**^ led to fast equilibration of the system and allowed for determining the BDFE_avg_ of the 4H^+^/4e^–^ process (Figure 8).

**Figure 8:**
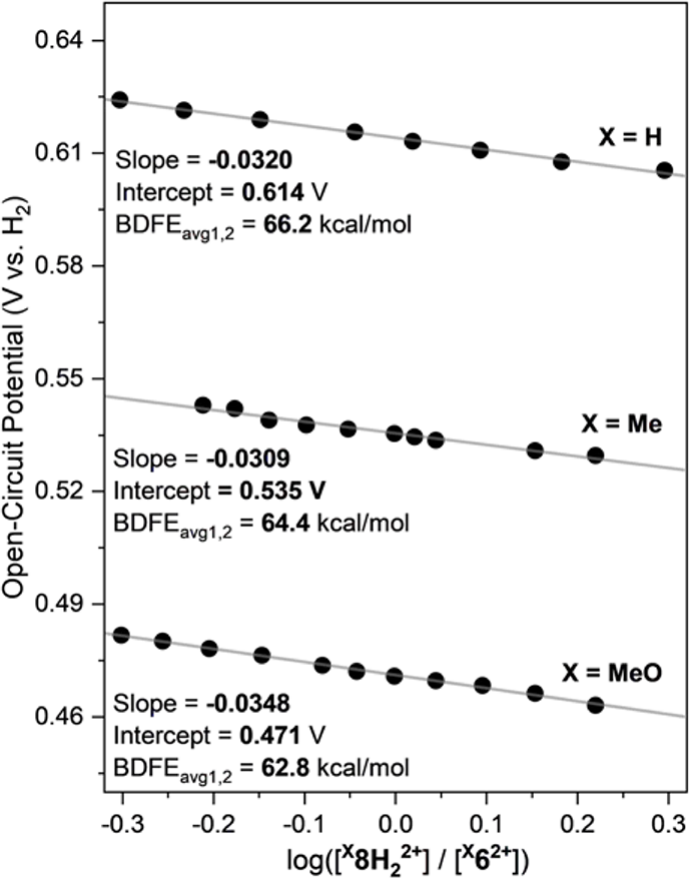
Open-circuit measurements of the ^**X**^ **6** ^**2+**^/^**X**^ **8H**_**2**_^**2+**^ couples (see Supporting Information for additional details).

For the Fe systems described herein, we were able to measure the OCPs for the ^**X**^ **6** ^**2+**^/^**X**^ **8H**_**2**_^**2+**^ couples (Figure 8). Attempts to measure the OCPs for the ^**X**^ **6** ^**2+**^/^**X**^ **12H**_**2**_^**2+**^ couples were unsuccessful due to the formation of mixtures of ^**X**^ **6** ^**2+**^, ^**X**^ **8H**_**2**_^**2+**^, ^**X**^ **10H**_**4**_ **^2+^,** and ^**X**^ **12H**_**2**_^**2+**^ (see section below for further details). Similarly, the OCPs of the ^**X**^ **8H**_**2**_^**2+**^/ ^**X**^ **12H**_**2**_^**2+**^couples could not be measured either due to the formation of substantial amounts of ^**X**^ **10H**_**4**_^**2+**^ in solution. The slope of the OCP measurements for the ^**X**^ **6** ^**2+**^/^**X**^ **8H**_**2**_^**2+**^ couples was consistent with 2H^+^/2e^–^ events (∼−0.030; theoretical: −0.0592/2). The BDFE_avg1,2_ of the ^**X**^ **6** ^**2+**^/^**X**^ **8H**_**2**_^**2+**^ couples obtained by OCP (66.2 kcal/mol for X = H; 64.4 kcal/mol for X = Me; and 62.8 kcal/mol for X = MeO) agreed with the PCET reactivity experiments described above in which ^**H**^ **6** ^**2+**^ was capable of dehydrogenating substrates with higher BDFE than ^**Me**^ **6** ^**2+**^ and ^**MeO**^ **6** ^**2+**^.

### BDFE Determination of the Fe-ECPB Systems: Comproportionation Reactions

As stated, the PCET reactions described above suggested that the BDFE_avg_ of the ^**X**^ **6** ^**2+**^ **/** ^**X**^ **8H**_**2**_^**2+**^ couples (BDFE_1,2_) were higher than BDFE_avg_ of the ^**X**^ **8H**_**2**_^**2+**^/^**X**^ **10H**_**4**_^**2+**^ couples (BDFE_avg1,2_ > BDFE_avg3,4_). When ^**X**^ **6** ^**2+**^ and ^**X**^ **12H**_**6**_^**2+**^ are mixed in solution, the formation of ^**X**^ **8H**_**2**_^**2+**^ and ^**X**^ **10H**_**4**_^**2+**^ is observed, a comproportionation reaction that can be described also as ligand-exchange reaction (i.e., the reaction between of ^**X**^ **6** ^**2+**^ and ^**X**^ **12H**_**2**_^**2+**^ to form ^**X**^ **8H**_**2**_^**2+**^ and ^**X**^ **10H**_**4**_^**2+**^ involves the exchange of a catechol-like and a quinone-like ligand).

The equilibria constants for the comproportionation reactions are related to the BDFE_avg_ of the ^**X**^ **6** ^**2+**^ **/** ^**X**^ **8H**_**2**_^**2+**^, ^**X**^ **8H**_**2**_^**2+**^ **/** ^**X**^ **10H**_**2**_^**2+**^ and ^**X**^ **10H**_**4**_^**2+**^ **/** ^**X**^ **12H**_**2**_^**2+**^ couples (see Scheme S1, see Table 2). The ΔG^0^ for the ligand-exchange reaction between ^**X**^ **6H**_**2**_^**2+**^ and ^**X**^ **12H**_**6**_^**2+**^(^**X**^ **6H**_**2**_^**2+**^ + ^**X**^ **12H**_**6**_^**2+**^ ↔ ^**X**^ **8H**_**2**_^**2+**^ + ^**X**^ **10H**_**4**_^**2+**^) is equal to the difference in BDFE_avg_ of the ^**X**^ **6H**_**2**_^**2+**^/ ^**X**^ **8H**_**4**_^**2+**^ and the ^**X**^ **10H**_**4**_^**2+**^/^**X**^ **12H**_**6**_^**2+**^ couples multiplied by 2 (see Scheme S1). The reaction between ^**Me**^ **6** ^**2+**^ and ^**Me**^ **12H**_**6**_^**2+**^ was followed by ^1^H NMR in CD_3_CN until the equilibrium was reached (∼10 days), leading to a mixture containing ^**Me**^ **6** ^**2+**^, ^**Me**^ **8H**_**2**_^**2+**^, ^**Me**^ **10H**_**6**_^**2+**^ and ^**Me**^ **12H**_**6**_^**2+**^ (see data in the Supporting Information). The equilibrium constant calculated for the comproportionation reaction indicated that the BDFE_avg_ of the ^**Me**^ **6** ^**2+**^ **/** ^**Me**^ **8H**_**2**_^**2+**^ couple was higher than the ^**Me**^ **10H**_**4**_^**2+**^ **/** ^**Me**^ **12H**_**6**_^**2+**^ couple (BDFE_avg1,2_ – BDFE_avg5,6_ ∼1 kcal/mol). Using the BDFE_avg_ value of the ^**Me**^ **6** ^**2+**^ **/** ^**Me**^ **8H**_**2**_^**2+**^ couple obtained by OCP measurements (BDFE_avg1,2_ = 64.4 kcal/mol), we can estimate the BDFE_avg_ of the ^**Me**^ **10H**_**4**_^**2+**^ **/** ^**Me**^ **12H**_**6**_^**2+**^ couple (BDFE_avg5,6_ = 63.6 kcal/mol). Based on the reactivity experiments described above, we determined that BDFE_avg1,2_ is higher than BDFE_avg3,4_. Taking into account that complex ^**Me**^ **8H**_**2**_^**2+**^ did not react with 2,6-Me_2_-H_2_Q (BDFE_avg_ = 64.6 kcal/mol) but did react with 2,6-(MeO)_2_-H_2_Q (BDFE_avg_ = 62.8 kcal/mol) allowed us to estimate that the BDFE_avg3,4_^**Me**^ **8H**_**2**_^**2+**^ **/** ^**Me**^ **10H**_**4**_^**2+**^ was approximately 63 kcal/mol. After that, we could also estimate the overall BDFE_avg_ for the ^**Me**^ **6** ^**2+**^ **/** ^**Me**^ **12H**_**6**_^**2+**^ couple (BDFE_avg1,6_ ∼ 63 kcal/mol).

**Table 2:** BDFE_avg_ Values Obtained by OCP Measurements, a Comproportionation Reactions and Reactivity Experiments (See Details in the Supporting Information)

^**X**^ **6** ^**2+**^ **/** ^**X**^ **12H**_**6**_^**2+**^	BDFE_avg1,2_	BDFE_avg3,4_	BDFE_avg5,6_	BDFE_avg1,6_
X: H	66.2	∼65	64.9	∼65
X: Me	64.4	∼63	63.6	∼63
X: MeO	62.8	∼62	62.4	∼62

The comproportionation reactions for the ^**H**^ **6** ^**2+**^ **/** ^**H**^ **12H**_**6**_^**2+**^ and ^**MeO**^ **6** ^**2+**^ **/** ^**MeO**^ **12H**_**6**_^**2+**^ systems were also performed (see details in the Supporting Information). Like in the ^**Me**^ **6** ^**2+**^ **/** ^**Me**^ **12H**_**6**_^**2+**^ system, the addition of equimolar amounts of ^**X**^ **6** ^**2+**^ **/** ^**X**^ **12H**_**6**_^**2+**^ led to the formation of ^**X**^ **8H**_**2**_^**2+**^ as the major species in solution and ^**X**^ **10H**_**4**_^**2+**^ as the minor. Following a similar reasoning to the one used for the ^**Me**^ **6** ^**2+**^ **/** ^**Me**^ **12H**_**6**_^**2+**^ system, we could determine the BDFE_avg_ of the ^**X**^ **10H**_**4**_^**2+**^ **/** ^**X**^ **12H**_**6**_^**2+**^ couples, and estimate the BDFE_avg_ of the ^**X**^ **8H**_**2**_^**2+**^ **/** ^**X**^ **10H**_**4**_^**2+**^ couples as well as the overall BDFE_avg1,6._ The BDFE values observed agreed with the reactivity of the ^**X**^ **6** ^**2+**^ complexes toward PCET donors described above (see Table 1), in which ^**H**^ **6** ^**2+**^ acted as a stronger H atom abstractor when compared to ^**Me**^ **6** ^**2+**^ and ^**MeO**^ **6** ^**2+**^.

The comproportionation reactions described above could also occur via a 1H^+^/1e^–^ PCET event between ^**X**^ **6** ^**2+**^ and ^**X**^ **12H**_**6**_^**2+**^ to produce ^**X**^ **7H** ^**2+**^ and ^**X**^ **11H**_**5**_^**2+**^, followed by a second intermolecular PCET step to generate ^**X**^ **8H**_**2**_^**2+**^ and ^**X**^ **10H**_**4**_^**2+**^ (as opposed to ligand-exchange reactions). To gather further insights into the mechanism, we compared four comproportionation reactions: ^**MeO**^ **6** ^**2+**^ + ^**H**^ **12H**_**6**_^**2+**^; ^**H**^ **6** ^**2+**^ + ^**MeO**^ **12H**_**6**_^**2+**^, ^**H**^ **6** ^**2+**^ + ^**H**^ **12H**_**6**_^**2+**^; and ^**MeO**^ **6** ^**2+**^ + ^**MeO**^ **12H**_**6**_^**2+**^ (see Supporting Information for details). The reaction between ^**H**^ **6** ^**2+**^ and ^**MeO**^ **12H**_**6**_^**2+**^ was extremely fast and produced ^**H**^ **8H**_**2**_^**2+**^ and ^**MeO**^ **10H**_**4**_^**2+**^, indicative of a net 2H^+^/2e^–^ PCET event. This reaction also generated other NMR peaks that resemble ^**X**^ **8H**_**2**_^**2+**^ and ^**X**^ **10H**_**4**_^**2+**^, and that were assigned as Fe complexes containing different opda ligands (e.g., ^**X**^ **8H**_**2**_^**2+**^-like species such as [Fe^II^(^q^L^H^)_2_(^cat^LH_2_^MeO^)]^2+^ or [Fe^II^(^q^L^H^)­(^q^L^MeO^)­(^cat^LH_2_^MeO^)]^2+^). Hence, these so-called comproportionation reactions between ^**X**^ **6** ^**2+**^ and ^**X**^ **12H**_**6**_^**2+**^ occur via PCET events (^**X**^ **6** ^**2+**^ + ^**X**^ **12H**_**6**_^**2+**^ → ^**X**^ **7H** ^**2+**^ + ^**X**^ **11H**_**5**_^**2+**^ → ^**X**^ **8H**_**2**_^**2+**^ + ^**X**^ **10H**_**4**_^**2+**^) but also involve the exchange of ^q^L and ^cat^LH_2_ ligands between two iron complexes (^**X**^ **6** ^**2+**^ + ^**X**^ **12H**_**6**_^**2+**^ → ^**X**^ **8H**_**2**_^**2+**^ + ^**X**^ **10H**_**4**_^**2+**^). The reaction between ^**MeO**^ **6** ^**2+**^ and ^**H**^ **12H**_**6**_^**2+**^ was very slow (only trace amounts of ^**MeO**^ **8H**_**2**_^**2+**^ were observed after 2 days), in contrast to the reaction between ^**H**^ **6** ^**2+**^ and ^**MeO**^ **12H**_**6**_^**2+**^ (full decay of ^**H**^ **6** ^**2+**^ was observed in minutes). The reactions between ^**H**^ **6** ^**2+**^ and ^**H**^ **12H**_**6**_^**2+**^ and between ^**MeO**^ **6** ^**2+**^ and ^**MeO**^ **12H**_**6**_^**2+**^ were not as fast as the reaction between ^**H**^ **6** ^**2+**^ and ^**MeO**^ **12H**_**6**_^**2+**^ (full decay of ^**X**^ **6** ^**2+**^ was observed in 2 days). These kinetic observations are in agreement with the thermochemistry of the transformations, being ^**MeO**^ **6** ^**2+**^ + ^**H**^ **12H**_**6**_^**2+**^ endergonic (^**MeO**^ **6** ^**2+**^ + ^**H**^ **12H**_**6**_^**2+**^ → ^**MeO**^ **8H**_**2**_^**2+**^ + ^**H**^ **10H**_**4**_^**2+**^; Δ*G* ^0^ = 4.2 kcal/mol), ^**MeO**^ **6** ^**2+**^ + ^**MeO**^ **12H**_**6**_^**2+**^ mildly exergonic (^**MeO**^ **6** ^**2+**^ + ^**MeO**^ **12H**_**6**_^**2+**^ → ^**MeO**^ **8H**_**2**_^**2+**^ + ^**MeO**^ **10H**_**4**_^**2+**^; Δ*G* ^0^ = −0.8 kcal/mol), ^**H**^ **6** ^**2+**^ + ^**H**^ **12H**_**6**_^**2+**^ exergonic (^**H**^ **6** ^**2+**^ + ^**H**^ **12H**_**6**_^**2+**^ → ^**H**^ **8H**_**2**_^**2+**^ + ^**H**^ **10H**_**4**_^**2+**^; Δ*G* ^0^ = −2.6 kcal/mol), and ^**H**^ **6** ^**2+**^ + ^**MeO**^ **12H**_**6**_^**2+**^ highly exergonic (^**H**^ **6** ^**2+**^ + ^**MeO**^ **12H**_**6**_^**2+**^ → ^**H**^ **8H**_**2**_^**2+**^ + ^**MeO**^ **10H**_**4**_^**2+**^; Δ*G* ^0^ = −7.6 kcal/mol).

### Cyclic Voltammetry: Thermochemical Decompensation

Cyclic voltammetry (CV) measurements for ^**H**^ **6** ^**2+**^, ^**Me**^ **6^2+^,** and ^**MeO**^ **6** ^**2+**^ were carried out in CH_3_CN (see Supporting Information for details). For all three complexes, we observed two redox events, corresponding to the 1e^–^ reduction of ^**X**^ **6** ^**2+**^ (*E*_1/2_ between −0.54 and −0.79 V vs Fc^0/+^) and 1e^–^ oxidation ^**X**^ **6** ^**2+**^ (*E*_1/2_ between 0.88 and 0.62 V vs Fc^0/+^. Note: the 1e^–^ oxidation of ^**MeO**^ **6** ^**2**+^ was irreversible, see Supporting Information).^23^ The CV of ^**X**^ **8H**_**2**_^**2+**^ and ^**X**^ **12H**_**6**_^**2+**^ led to irreversible redox processes. As expected, the reduction of potentials for the 1e^–^ reduction and 1e^–^ oxidation of ^**X**^ **6** ^**2+**^ shifted to more negative values for the Fe complexes containing electron-donating substituents in the opda ligand (H > Me > MeO).

For the Cu-based ECPBs, we have recently found that the BDFE_avg_ for the 4H^+^/4e^–^ reductive protonation of ^**X,R**^ **5** ^**+**^ to ^**X,R**^ **1H**_**4**_^**+**^ (see Figure 2) increased proportionally to the *E*_1/2_ of the 1e^–^ reduction of ^**X,R**^ **5** ^**+**^ (see Figure 9). This effect, referred as thermochemical decompensation, is consistent with the BDFEs being more affected by changes in reduction potentials than changes in the p*K*a’s, and it has been observed in organic molecules (such as phenols or anilines) and metal complexes (such as Cu^III^OH intermediates).^29^ For the Fe-based ECPBs described herein, a similar trend was observed.

**Figure 9:**
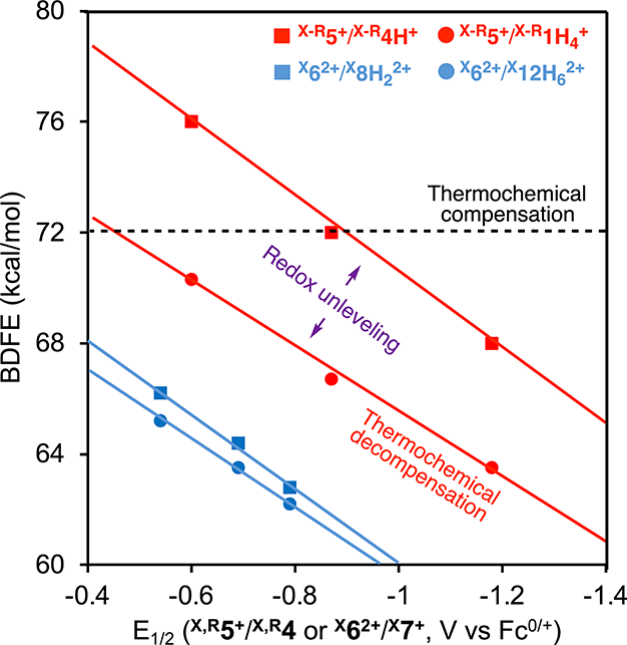
Thermochemical decompensation and redox unleveling effects observed in the Cu-based ECPBs (in red) and Fe-based ECPBs (in blue). Note: redox unleveling for the Cu-based ECPBs appears to be more pronounced, but it should be noted that we are comparing redox couples with different number of protons and electrons (1H^+^/1e^–^ vs 4H^+^/4e^–^ in Cu; 2H^+^/2e^–^ vs 6H^+^/6e^–^ in Fe).

### Buffering Capabilities of the Fe-Based ECPB System

To support that the Fe ECPB system could be used as a buffer for protons and electrons, we reacted equimolar mixtures of ^**Me**^ **6** ^**2+**^ and ^**Me**^ **12H**_**6**_^**2+**^ with PCET donors (e.g., hydroquinones) and PCET acceptors (e.g., quinones) in a substoichiometric fashion. Equimolar mixtures of ^**Me**^ **6** ^**2+**^ and ^**Me**^ **12H**_**6**_^**2+**^ were reacted until the equilibrium between ^**Me**^ **6** ^**2+**^, ^**Me**^ **8H**_**2**_^**2+**^, ^**Me**^ **10H**_**4**_ **^2+^,** and ^**Me**^ **12H**_**6**_^**2+**^ was reached and then 2,6-(MeO)_2_-H_2_Q was added (see Figure 10A). The reaction of the buffer with hydroquinone led to the immediate formation of the quinone product, shifting the equilibria to the formation of ^**Me**^ **10H**_**2**_^**2+**^ and ^**Me**^ **12H**_**6**_^**2+**^. After several hours, a new equilibrium was reached in which ^**Me**^ **8H**_**2**_^**2+**^, ^**Me**^ **10H**_**4**_^**2+**^, ^**Me**^ **12H**_**6**_^**2+**^, 2,6-(MeO)_2_-H_2_Q and 2,6-(MeO)_2_-Q were observed. As expected, the equivalents of quinone product formed (from 0 to 3.3 mM) agreed with the increase of the overall hydroquinone-like ligand concentration (from 28.9 to 31.1 mM).

**Figure 10:**
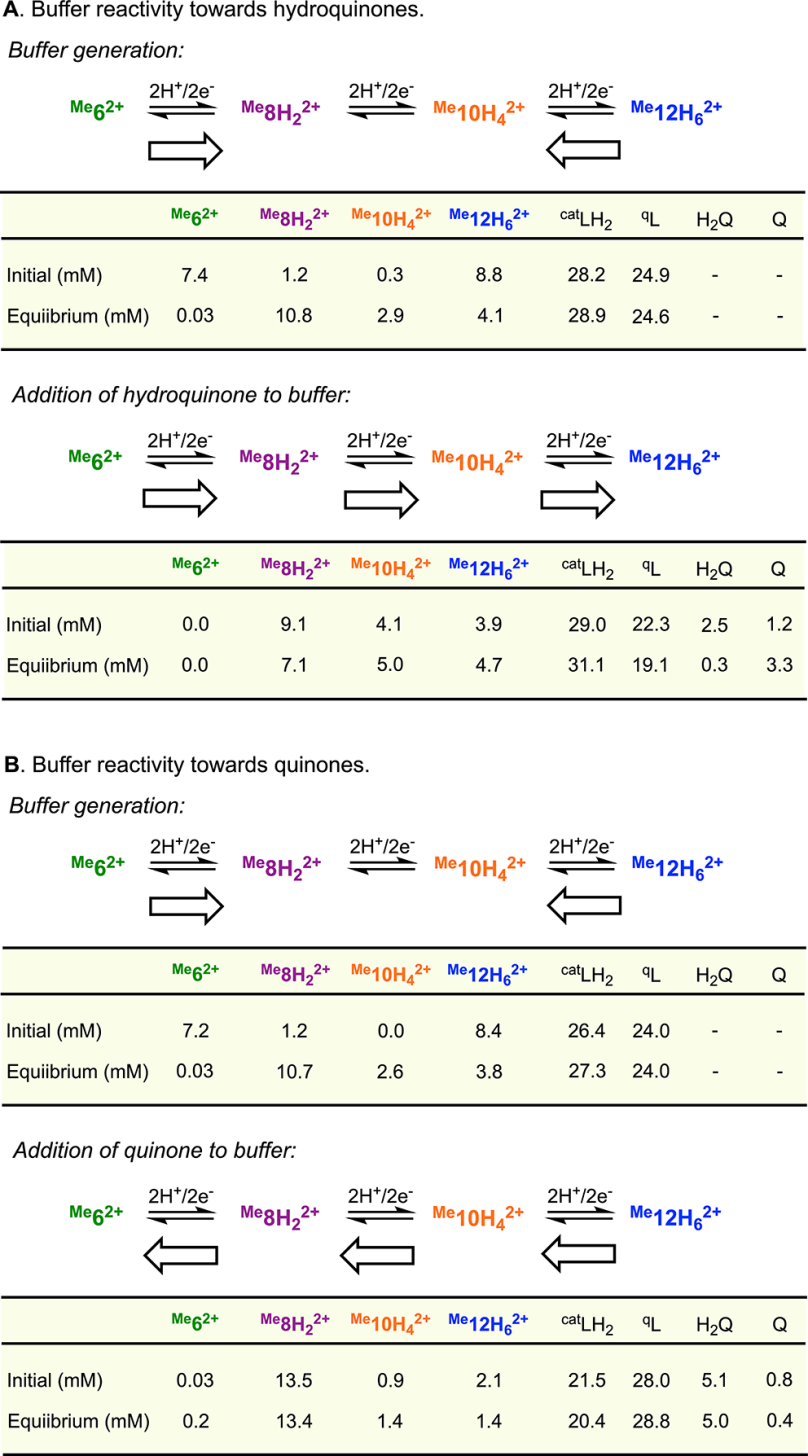
Buffering reactivity of the ^**Me**^ **6** ^**2+**^/^**Me**^ **12H**_**6**_^**2+**^ couple using 2,6-(MeO)_2_-H_2_Q as PCET donor (A) and 1,4-Q as PCET acceptor (B). Note: ^cat^LH_2_ and ^q^LH denote the ligands of the Fe complexes in the fully protonated-reduced form (^cat^LH_2_) or fully deprotonated-oxidized form (^q^L). The data included in first and the third table is from two different comproportionation experiments. Note that “initial” indicate the ^1^H NMR measurement carried out after addition of reagents (∼5 min).

A similar buffer containing ^**Me**^ **6** ^**2+**^, ^**Me**^ **8H**_**2**_^**2+**^, ^**Me**^ **10H**_**4**_ **^2+^,** and ^**Me**^ **12H**_**6**_^**2+**^ was reacted with 1,4-quinone (see Figure 10B). The addition of the quinone led to immediate 1,4-hydroquinone, shifting the equilibria toward the formation of ^**Me**^ **6** ^**2+**^ and ^**Me**^ **8H**_**2**_^**2+**^. Interestingly, the 1,4-hydroquinone/quinone ratio did not change substantially overtime, but the relative concentrations of ^**Me**^ **6** ^**2+**^, ^**Me**^ **8H**_**2**_^**2+**^, ^**Me**^ **10H**_**4**_ **^2+^,** and ^**Me**^ **12H**_**6**_^**2+**^ did. As expected, the formation of the 1,4-hydroquinone product (from 0 to 5 mM) agreed with the increase of overall quinone-like ligand concentration (from 24 to 28 mM).

### Decoupled Substrate Dehydrogenation using O_2_ as Oxidant

By definition, ECPB systems capture and deliver H atom equivalents in a decoupled and reversible fashion. In some of our recent publications, we have shown that Cu-ECPB systems can be used in the decoupled dehydrogenation of organic molecules (e.g., hydroquinones and PhNHNHPh) using O_2_ as oxidant. To demonstrate that the Fe-ECBP systems were capable of performing such transformations, we used the ^**MeO**^ **6** ^**2+**^ **/** ^**MeO**^ **12H**_**6**_^**2+**^ system to decouple the oxidative deprotonation of PhNHNHPh with the reductive protonation of O_2_ (Figure 11). During the first cycle, we reacted ^**MeO**^ **6** ^**2+**^ with 3 equiv of PhNHNHPh under anaerobic conditions. The reaction was followed by NMR and we observed the formation of ^**MeO**^ **12H**_**6**_^**2+**^ and PhN=NPh (∼75% for ^**MeO**^ **12H**_**6**_^**2+**^, 71% for PhN=NPh). After full formation of ^**MeO**^ **12H**_**6**_^**2+**^, we added O_2_ to the solution to produce ^**MeO**^ **6** ^**2+**^. The third cycle was then carried out by removing the excess of O_2_ and adding 3 equiv of PhNHNHPh into the CD_3_CN solution containing complex ^**MeO**^ **6** ^**2+**^. Regeneration of ^**MeO**^ **12H**_**6**_^**2+**^ and formation of PhN=NPh was monitored until completion, which prompted us to add O_2_ to initiate the fourth cycle. Overall, we observed a substantial decay of ECPB mass balance in each of the cycles (down to 40% after four cycles).

**Figure 11:**
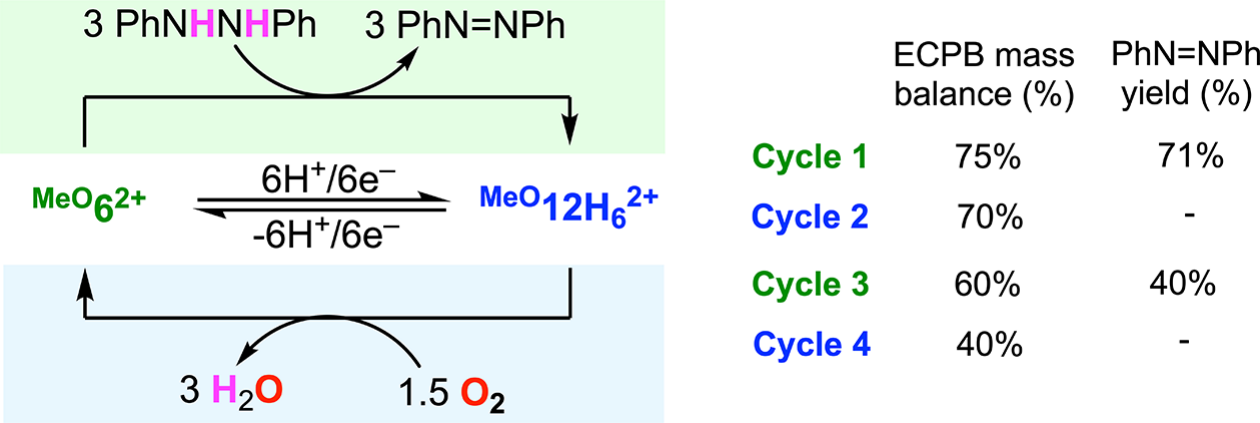
Decoupled dehydrogenation of PhNHNHPh with O_2_ using Fe-based ECPBs. See Supporting Information for experimental details.

### Redox “Unleveling” in Cu-Based and Fe-Based ECPBs

To visualize redox (un)­leveling in species capable of accepting more than one H atom equivalent (e.g., 4H^+^/4e^–^ reduction of O_2_ to H_2_O), one can plot the stepwise thermochemical changes of an isoergonic transformation involving the species studied (e.g., O_2_) and a fictitious 1H^+^/1e^–^ PCET reagent (X-H) with a BDFE equal to the BDFE_avg_ of the couple studied (e.g., BDFE_(X‑H)_ = BDFE_avg_(O_2_/H_2_O) = 81.1 kcal/mol, see Figure 12). These plots, which resemble the Frost diagrams, allow to determine the thermodynamic tendency of the intermediate species formed during PCET transformations to accept/donate H atom equivalents and to undergo disproportionation reactions. For example, the thermodynamic driving force for the 1H^+^/1e^–^ reduction of dioxygen to hydroperoxyl radical (HO_2_·), which is the BDFE of the O–H of formed, is very low when compared to the BDFE_avg_ of the 4H^+^/4e^–^ reduction of dioxygen to water (51.2 vs 81.2 kcal/mol), which makes O_2_ a very inefficient H atom abstractor despite being considered a strong oxidant (see Figure 12). In an isoergonic PCET reaction, this 1H^+^/1e^–^ reduction is highly endergonic (ΔG^0^_1_ ≫ 0), a situation that usually leads to slow PCET transformations (i.e., in general, the lower is the driving force for a PCET reaction, the slower this will be). Once generated, the hydroperoxyl radical can react with the PCET reagent in an exergonic fashion (ΔG^0^_1_ < 0) since the BDFE associated with the formation of the O–H bond of H_2_O_2_ is slightly higher than the BDFE_avg_ of the O_2_/H_2_O couple (86.5 kcal/mol vs 81.2 kcal/mol). Alternatively, the hydroperoxyl radical can also undergo disproportionation reactions to generate O_2_, H_2_O_2_, which can ultimately produce H_2_O. In an isoergonic reaction, the thermochemistry of these disproportionation reactions (formally 1H^+^/1e^–^ and 2H^+^/2e^–^ events) can also be determined if the stepwise BDFEs are known. For the O_2_/H_2_O couple, the 1H^+^/1e^–^ disproportionation reactions are thermodynamically favored due to the high difference between the BDFE of the O_2_/HO_2_· couple (BDFE_1_ = 51.2 kcal/mol) and the BDFE of the HO·/H_2_O couple (BDFE_1_ = 115.8 kcal/mol). Additionally, the 2H^+^/2e^–^ disproportionation of H_2_O_2_ to produce O_2_ and H_2_O is also thermodynamically favored due to the high difference between the BDFE_avg_ of the O_2_/H_2_O_2_ couple (BDFE_1,2_ = 69 kcal/mol) and the BDFE_avg_ of the H_2_O_2_/H_2_O couple (BDFE_3,4_ = 93.5 kcal/mol).

**Figure 12:**
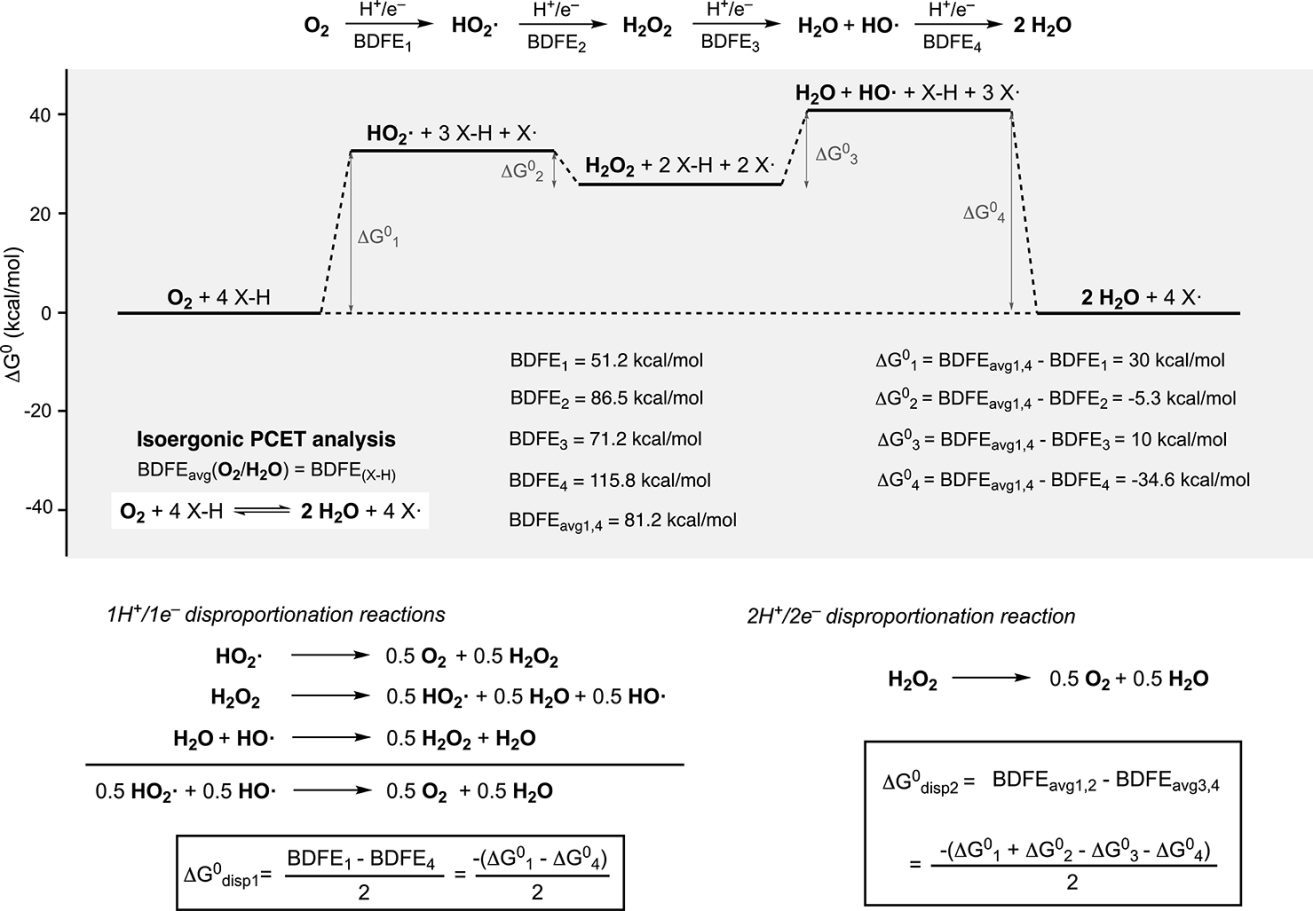
Isoergonic analysis of the redox unleveling observed in the 4H^+^/4e^–^ reduction of dioxygen to water.

In one of our recent publications, we included the isoergonic PCET analysis of the Cu-based ECPB system **5** ^**+**^/**1H**_**4**_^**+**^ (see Figure 13). The average BDFE of the **5** ^**+**^/**1H**_**4**_^**+**^ couple (BDFE_1,4_ = 70.3 kcal/mol) and the stepwise BDFE of the **5** ^**+**^/**4H** ^**+**^ and **2H**_**3**_^**+**^/**1H**_**4**_^**+**^ coupled (BDFE_1_ ∼ 76 kcal/mol; and BDFE_4_ ∼ 80 kcal/mol) were experimentally determined. Like in the isoergonic analysis of the O_2_/H_2_O couple, the thermodynamics of the 1H^+^/1e^–^ disproportionation reactions only depended on BDFE_1_ and BDFE_4_, and that for the **5** ^**+**^/**1H**_**4**_^**+**^ system was exergonic, which agreed with the fast disproportionation kinetics observed. We also measured the kinetics for the ligand-exchange reactions between **5** ^**+**^ and **1H**_**4**_^**+**^ (formal 2H^+^/2e^–^ disproportionation of **3H**_**2**_^**+**^to **5** ^**+**^ and **1H**_**4**_^**+**^), which were fast and did not involve the formation of **3H**_**2**_^**+**^, allowing us to hypothesize that the average BDFE of the **5** ^**+**^/**3H**_**2**_^**+**^ couple (BDFE_1,2_) was lower than the BDFE of the **3H**_**2**_^**+**^/**1H**_**4**_^**+**^ couple (BDFE_3,4_). The kinetics of an isoergonic PCET reaction involving **5** ^**+**^/**1H**_**4**_^**+**^ and a PCET reagent were also measured and were found to be 3 orders of magnitude slower than the ones observed for the ligand-exchange reaction. Overall, it was proposed that the 4H^+^/4e^–^ reactivity of the Cu-based ECPBs was dictated by the slow PCET reactions followed by fast disproportionation (formally 1H^+^/1e^–^ events) and fast ligand-exchange reactions (formally 2H^+^/2e^–^ events) to maintain the ^**X,R**^ **5** ^**+**^ and ^**X,R**^ **1H**_**4**_^**+**^ equilibria.

**Figure 13:**
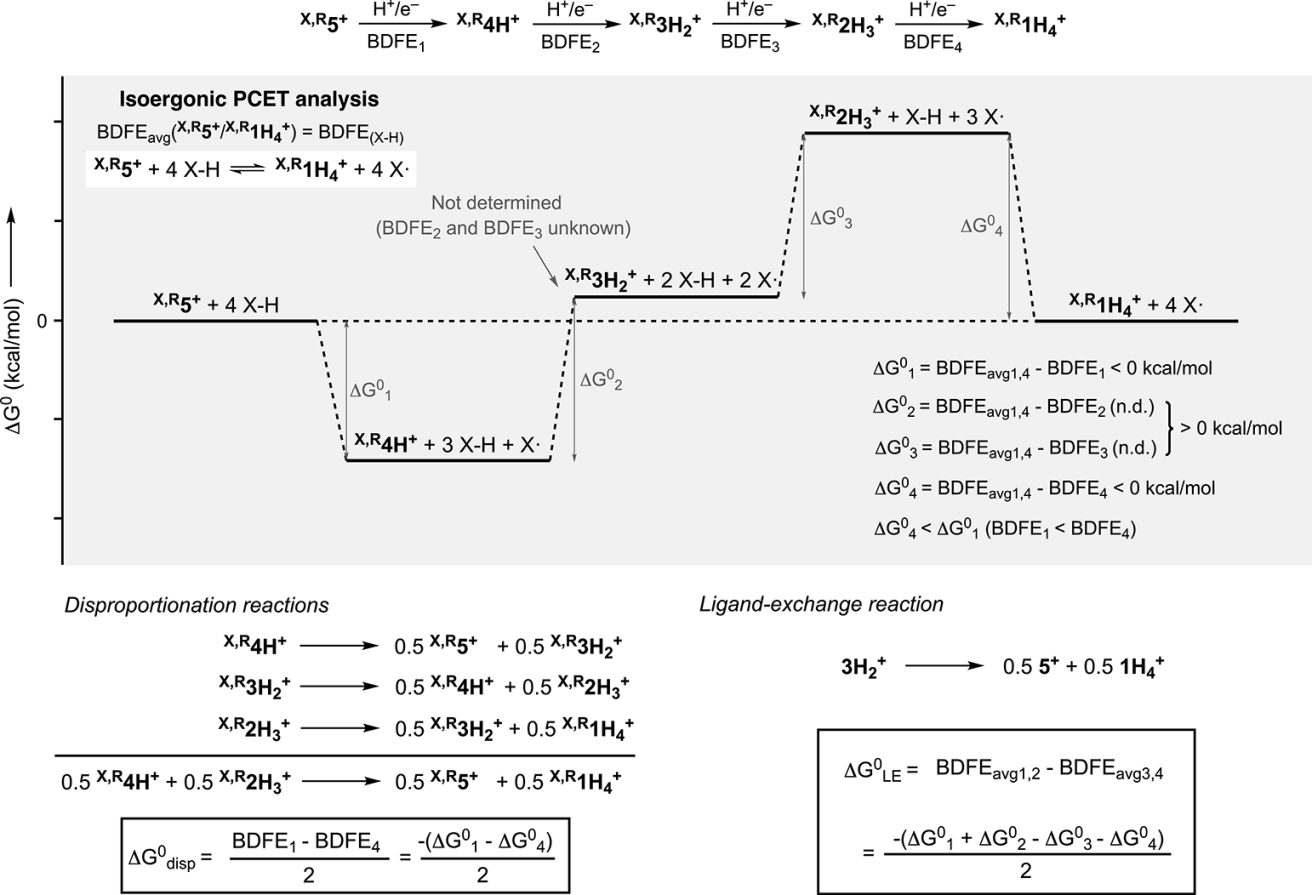
Isoergonic analysis of the redox unleveling observed in the 4H^+^/4e^–^ reduction of ^**X,R**^ **5** ^**+**^ to ^**X,R**^ **1H**_**4**_^**+**^.

For the Fe-ECPBs described herein, an isoergonic PCET analysis can also be carried out (see Figure 14). When compared to the Cu-ECPBs, none of the stepwise BDFEs could be determined, but we have obtained the average BDFE of the ^**X**^ **6** ^**2+**^ **/** ^**X**^ **8H**_**2**_^**2+**^, ^**X**^ **8H**_**2**_^**2+**^ **/** ^**X**^ **10H**_**4**_ **^2+^,** and ^**X**^ **10H**_**2**_^**2+**^ **/** ^**X**^ **12H**_**6**_^**2+**^ couples. The fact that during the PCET reactions (e.g., reaction of ^**Me**^ **6** ^**2+**^ with H_2_Q to produce ^**Me**^ **8H**_**2**_^**2+**^) and the comproportionation reactions (e.g., reaction between ^**Me**^ **6** ^**2+**^ and ^**Me**^ **12H**_**6**_^**2+**^ to produce mixtures of ^**Me**^ **6** ^**2+**^, ^**Me**^ **8H**_**2**_^**2+**^, ^**Me**^ **10H**_**4**_^**2+**^, and ^**Me**^ **12H**_**6**_^**2+**^) no Fe species other than ^**X**^ **6** ^**2+**^, ^**X**^ **8H**_**2**_^**2+**^, ^**X**^ **10H**_**4**_^**2+**^, and ^**X**^ **12H**_**6**_^**2+**^ could be detected implies that the stepwise BDFE for the formation of the intermediate Fe-semiquinone species ^**X**^ **7H** ^**2+**^, ^**X**^ **9H**_**3**_^**2+**^, and ^**X**^ **11H**_**5**_^**2+**^ are lower than the stepwise BDFE for the formation of ^**X**^ **8H**_**2**_^**2+**^, ^**X**^ **10H**_**4**_^**2+**^, and ^**X**^ **12H**_**6**_^**2+**^ (i.e., BDFE_1_ < BDFE_2_, BDFE_3_ < BDFE_4_ and BDFE_5_< BDFE_6_, see Figure 14). Based on this assumption (BDFE_avg_ > BDFE_n_ and BDFE_avg_ < BDFE_n+1_ for n: 1,3 and 5), the 1H^+^/1e^–^ disproportionation reactions involved in the Fe-ECPB reactivity should be thermodynamically favored (BDFE_1_ < BDFE_6_). Conversely, the thermodynamic analysis of the ligand-exchange reactions involved in the Fe-ECPB systems suggests that these processes are overall endergonic (ΔG^0^_LE_ = BDFE_avg1,2_ – BDFE_avg3,4_, with BDFE_avg1,2_ > BDFE_avg5,6_ leading to ΔG^0^_LE_ > 0). Our thermochemical analysis indicates that the 6H^+^/6e^–^ ECPB reactivity of the and ^**X**^ **6** ^**2+**^ **/** ^**X**^ **12H**_**6**_^**2+**^ couples is dictated by the slow PCET reactions (e.g., ^**X**^ **6** ^**2+**^ + H· → ^**X**^ **7H** ^**2+**^, slow) followed by fast disproportionation (e.g., ^**X**^ **7H** ^**2+**^ → 0.5 ^**X**^ **6** ^**2+**^ + 0.5 ^**X**^ **8H**_**2**_^**2+**^, fast) and slow ligand-exchange reactions (e.g., ^**X**^ **8H**_**2**_^**2+**^ → 0.5 ^**X**^ **6** ^**2+**^ + 0.5 ^**X**^ **10H**_**4**_^**2+**^, slow). This allows to observe the formation of ^**X**^ **8H**_**2**_^**2+**^ and ^**X**^ **10H**_**4**_^**2+**^ during the reductive protonation of ^**X**^ **6** ^**2+**^ to ^**X**^ **12H**_**6**_^**2+**^ and the oxidative deprotonation of ^**X**^ **6** ^**2+**^ to ^**X**^ **12H**_**6**_^**2+**^, which leads to a stepwise ECPB system.

**Figure 14:**
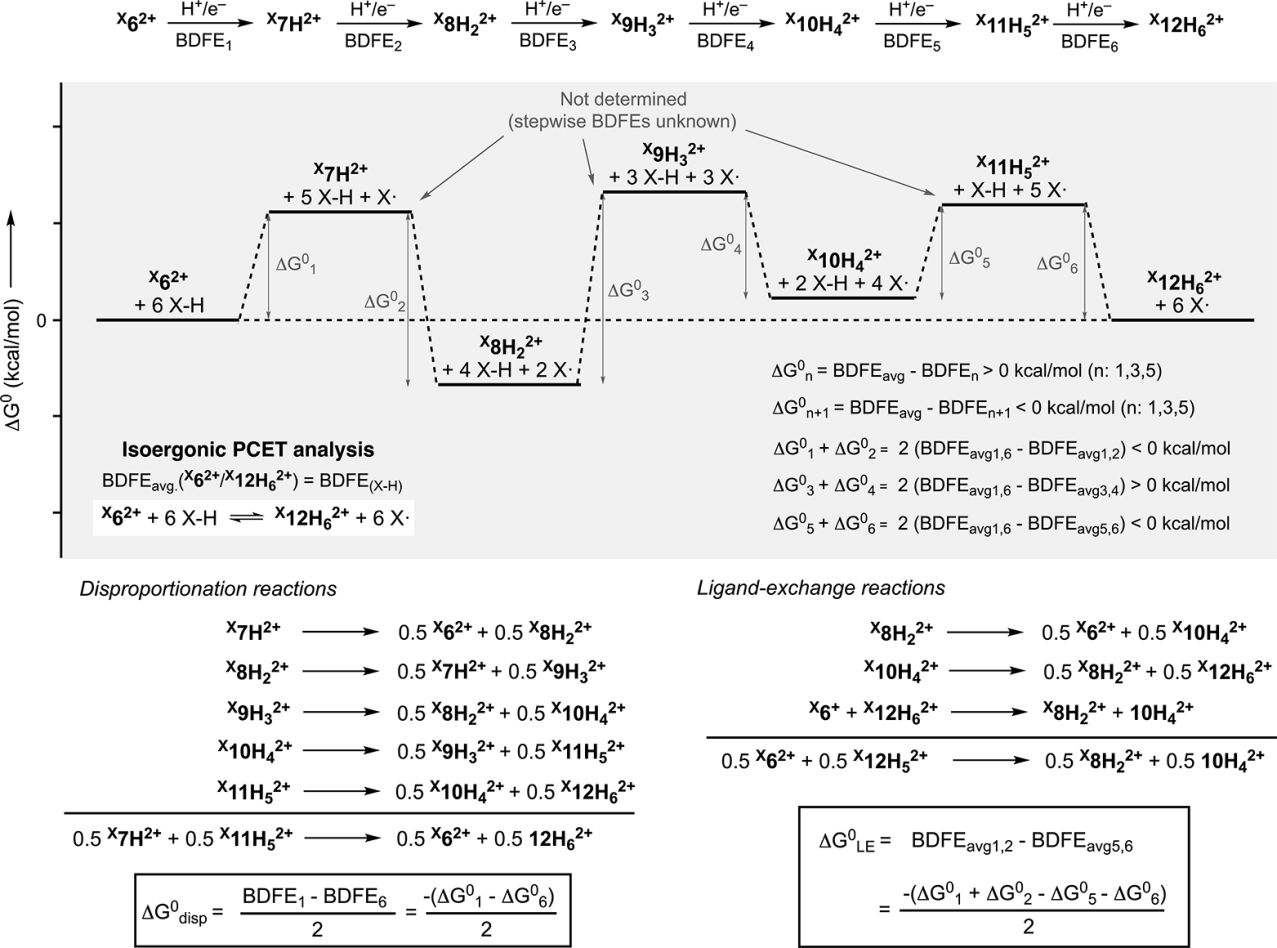
Isoergonic analysis of the redox unleveling observed in the 6H^+^/6e^–^ reduction of ^**X**^ **6** ^**2+**^ to ^**X**^ **12H**_**6**_^**2+**^.

During the execution of this research project, we noticed that the PCET transformations involving Fe-ECPB systems were generally much slower than the ones involving the Cu analogues. To corroborate this perception, we analyzed the kinetics of the reductive protonation of ^**Me**^ **6** ^**2+**^ and ^**Me,H**^ **5** ^**2+**^ (Fe^II^ and Cu^I^ complexes bearing the same quinone-like ligand) using PhNHNHPh as PCET donor. The reactions were carried out at room temperature in two different solvents (DMF and CH_3_CN) under pseudo-first order conditions ([ECPB] = 2 mM; [PhNHNHPh] = 20 mM, see Supporting Information). The reactions between ^**Me,H**^ **5** ^**2+**^ and PhNHNHPh, both in DMF and CH_3_CN, finalized in less than 30 min. Conversely, the conversion of ^**Me**^ **6** ^**2+**^ to ^**Me**^ **12H**_**6**_^**2+**^ using PhNHNHPh under the same conditions took more than a week. Despite being preliminary (a more thorough kinetic analysis will be carried out in the future), these findings can be explained by the fact that (i) the thermodynamic driving force for the overall ^**Me,H**^ **5** ^**+**^ reaction (^**Me,H**^ **5** ^**+**^ + 2 PhNHNHPh → ^**Me,H**^ **1H**_**5**_^**+**^ + 2 PhN=NPh, ΔG^0^ = −23.2 kcal/mol) is slightly more exergonic than the ^**Me**^ **6** ^**2+**^ reaction (^**Me**^ **6** ^**2+**^ + 3 PhNHNHPh → ^**Me**^ **12H**_**6**_^**2+**^ + 3 PhN=NPh, ΔG^0^ = −15.6 kcal/mol); (ii) the first H atom transfer from the substrate to the oxidant is more thermodynamically favored for the Cu system (BDFE_1_ < BDFE_avg1,6_ for ^**Me**^ **6** ^**2+**^, BDFE_1_ > BDFE_avg1,4_ for ^**Me,H**^ **5** ^**+**^), and (iii) the disproportionation and ligand-exchange reactions of the Cu systems are both thermodynamically favored, which lead to fast regeneration of ^**Me,H**^ **5** ^**+**^.

## Conclusions

In this research article, we reported the first example of Fe-based ECPB system, capable of performing reversible 6H^+^/6e^–^ transformations in a stepwise fashion. Our analysis indicated that the Fe-based ECPBs depicted redox unleveling, in which the BDFE_avg_ of the ^**X**^ **6** ^**2+**^/^**X**^ **8H**_**2**_^**2+**^ couples was substantially higher than the BDFE_avg_ of the 6H^+^/6e^–^ conversion of ^**X**^ **6** ^**2+**^ to ^**X**^ **12H**_**6**_^**2+**^. We also showed that the PCET reactivity of the Fe ECPBs could also be tuned by using different redox-active ligands, with the systems containing electron-rich substituents (4,5-Me_2_-opda and 4,5-(MeO)_2_-opda) depicting lower BDFE_avg_ values lower than the H analogue (opda), a manifestation of redox decompensation. When compared to the Cu-based ECPBs, we found that the PCET reactivity of the Fe systems was slower, which allowed to observe the reaction intermediates ^**X**^ **8H**_**2**_^**2+**^ and ^**X**^ **10H**_**4**_^**2+**^. We hypothesize that the differences in the reactivity arise from the slow ligand-exchange rates observed in the Fe-ECPB systems (involving the inert low-spin octahedral 3d^6^ Fe^II^ complexes ^**X**^ **6** ^**2+**^, ^**X**^ **8H**_**2**_^**2+**^, and ^**X**^ **10H**_**4**_^**2+**^), in contrast with the fast ligand-exchange reactions observed in the Cu-analogues (which involve labile 3d^10^ Cu^I^ complexes).^30,31^

## Experimental Section

### Materials

All reagents were purchased from commercial suppliers and used as received except as noted. All solvents were purchased at the highest level of purity and further purified and dried by passing through an activated alumina solvent purification system (MB SPS-7, M. BRAUN INERTGAS-SYSTEME, Garching, Germany). Dimethylformamide (DMF) was distilled under partial vacuum before use. Deuterated solvents were purchased from Cambridge Isotope Laboratories (Tewksbury, MA, USA) and used as received. **Caution!** Metal perchlorate salts are strong oxidizing agents and can be explosive in the presence of open flames, heat, or sparks.

### Physical Methods

Air-free handling of the iron complexes was performed inside a MBRAUN UNIlab Pro SP glovebox system with N_2_ working gas. Electrochemical measurements were performed using a CH Instruments 620E Electrochemical Workstation (CH Instruments, Austin, TX, USA). UV–vis spectra were collected using a Hewlett-Packard 8454 diode array spectrophotometer with a 1 cm path quartz cell. The spectrometer was equipped with Agilent UV–visible ChemStation software (ver. B.05.02 [16], Agilent Technologies, Santa Clara, CA, USA) and a Unisoku CoolSpeK UV cryostat (UNISOKU Co., Hirakata, Japan). NMR spectra were on a 500 MHz NMR spectrometer (NEO 500 or Avance III, Bruker Corp., Billerica, MA) to acquire spectra with 16 cumulative scans. Evans method experiments were performed using a 7 in, 5 mm o.d. NMR tube with a smaller 3 mm o.d. NMR tube inserted inside. The outer tube contained the analyte dissolved in a deuterated solvent with a dichloromethane (DCM) internal standard. The smaller inner tube contained the same deuterated solvent and DCM internal standard solution (without analyte). XCHN analysis was performed by Midwest Micro Lab (Indianapolis, IN, USA). A PerkinElmer Frontier FT–IR Spectrometer with an attenuated total reflectance attachment containing a germanium crystal was used. Spectra were obtained over a range of 4000–700 cm^–1^ with 0.4 cm^–1^ resolution. SC-XRD measurements: Reflection intensities were measured at 110 K using either a SuperNova diffractometer (with Atlas detector) with Mo Kα radiation (λ = 0.71073 Å) or Cu Kα radiation (λ = 1.54178 Å) or a Rigaku XtaLAB Synergy R diffractometer (with a rotating-anode X-ray source and HyPix-6000HE detector) with Cu Kα radiation (λ = 1.54178 Å). Data collection, refinement of cell dimensions, and data reduction were performed using the program CrysAlisPro (see Supporting Information for more details). ^57^Fe Mössbauer spectra were collected on powder samples using two spectrometers employing Janis Research (Wilmington, MA) SuperVaritemp dewars equipped with a LakeShore Model 331 A temperature controller. The external magnetic field of 0.045 T was provided by a permanent magnet, while the external fields of 0.1 and 7 T were provided by a superconducting magnet. Powder samples were prepared by grinding a mixture of crystals and boron nitride (BN), and transferring the resulting powders into sample holders manufactured by polyoxymethylene. A threaded lid was used to apply compression and fix the powders. To achieve effective seal and protect the samples from air, vacuum grease was applied to the threaded lid. The data was simulated using quadrupole doublet model with Lorentzian line shapes. The asymmetric parameter is defined as η = (V_*xx*_–V_*yy*_)/V_*zz*_, where V_*xx*_, V_*yy*_, and V_*zz*_ are the three principal values of the electron field gradient tensor. Isomer shift values are referenced against that of α-Fe foil at 298 K.

### Synthesis and Characterization of the Iron Complexes

4,5-Dimethoxy-1,2-phenylenediamine and 4,5-dimethyl-1,2-phenylenediamine were synthesized following a reported procedure.^20^ Synthesis of ^**H**^ **6** ^**2+**^, ^**H**^ **8H**_**2**_^**2+**^, ^**H**^ **10H**_**4**_^**2+**^, and ^**H**^ **12H**_**6**_^**2+**^ were modified from reported methods.^22,23^ The synthesis and characterization of the MeO and H analogues was carried out following a protocol similar to the Me-substituted complexes described below. The Fe complexes were characterized by^1^H NMR, elemental analysis, SC-XRD, ^1^H NMR Evans method, UV–vis and Mössbauer spectroscopy (see further details in the Supporting Information).

^**H**^ **6** ^**2+**^: In a N_2_ filled glovebox, 4,5-dimethyl-1,2-phenylenediamine (408 mg, 3 mmol) was charged in a 30 mL vial and dissolved in 12 mL CH_3_CN, then [Fe­(H_2_O)_6_]­(ClO_4_)_2_ (360 mg, 1 mmol) was added as solid. Then the pale brown solution was transferred out of the glovebox and reacted with oxygen with an O_2_ balloon fitted on top of the vial in the presence of 4A molecular sieves. The color of the solution rapidly turned navy blue in 15 s, then slowly turned purple in 1 h. The reaction takes about 2–3 days for completion. It was monitored by NMR and stopped when there was only ^Me^6^2+^ in the solution. This could also be monitored visually – a bright green solution is indicative of the end point. The solution was filtered to get rid of molecular sieves and the solvent removed under vacuum. The crude product was crystallized by layering hexane on top of concentrated THF solution of **^Me^6^2+^** to afford crystal suitable for SC-XRD characterization (80% yield). Vapor diffusion of Et_2_O into a concentrated solution of **^Me^6^2+^** in CH_3_CN also yielded good crystals. ^1^H NMR (CD_3_CN): 2.21 (s, 18H, C**H**_3_), 6.80 (s, 6H, Ph-**H**), 11.27 (s, 6H, N**H**). Elemental analysis: Chemical Formula: (C_24_H_30_FeN_6_Cl_2_O_8_ x H_2_O). Calc: C (42.69%); H (4.78%); N (12.45%). Exp: C (42.69%); H (4.59%); N (12.13%). This complex was also characterized by SC-XRD (see Supporting Information).

^**Me**^ **8H**_**2**_^**2+**^: In a N_2_ filled glovebox, 4,5-dimethyl-1,2-phenylenediamine (408 mg, 3 mmol) was charged in a 30 mL vial and dissolved in 12 mL CH_3_CN, then [Fe­(H_2_O)_6_]­(ClO_4_)_2_ (360 mg, 1 mmol) was added as solid. Then the pale brown solution was transferred out the glovebox, reacting with oxygen in air. The color of the solution rapidly turned navy blue in 10 s, then slowly turned purple in 1 h. During the reaction, NMR was taken to monitor the formation of **^Me^8H_2_^2+^**. Upon completion, the reaction was quenched by removal of the solvent under vacuum to obtain the crude product (usually the crude contains ∼ 5% ^Me^6^2+^ due to overoxidation). In a N_2_-filled glovebox, the crude product was crystallized by layering hexane on top of a THF solution to afford dark crystals suitable for SC-XRD characterization (40% yield).^1^H NMR (CD_3_CN): 2.16 (s, 6H, C**H**_3_), 2.21 (s, 6H, C**H**_3_), 2.25 (s, 6H, C**H**_3_), 4.14 (d, 2H, N**H**_2_), 5.14 (d, 2H, N**H**_2_), 6.74 (s, 2H, Ph-**H**), 7.04 (s, 2H, Ph-**H**), 7.16 (s, 2H, Ph-**H**), 10.44 (s, 2H, N**H**), 12.20 (s, 2H, N**H**). Elemental analysis: Chemical Formula: (C_24_H_32_FeN_6_Cl_2_O_8_ x 2THF x 0.5 hexane). Calc: C (49.66%); H (6.55%); N (9.93%). Exp: C (49.18%); H (6.27%); N (9.96%). This complex was also characterized by SC-XRD (see Supporting Information).

^**Me**^ **10H**_**4**_^**2+**^: In a N_2_ filled glovebox, 4,5-methyl-1,2-phenylenediamine (205 mg, 1.5 mmol) was charged in a 30 mL vial and dissolved in 12 mL CH_3_CN, then [Fe­(H_2_O)_6_]­(ClO_4_)_2_ (180 mg, 0.5 mmol) was added as solid. Then the pale brown solution was transferred out the glovebox, reacting with 5.6 mL oxygen stored in a gastight syringe. The reaction was stopped 5 min after full consumption of O_2_. After removal of solvent in vacuum, the crude solid was transferred into glovebox. The resulting solid was analyzed by NMR and UV–vis spectroscopy (see Supporting Information). ^1^H NMR (CD_3_CN-*d*_*3*_): 2.19 (s, 18H, C**H**_3_), 6.78 (s, 6H, Ph-**H**), 11.26 (s, 6H, N**H**).

^**Me**^ **12H**_**6**_^**2+**^: In a N_2_ filled glovebox, *n*-Hexane/THF mixed solvent (v:v = 2:1) (10 mL) was layered onto a colorless THF solution (5 mL) of [Fe­(H_2_O)_6_]­(ClO_4_)_2_ (545 mg, 1.5 mmol). Then, *n*-hexane/THF solution (15 mL) of 4,5-dimethyl-1,2-phenylenediamine (612 mg, 44.5 mmol) was layered on this solution. The mixture was stored for 4–5 days at room temperature until crystal suitable for SC-XRD characterization was obtained. After removal of the solvent, the product was washed with THF (2 mL x 2) and dried in vacuum to afford colorless crystals in 45% yield.^1^H NMR (CD_3_CN): 10.20 (s, 18H, C**H**_3_), 17.86 (s, 6H, Ph-**H**). Elemental analysis: Chemical Formula: (C_24_H_36_FeN_6_Cl_2_O_8_ x THF) Calc: C (45.73%); H (6.03%); N (11.43%). Exp: C (45.72%); H (6.06%); N (11.41%).

### Cyclic Voltammetry of Iron Complexes

Three mL of a CH_3_CN solution of the Fe complexes (1 mM) containing 0.1 M of [NBu_4_]­PF_6_ was prepared in the glovebox and were transferred to an electrochemical cell outside the glovebox, which was purged with Ar for 5 min (note: a conventional three-electrode cell was used with a glassy carbon working electrode, an Ag/AgNO_3_ (0.01 M) and platinum wire as the counter electrode). The potentials were measured with respect to the Ag/AgNO_3_ reference electrode and converted to Fc^0/+^ (Fc^0/+^ potential measured under the same experimental conditions). Cyclic voltammograms were obtained at 100 mV/s scan rate. All electrochemical measurements were carried out under Ar atmosphere. See Supporting Information for further details.

### Oxidative Deprotonation and Reductive Protonation of the Fe-Based ECPBs

In the glovebox, stock solutions of appropriate concentrations of the complexes, 1,3,5-trimethoxybenzene (Internal Standard, Int. Std.) and relevant PCET reagents were prepared in CD_3_CN. In a 7-in., 5 mm o.d. NMR tube, a 0.9 mL CD_3_CN solution containing desired concentrations of the complex (1–10 mM, unless otherwise noted) and Int. Std. (4–10 mM, unless otherwise noted) was recorded as the blank solution for each reactivity study. Following the blank spectrum, 0.1 mL (unless otherwise noted) of PCET reagents of desired concentration was added (5–30 mM, unless otherwise noted). Subsequently, the NMR tube was capped and sealed with Teflon tape to inhibit any unanticipated oxidation by O_2_ during reaction time scale. Multiple NMR spectra over time revealed the reactivity of the systems under study. The internal standard peak at 6.12 ppm (Ph-H, 3H) was used as reference for quantification of all components of the reactions. Complexes ^**X**^ **6** ^**2+**^ were quantified using the integration values for singlet N–H peaks corresponding to 6 protons at around 11 ppm. Complexes ^**X**^ **8H**_**2**_^**2+**^ were quantified using the average integrations of singlet N–H peaks corresponding to 2 protons each at the 10–13 ppm window and doublet N–H_2_ peaks corresponding to 2 protons each at the 4–6 ppm window. Complexes ^**X**^ **10H**_**4**_^**2+**^, which were observed in situ, were quantified using the average integrations of a singlet N–H peak corresponding to 2 protons at around 11 ppm and a broad N–H_2_ peak corresponding to 8 protons each at the 4–6 ppm window. Complexes ^**X**^ **12H**_**6**_^**2+**^, which were inherently high-spin and paramagnetic, were challenging to quantify in the usual diamagnetic window. The broadness and chemical shifts of the signals would change depending on the concentration (being almost undetectable at <2 mM conc), and in general, yields erroneous quantification as seen in the decrease of mass balances. For quantification of ^**X**^ **12H**_**6**_^**2+**^ at the end of some reactions, 30 mM bipyridine (bpy) was added to the reaction mixture. Bpy displaces the reduced ligands and ends up forming diamagnetic (low-spin) [Fe^II^(bpy)_3_]^2+^. This gives two handles to quantify the amounts of ^**X**^ **12H**_**6**_^**2+**^ – concentrations of free ligand (= 3­[^**X**^ **12H**_**6**_^**2+**^]) and [Fe^II^(bpy)_3_]^2+^ (= [^**X**^ **12H**_**6**_^**2+**^]). The 6H^+^/6e^–^ yield was based on the formation of oxidized products versus the initial concentration of the substrate. The ECPB mass balance was calculated from the total concentration of the resultant complexes and the of the PCET reagent product (if any) versus their initial concentration of the starting complex and starting PCET reagent (if any), respectively. See further details in the Supporting Information.

### BDFE Calculations from Comproportionation Reactions

The comproportionation reactions were performed by preparing equimolar (10 mM) 1 mL solutions of ^**X**^ **6** ^**2+**^ and ^**X**^ **12H**_**6**_^**2+**^ in CD_3_CN inside a N_2_ filled glovebox. These solutions were transferred into 7-in., 5 mm o.d. NMR tube along of an internal standard (1,3,5-trimethoxybenzene, 10 mM). The concentration of all 4 species of the respective family of the ECPB was closely monitored over time using NMR to calculate equilibrium constants (*K*_eq_). The *K*_eq_ values were then used to calculate the BDFE_avg_. The set of all possible equilibration reactions and their corresponding Keq are shown below. See further details in the Supporting Information.

### General Procedure (Adapted from Protocol Reported by Mayer and coworkers^21^)

In the glovebox, 3 mL of CH_3_CN solution containing 100 mM [Bu_4_N]­PF_6_, 50 mM Pyr.H^+^/Pyr. buffer was prepared, after which desired amount of substrates (0.75 mm of ^**X**^ **6** ^**2+**^ and 1.5 mm of ^**X**^ **8H**_**2**_^**2+**^, X = H, Me, and MeO) was added. For each substrate, open circuit potential measurements were collected at several ratios of the oxidized: reduced form (^**X**^ **6** ^**2+**^: ^**X**^ **8H**_**2**_^**2+**^), ranging between 0.5:1 and 2:1. Under Ar flow, OCP was recorded every second for 5–10 min, or until the potential has stabilized such that it changed less than 1.5 mV over 5 min. After each OCP measurement, Fc (in CH_3_CN) was added as reference before a cyclic voltammetry (CV) measurement was carried out. See Supporting Information for details on the BDFE determination from these measurements.

### Decoupled Oxidation of Diphenylhydrazine

In the glovebox, a 1 mL CD_3_CN-*d*_3_ solution containing 10 mM 1,3,5-trimethoxybenzene and ∼10 mM ^**MeO**^ **6** ^**2+**^ was prepared. After the first ^1^H NMR spectrum (blank) was recorded, ∼30 mM DPH was added to the NMR tube and the reaction was monitored over time. After full consumption of ^**MeO**^ **6** ^**2+**^, O_2_ was bubbled into the solution for 30 s before each subsequent NMR measurement. The complete consumption of ^**MeO**^ **12H**_**6**_^**2+**^ and the consequent formation of ^**MeO**^ **10H**_**2**_^**2+**^, ^**MeO**^ **8H**_**4**_^**2+**^ and finally ^**MeO**^ **6** ^**2+**^ mark the completion of the first cycle. After the first cycle, the solution was degassed and purged with N_2_, transferred into the glovebox and reacted with another 3 equiv of DPH calculated based on recovered ECPB. This strategy was repeated for one more cycle and the results are shown in the Supporting Information.

### Buffering Experiments

In the glovebox, a 1 mL CD_3_CN solution containing 10 mM 1,3,5-trimethoxybenzene and equimolar amounts of ^**X**^ **6** ^**2+**^ (∼10 mM) and ^**X**^ **12H**_**6**_^**2+**^ (∼10 mM) was prepared and transferred into an NMR tube. ^1^H NMR spectra (blank) were recorded over time until no changes in concentration were observed, i.e., until the ECPB system equilibrated. After the solution equilibrated, ∼5 mM PCET regent (either 1,4-benzoquinone (BQ) or 4,5-MeO-1,4-hydroquinone ((MeO)_2_-H_2_Q)) was added to the NMR tube and the reaction was again monitored over time to observe the shifts in equilibria. The experiments were performed at room temperature. See further details in the Supporting Information.

### Kinetics of PCET Reactions

A 1 mL CD_3_CN solution containing 10 mM 1,3,5-trimethoxybenzene and ∼2 mM ^**Me,H**^ **5** ^**+**^ (Cu)/^**Me**^ **6** ^**2+**^ (Fe) was prepared inside a glovebox. After the first ^1^H NMR spectrum (blank) was recorded, 20 mM DPH was added to each sample in order to study pseudo first order kinetics. The reaction (consumption of the complex and formation of DPD) was monitored over time. The experiments were performed at room temperature. See further details in the Supporting Information.

## Supplementary Material



## Notes

Experimental details, including synthetic protocols, characterization data, spectra, crystallographic data (CCDC numbers: 2445100–2445107), and electrochemical measurements and reactivity experiments (PDF)
