# Supplementary material for: Stepwise 6H+/6e– Electron-Coupled Proton Buffers Based on Fe and Redox-Active Ligands
Source: Inorg Chem. 2025 Sep 22;64(39):19632–46. doi: 10.1021/acs.inorgchem.5c02720 (PMC12505259; doi:10.1021/acs.inorgchem.5c02720)
Supplement: Supplementary file 1 [file ic5c02720_si_001.pdf]

Supporting Information for:

Stepwise 6H$^+$/6e$^-$ Electron-Coupled-Proton Buffers based on Fe and Redox-Active Ligands

Rajdeep Sarma,[a] Tong Wu,[a] Daniel Ye,[a] Yi Lin Qiu,[a] Serim Park[a], Emma Cohen[a], Jin Xiong[a], Maxime A. Siegler[b], Yisong Guo[a,*] and Isaac Garcia-Bosch [a,*]

[a]Department of Chemistry, Carnegie Mellon University, Pittsburgh, Pennsylvania 15213, United States.

[b]Johns Hopkins University, Baltimore, Maryland 21218, United States

igarciab@andrew.cmu.edu

ysguo@andrew.cmu.edu

Table of Contents

1. Physical methods and materials……………………………………………………...…S3

2. Naming of the complexes……………………………………………………………………S4

3. Synthesis and characterization of Fe complexes………………………………….…S5

4. Electrochemical and spectroscopic characterization.….……………………….……S35

5. Oxidative deprotonation and reductive protonation of ECPBs………………………S39

6. Thermochemistry…………………………………………………………………..………S48

7. Decoupled oxidation of diphenylhydrazine……………………………………..………S62

8. Buffering experiments…………………………………………………………………..…S64

9. Kinetics………………………………………………………………………………..…..S65

10. References…………………………………………………………………………………S66

1. Physical methods and materials.

Reagents: All reagents and solvents were purchased at the highest level of purity and used as received except as noted.

Solvents were purified and dried by passing through an activated alumina purification system (INERT Pure Solv) or by conventional distillation techniques.

Glovebox: synthesis of copper complexes and preparation of some NMR samples was carried out under anaerobic conditions in an mBRAUN MB-Unilab Pro SP Glovebox system.

UV-Vis measurements were carried out by using a Hewlett Packard 8454 diode array spectrophotometer with a 10 mm path quartz cell. The spectrometer was equipped with HP Chemstation software and a Unisoku cryostat for low temperature experiments.

NMR spectra were recorded in 7-inch, 5-mm o.d. NMR tubes on a 500 MHz NMR (Bruker AvanceTM 500) to acquire spectra with 32 cumulative scans.

SC-XRD measurements: Reflection intensities were measured at 110 K using either a SuperNova diffractometer (with Atlas detector) with Mo Kα radiation (λ = 0.71073 Å) or Cu Kα radiation (λ = 1.54178 Å) or a Rigaku XtaLAB Synergy R diffractometer (with a rotating-anode X-ray source and HyPix-6000HE detector) with Cu Kα radiation (λ = 1.54178 Å). Data collection, refinement of cell dimensions, and data reduction were performed using the program CrysAlisPro (see SI for more details).

Mössbauer Spectroscopy: ^{57}*Fe Mössbauer spectra were collected on powder samples using two spectrometers employing Janis Research (Wilmington, MA) SuperVaritemp dewars equipped with a LakeShore Model 331 A temperature controller. The external magnetic field of 0.045 T was provided by a permanent magnet, while the external fields of 0.1 T and 7 T were provided by a superconducting magnet. Powder samples were prepared by grinding a mixture of crystals and boron nitride (BN), and transferring the resulting powders into sample holders manufactured by polyoxymethylene. A threaded lid was used to apply compression and fix the powders. To achieve effective seal and protect the samples from air, vacuum grease was applied to the threaded lid. The data was simulated using quadrupole doublet model with Lorentzian line shapes. The asymmetric parameter is defined as* $\eta = (V_{xx}-V_{yy})/V_{zz}$*, where* V_{xx}*,* V_{yy} *and* V_{zz} *are the three principal values of the electron field gradient tensor. Isomer shift values are referenced against that of α-Fe foil at 298 K.*

Elemental analysis was performed by Robertson Microlit Laboratories, 1705 US Highway 46, Suite 1D, Ledgewood, NJ, 07852 and Midwest Microlab, 7212 Shadeland Ave 110, Indianapolis, IN 46250.

Electrochemical measurements were carried out on a model 620E Electrochemical Workstation (CH Instruments).

2. Naming of the complexes

Complex naming is based on the number of electrons and protons available for Proton-coupled electron transfers (PCET). For example, **^{MeO}Fe(12H^{+}/12e^{-})$^{2+}$** is formulated as a Fe^{II} ion (d^6, 6 electrons) bound by three fully reduced *o*-phenylenediamine ligands (catecholate-like), which can undergo oxidation to the semiquinone-like and quinone-like states (2 electrons per ligand, 6 total electrons). This ligand scaffold also has 12 protons on N coordinating to the Fe^{II} center (see Figure below). **^{MeO}Fe(12H^{+}/12e^{-})$^{2+}$** is named based on total number of electrons (6 from Fe and 6 from ligand, 12 total) and protons (4 protons from each ligand, 12 total). For brevity, **^{MeO}Fe(12H^{+}/12e^{-})$^{2+}$** is named **^{MeO}12^{2+}**.

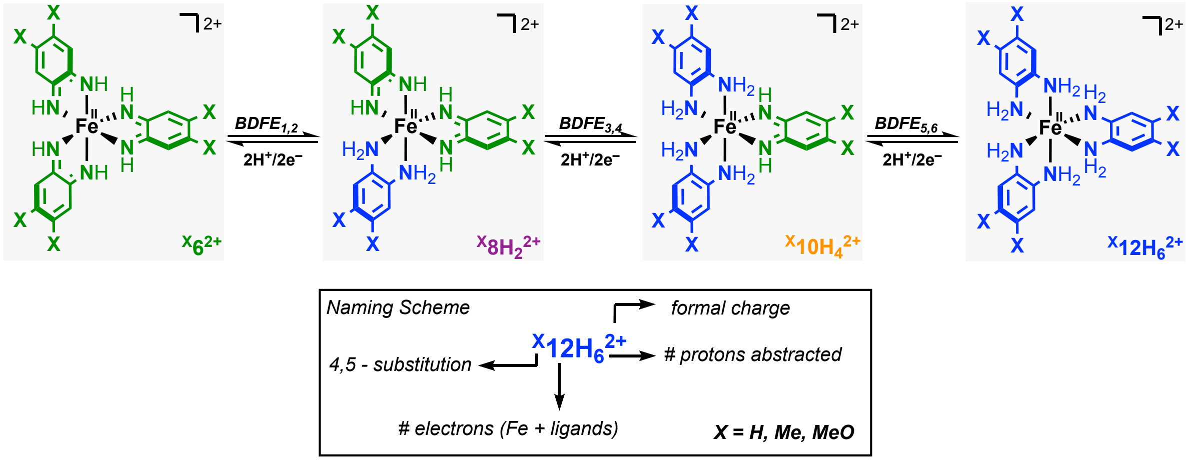

Figure S1. Naming scheme of the Fe complexes described in the paper.

3. Synthesis and characterization of Fe complexes

4,5-dimethoxy-1,2-phenylenediamine and **4,5-dimethyl-1,2-phenylenediamine** were synthesized following a reported procedure.[1]

Synthesis of $^{\mathrm{H}}\mathbf{6}^{2+}$, $^{\mathrm{H}}\mathbf{8H_2}^{2+}$, $^{\mathrm{H}}\mathbf{10H_4}^{2+}$ and $^{\mathrm{H}}\mathbf{12H_6}^{2+}$ were modified from reported methods.[2, 3] We attempted to synthesize $^{\mathrm{MeO}}\mathbf{10H_4}^{2+}$ but the resulting material contained mainly $^{\mathrm{MeO}}\mathbf{6}^{2+}$, $^{\mathrm{MeO}}\mathbf{8H_2}^{2+}$, and some $^{\mathrm{MeO}}\mathbf{12H_6}^{2+}$.

Evans method measurements for paramagnetic complexes: An exact amount of $^{\mathrm{H}}\mathbf{12H_6}^{2+}$, $^{\mathrm{Me}}\mathbf{12H_6}^{2+}$ or $^{\mathrm{Me}}\mathbf{12H_6}^{2+}$ (~6 mM) in CD_3CN containing DCM (100 mM) was placed in an NMR tube with a coaxial inner tube containing blank solvent (CD_3CN containing DCM (100 mM)). ^{1}H-NMR spectra were recorded at 298 K, and the chemical shift of the DCM peak in the presence of the iron complexes was compared to that of the DCM peak in the inner tube containing only the DCM standard. The effective spin-only magnetic moment was calculated by a simplified Evans method analysis according to the following equation:

$$\mu_{eff} = 0.0618\sqrt{\frac{\Delta\nu T}{2fM}}$$

where f is the oscillator frequency (MHz) of the superconducting spectrometer, T is the temperature (K), M is the molar concentration of the paramagnetic metal complex, and $\Delta\nu$ is the difference in frequency (Hz) between the two reference (DCM) signals.

3.1. $^{\mathrm{H}}\mathbf{6}^{2+}$

In a N_2 filled glovebox, 1,2-phenylenediamine (972 mg, 9 mmol) was charged in a 50 mL round-bottom flask and dissolved in 25 mL CH_3CN, then $[Fe(H_2O)_6](ClO_4)_2$ (360 mg, 1 mmol) and 5 g of Molecular Sieves (4A) were added as solid. Then the pale brown solution was transferred out of the glovebox and reacted with oxygen with an O_2 balloon fitted on top of the vial. The color of the solution rapidly turned navy blue in 1 minute, then slowly turned purple in 3 hours. The reaction stopped until the color of the solution was bright green after 2 days. After filtration, the solvent was removed under vacuum. The crude product was crystallized by by either vapor diffusion of Et_2O into a CH_3CN solution containing the crude or layering Et_2O on top of a concentrated CH_3CN solution of $^{\mathrm{H}}\mathbf{6}^{2+}$ (1.50 g, 80% yield) to afford crystal suitable for SC-XRD characterization.

^{1}H-NMR (CD_3CN): 7.02 (s, 6H, Ph-**H**), 7.10 (s, 6H, Ph-**H**), 11.79 (s, 6H, N**H**).

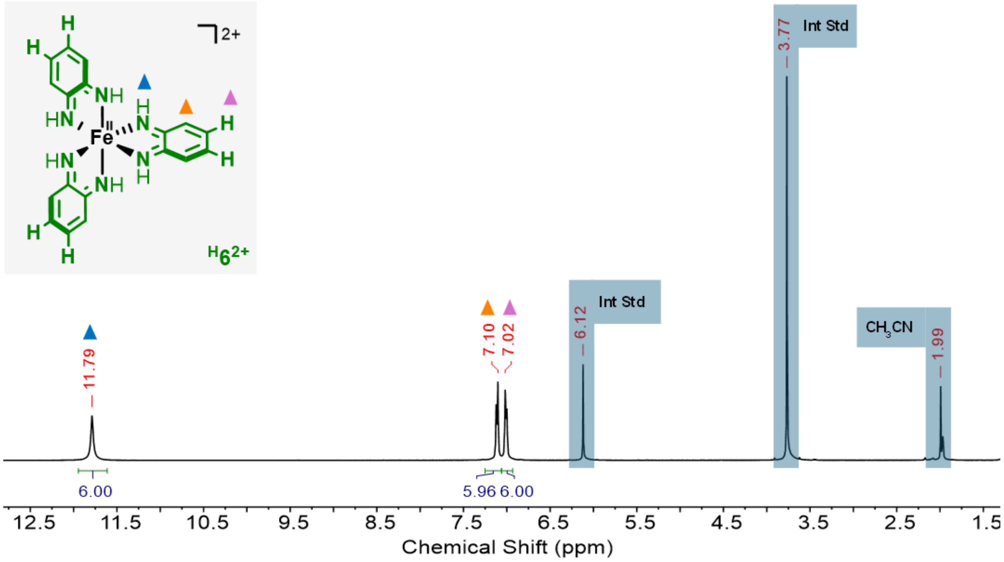

Figure S2. ^{1}H-NMR spectrum of $^{\mathbf{H}}\mathbf{6}^{2+}$ in CD_3CN. Int. Std. = 1,3,5 – trimethoxybenzene.

Elemental analysis: Chemical Formula: ($C_{18}H_{18}FeN_6Cl_2O_8$ x $0.5CH_3CN$). Calc. C: 38.44%; H 3.31%; N 15.34%. exp. C 38.52%; H 3.08%; N 15.11%.

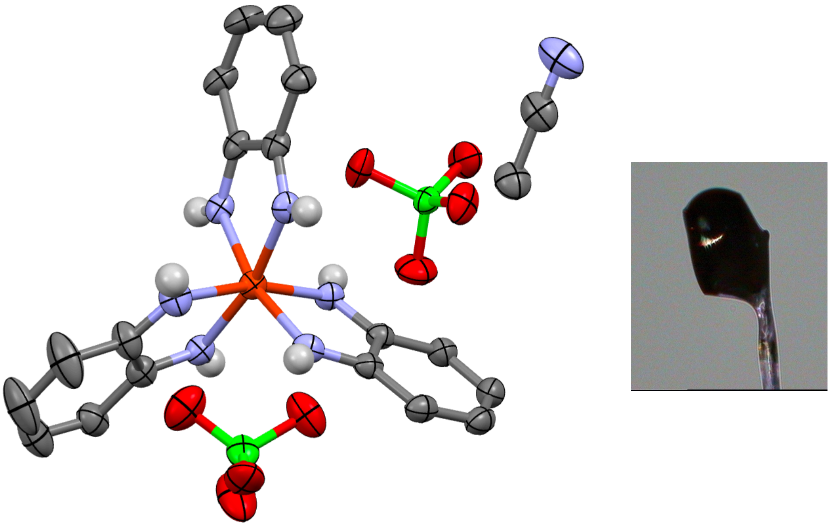

Figure S3. Displacement ellipsoid plot (50% probability level) of $^{\mathbf{H}}\mathbf{6}^{2+}$ at 110 (K). Selected H atoms were removed for clarity.

All reflection intensities were measured at 110 K using a SuperNova diffractometer (equipped with Atlas detector) with Cu *K*α radiation (λ = 1.54178 Å) under the program CrysAlisPro (Version CrysAlisPro 1.171.42.49, Rigaku OD, 2022). The same program was used to refine the cell dimensions and for data reduction. The structure was solved with the program SHELXS-2018/3 (Sheldrick, 2018) and was refined on F^2 with SHELXL-2018/3 (Sheldrick,

2018). Analytical numeric absorption correction using a multifaceted crystal model was applied using CrysAlisPro. The temperature of the data collection was controlled using the system Cryojet (manufactured by Oxford Instruments). The H atoms were placed at calculated positions (unless otherwise specified) using the instructions AFIX 43 or AFIX 137 with isotropic displacement parameters having values 1.2 or 1.5 U_{eq} of the attached C or N (only for N1F/N2F/N1G/N2G) atoms. The H atoms attached to N1X and N2X (X = A-E) were found from difference Fourier maps, and their coordinates were refined pseudofreely so that the N–H bond distances were found within an acceptable range.

The asymmetric unit contains two crystallographically independent Fe complexes, four perchlorate counterions and two lattice acetonitrile solvent molecules. The structure is partly disordered. One of the 1,2-Diaminobenzene ligand and three perchlorate counterions are disordered over either two or three orientations. All occupancy factors can be retrieved from the final .cif file.

Table S1. Crystallographic data for $^{H}\mathbf{6}^{2+}$

Crystal data	
Chemical formula	$C_{18}H_{18}FeN_6\cdot 2(ClO_4)\cdot C_2H_3N$
M_r	614.19
Crystal system, space group	Orthorhombic, *Pbca*
Temperature (K)	110
a, *b*, *c* (Å)	14.77749 (19), 15.1061 (2), 45.4778 (6)
V (Å^3)	10152.0 (2)
Z	16
Radiation type	Cu $K\alpha$
μ (mm^{-1})	7.24
Crystal size (mm)	0.39 × 0.28 × 0.28
Data collection	
Diffractometer	SuperNova, Dual, Cu at zero, Atlas
Absorption correction	Analytical<br>*CrysAlis PRO* 1.171.42.49 (Rigaku Oxford Diffraction, 2022) Analytical numeric absorption correction using a multifaceted crystal model based on expressions derived by R.C. Clark & J.S. Reid. (Clark, R. C. & Reid, J. S. (1995). Acta Cryst. A51, 887-897) Empirical absorption correction using spherical harmonics, implemented in SCALE3 ABSPACK scaling algorithm.
T_{min}, T_{max}	0.144, 0.321
No. of measured, independent and observed [$I > 2\sigma(I)$] reflections	70046, 9958, 9039
R_{int}	0.039
$(\sin \theta/\lambda)_{max}$ (Å^{-1})	0.616
Refinement	
$R[F^2 > 2\sigma(F^2)]$, $wR(F^2)$, S	0.045, 0.109, 1.09
No. of reflections	9958
No. of parameters	976
No. of restraints	994
H-atom treatment	H atoms treated by a mixture of independent and constrained refinement
	$w = 1/[s^2(F_o^2) + (0.0405P)^2 + 10.5614P]$, where $P = (F_o^2 + 2F_c^2)/3$
$\Delta\rho_{max}$, $\Delta\rho_{min}$ (e Å^{-3})	0.40, -0.52

3.2. $^{H}8H_2^{2+}$

In a N_2 filled glovebox, *ortho*-phenylenediamine (324 mg, 3 mmol) was charged in a 30 mL vial and dissolved in 12 mL CH_3CN, then $[Fe(H_2O)_6](ClO_4)_2$ (360 mg, 1 mmol) was added as solid. Then the pale brown solution was transferred out the glovebox, reacting with oxygen in air. The color of the solution rapidly turned navy blue in 10 seconds, then slowly turned purple in 1 hour. During the reaction, NMR was taken to monitor the formation of $^{H}8H_2^{2+}$. Upon completion, the reaction was quenched by removal of the solvent under vacuum to obtain the crude product (the crude is susceptible to contain the over-oxidized product $^{H}6^{2+}$ as an impurity). In a N_2-filled glovebox, the crude product was crystallized by either vapor diffusion of Et_2O into a CH_3CN solution containing the crude or layering Et_2O on top of a CH_3CN solution containing the crude to afford dark crystals suitable for SC-XRD characterization. Yield: ~ 80%.

^{1}H-NMR (CD_3CN): 4.41 (d, 2H, N**H**$_2$), 5.41 (d, 2H, N**H**$_2$), 6.95 (d, 2H, Ph-**H**), 7.12 (t, 2H, Ph-**H**), 7.20 (t, 2H, Ph-**H**), 7.33 (m, 4H, Ph-**H**), 7.38 (d, 2H, Ph-**H**), 10.93 (s, 2H, N**H**), 12.75 (s, 2H, N**H**).

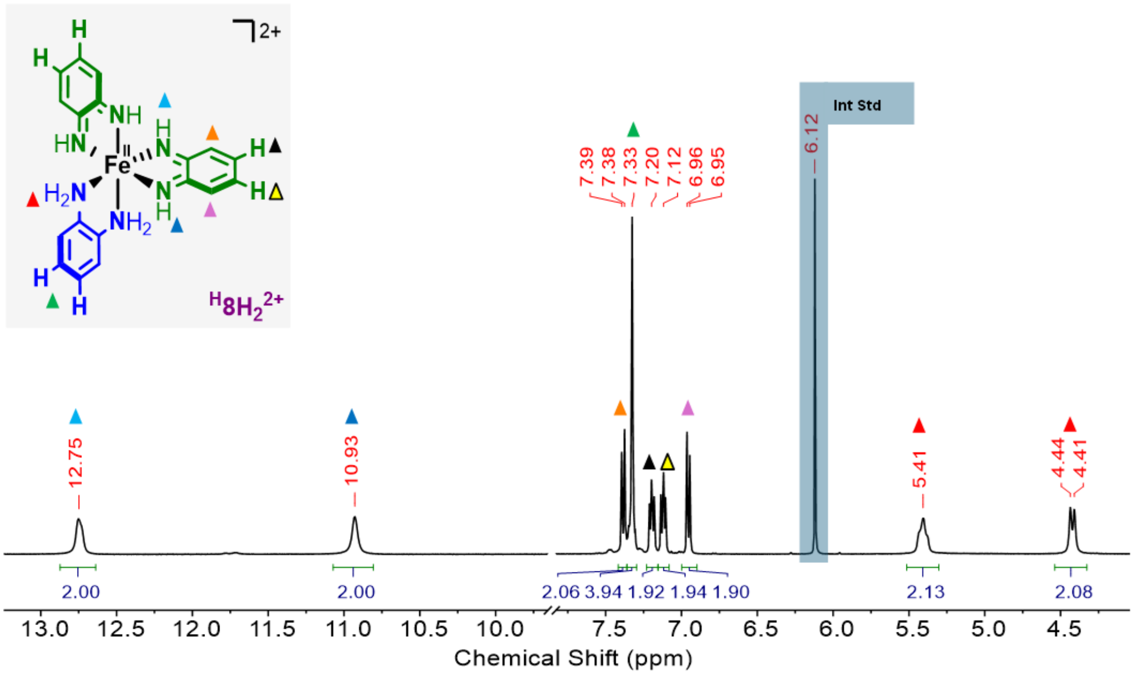

Figure S4. ^{1}H-NMR spectrum of $^{H}8H_2^{2+}$ in CD_3CN. Int. Std. = 1,3,5 – trimethoxy-benzene.

Elemental analysis: Chemical Formula: ($C_{18}H_{20}FeN_6Cl_2O_8$). calc: C (37.59%); H (3.51%); N (14.61%). exp: C (37.50%); H (3.16%); N (14.19%)

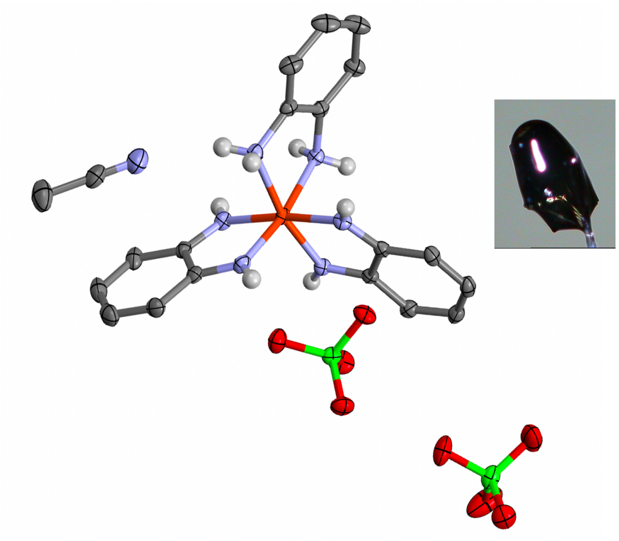

Figure S5. Displacement ellipsoid plot (50% probability level) of $^{\mathbf{H}}\mathbf{8H_2}^{\mathbf{2+}}$ at 110 (K). Selected H atoms were removed for clarity.

All reflection intensities were measured at 110 K using a SuperNova diffractometer (equipped with Atlas detector) with Cu *K*α radiation (λ = 1.54178 Å) under the program CrysAlisPro (Version CrysAlisPro 1.171.42.49, Rigaku OD, 2022). The same program was used to refine the cell dimensions and for data reduction. The structure was solved with the program SHELXS-2018/3 (Sheldrick, 2018) and was refined on F^2 with SHELXL-2018/3 (Sheldrick, 2018). Analytical numeric absorption correction using a multifaceted crystal model was applied using CrysAlisPro. The temperature of the data collection was controlled using the system Cryojet (manufactured by Oxford Instruments). The H atoms were placed at calculated positions (unless otherwise specified) using the instructions AFIX 43 or AFIX 137 with isotropic displacement parameters having values 1.2 or 1.5 U_{eq} of the attached C atoms. The H atoms attached to N1X and N2X (X = A-C) were found from different Fourier maps, and their coordinates were refined pseudofreely so that the N–H bond distances were found within an acceptable range.

The asymmetric unit contains one Fe complex, two perchlorate counterions and two lattice acetonitrile solvent molecules. The structure is ordered.

Table S2. Crystallographic data for $^{H}\mathbf{8H_2}^{2+}$

Crystal data	
Chemical formula	$C_{18}H_{20}FeN_6·2(ClO_4)·2(C_2H_3N)$
M_r	657.26
Crystal system, space group	Monoclinic, $P2_1/n$
Temperature (K)	110
a, *b*, *c* (Å)	8.05224 (10), 32.5895 (3), 11.32832 (14)
b (°)	110.5798 (14)
V (Å^3)	2783.05 (6)
Z	4
Radiation type	Cu $K\alpha$
μ (mm^{-1})	6.65
Crystal size (mm)	0.76 × 0.49 × 0.36
Data collection	
Diffractometer	SuperNova, Dual, Cu at zero, Atlas
Absorption correction	Analytical<br>*CrysAlis PRO* 1.171.42.49 (Rigaku Oxford Diffraction, 2022) Analytical numeric absorption correction using a multifaceted crystal model based on expressions derived by R.C. Clark & J.S. Reid. (Clark, R. C. & Reid, J. S. (1995). Acta Cryst. A51, 887-897) Empirical absorption correction using spherical harmonics, implemented in SCALE3 ABSPACK scaling algorithm.
T_{min}, T_{max}	0.069, 0.253
No. of measured, independent and observed [$I > 2\sigma(I)$] reflections	34729, 5431, 5302
R_{int}	0.040
$(\sin \theta/\lambda)_{max}$ (Å^{-1})	0.616
Refinement	
$R[F^2 > 2\sigma(F^2)]$, $wR(F^2)$, S	0.035, 0.092, 1.05
No. of reflections	5431
No. of parameters	397
No. of restraints	8
H-atom treatment	H atoms treated by a mixture of independent and constrained refinement
$\Delta\rho_{max}$, $\Delta\rho_{min}$ (e Å^{-3})	0.50, -0.46

3.3. $^{\mathbf{H}}\mathbf{10H_4^{2+}}$

In a N_2 filled glovebox, 1,2-phenylenediamine (162 mg, 1.5 mmol) was charged in a 30 mL vial and dissolved in 12 mL CH_3CN, then $[Fe(H_2O)_6](ClO_4)_2$ (180 mg, 0.5 mmol) was added as solid. Then the pale brown solution was transferred out the glovebox, reacting with 5.6 mL oxygen stored in a gas-tight syringe. The reaction was stopped 5 minutes after full consumption of O_2. After removal of solvent in vacuum, the crude solid was transferred into glovebox. The solid was analyzed by NMR (see below).

^{1}H-NMR (CD_3CN-*d_3*): 5.2-5.3 (b, 8H, N-**H_2**), 7.2-7.5 (m, 12H, Ph-**H**), 11.76 (s, 2H, N**H**).

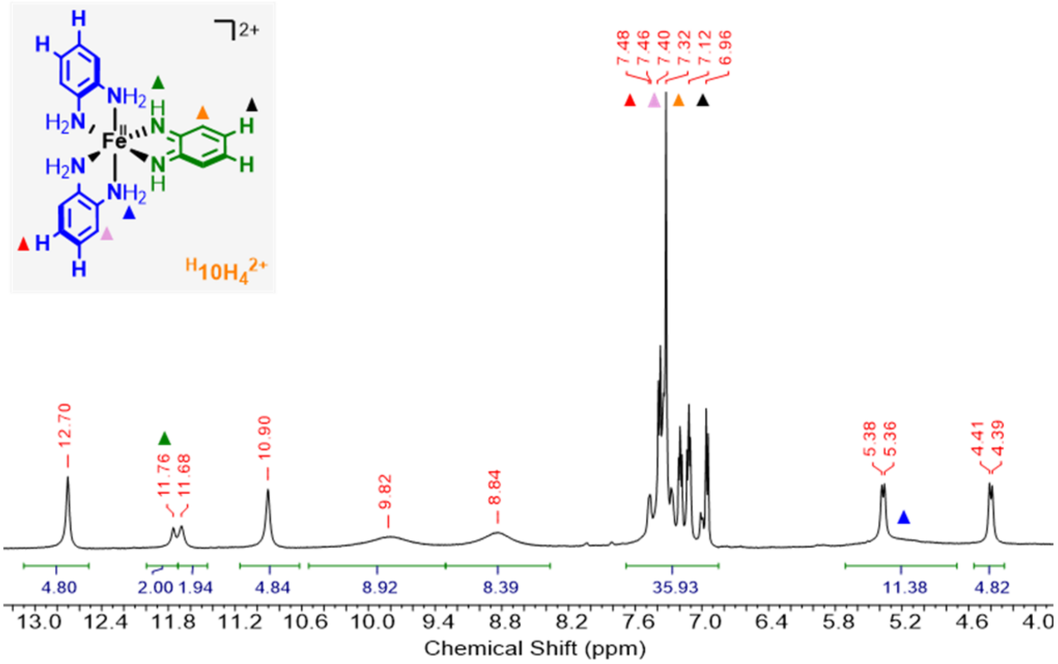

Figure S6. ^{1}H-NMR spectrum of $^{\mathbf{H}}\mathbf{10H_4^{2+}}$ in CD_3CN. $^{\mathbf{H}}\mathbf{8H_4^{2+}}$ (Figure S4) and $^{\mathbf{H}}\mathbf{12H_6^{2+}}$ (Figure S7) peaks can also be seen as disproportionation products of $^{\mathbf{H}}\mathbf{10H_4^{2+}}$. $^{\mathbf{H}}\mathbf{6^{2+}}$ impurities can also be seen (Figure S2).

3.4. $^{H}12H_6^{2+}$

In a N_2 filled glovebox, 2 mL THF solution of $Fe(H_2O)_6(ClO_4)_2$ (1080 mg, 3 mmol) was added dropwise into 5 mL anhydrous THF solution of 1,2-phenylenediamine (972 mg, 9 mmol) over 30 s. Then the reaction was shaken for 15 s and kept still for 2 hours after which the light-yellow solution was removed, and the yielded white crystal was washed with around 5 mL of hexane. After drying in vacuum for an hour, white solid was obtained (80% yield).

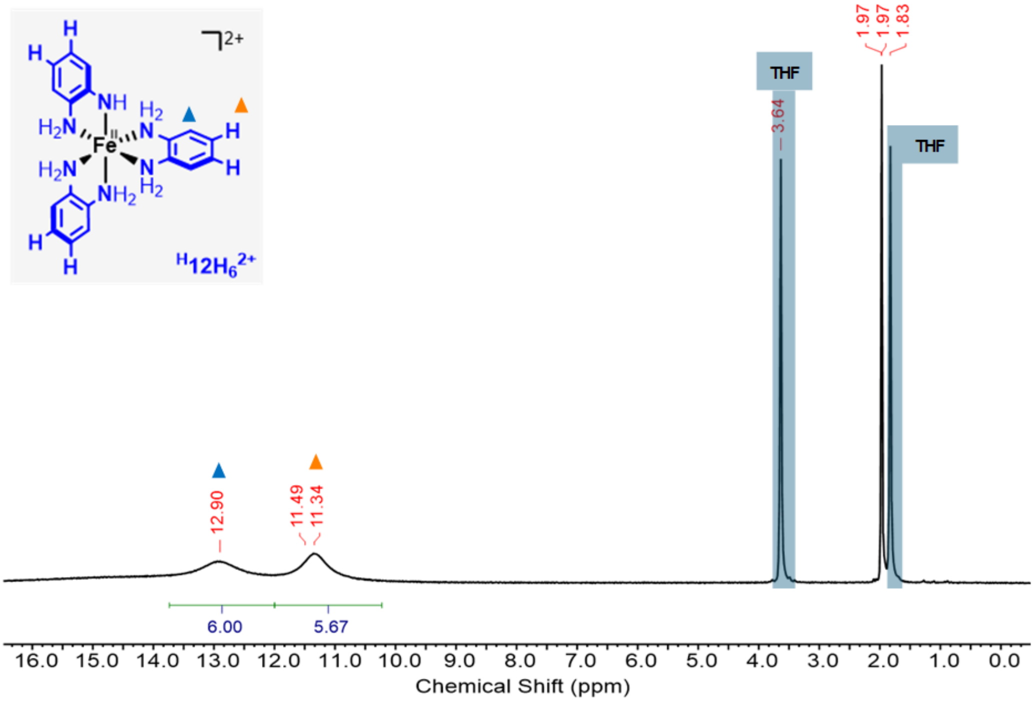

Figure S7. ^{1}H-NMR spectrum of $^{H}\mathbf{12H_6^{2+}}$ in CD_3CN. Note: magnetic susceptibility measurements were carried out using the Evans method. The effective magnetic moment measured, μ_{eff} = 5.30 μB, is consistent with an high-spin iron(II) complex (4 unpaired electrons).

3.5. $^{Me}6^{2+}$

In a N_2 filled glovebox, 4,5-dimethyl-1,2-phenylenediamine (408 mg, 3 mmol) was charged in a 30 mL vial and dissolved in 12 mL CH_3CN, then $[Fe(H_2O)_6](ClO_4)_2$ (360 mg, 1 mmol) was added as solid. Then the pale brown solution was transferred out of the glovebox and reacted with oxygen with an O_2 balloon fitted on top of the vial in the presence of 4A molecular sieves. The color of the solution rapidly turned navy blue in 15 seconds, then slowly turned purple in 1 hour. The reaction takes about 2-3 days for completion. It was monitored by NMR and stopped when there was only $^{Me}6^{2+}$ in the solution. This could also be monitored visually – a bright green solution is indicative of the end point. The solution was filtered to get rid of molecular sieves and the solvent removed under vacuum. The crude product was crystallized by layering hexane on top of concentrated THF solution of $^{Me}6^{2+}$ to afford crystal suitable for SC-XRD characterization (80% yield). Vapor diffusion of Et_2O into a concentrated solution of $^{Me}6^{2+}$ in CH_3CN also yielded good crystals.

^{1}H-NMR (CD_3CN): 2.21 (s, 18H, C**H**$_3$), 6.80 (s, 6H, Ph-**H**), 11.27 (s, 6H, N**H**).

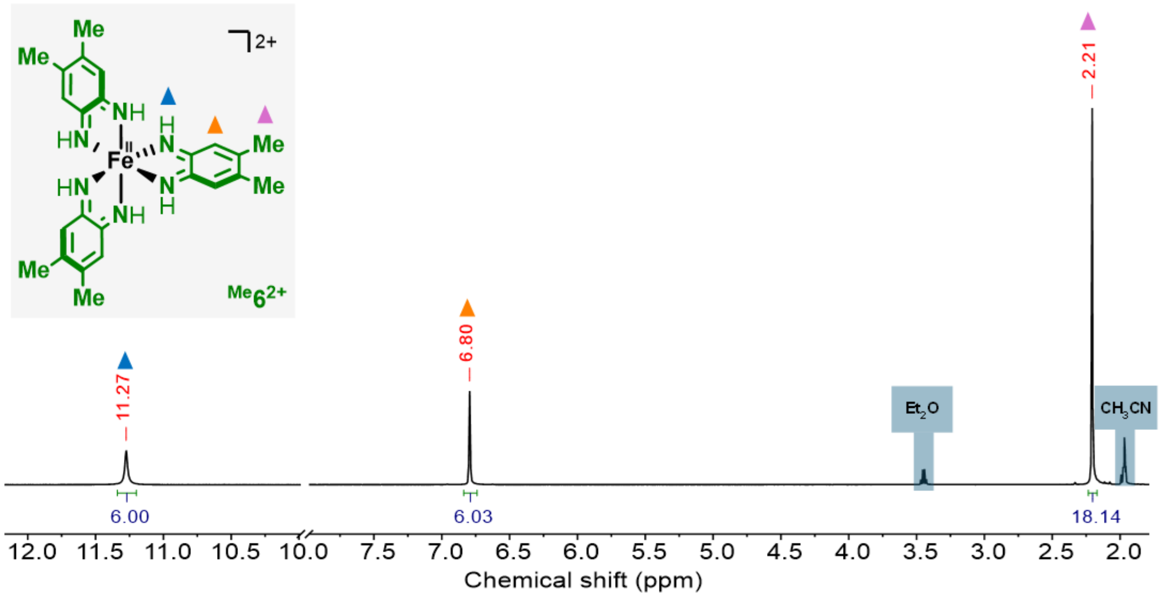

Figure S8. ^{1}H-NMR spectrum of ^{Me}**6**$^{2+}$ in CD_3CN.

Elemental analysis: Chemical Formula: ($C_{24}H_{30}FeN_6Cl_2O_8$ H_2O). calc: C (42.69%); H (4.78%); N (12.45%). exp: C (42.69%); H (4.59%); N (12.13%)

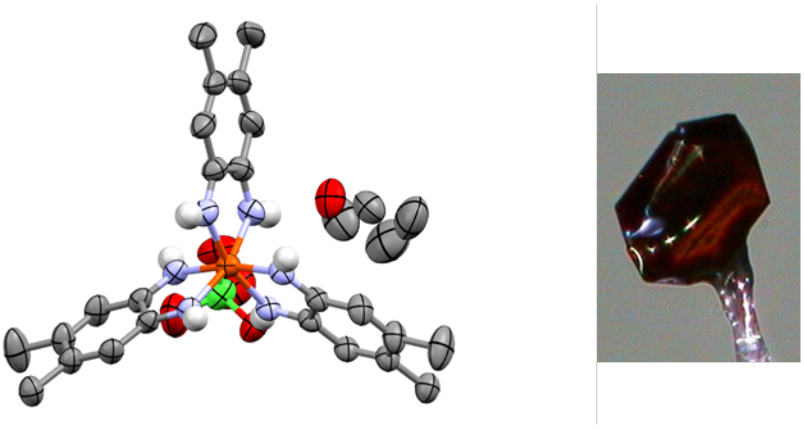

Figure S9. Displacement ellipsoid plot (50% probability level) of ^{Me}**6**$^{2+}$at 193 (K). Selected H atoms were removed for clarity.

All reflection intensities were measured at 193(2) K* using a SuperNova diffractometer (equipped with Atlas detector) with Cu *K*α radiation (λ = 1.54178 Å) under the program CrysAlisPro (Version CrysAlisPro 1.171.42.49, Rigaku OD, 2022). The same program was used to refine the cell dimensions and for data reduction. The structure was solved with the program SHELXS-2018/3 (Sheldrick, 2018) and was refined on F^2 with SHELXL-2018/3 (Sheldrick, 2018). Analytical numeric absorption correction using a multifaceted crystal model was applied using CrysAlisPro. The temperature of the data collection was controlled using the system Cryojet (manufactured by Oxford Instruments). The H atoms were placed at calculated positions (unless otherwise specified) using the instructions AFIX 43 or AFIX 137 with isotropic displacement parameters having values 1.2 or 1.5 U_{eq} of the attached C atoms. The H atoms attached to N1A, N2A and N1B were found from difference Fourier maps, and their coordinates were refined pseudofreely using the DFIX instruction in order to keep the N–H bond distances within an acceptable range.

*Data were initially collected at 110 K, but a more complicated diffraction pattern with additional weak reflections was observed, thus suggesting a more complicated phase at low temperature. The temperature was raised to 193 K, and the diffraction pattern was clean and all reflections could be indexed using the I-centered monoclinic cell (see experimental details for further details).

The asymmetric unit contains ½ Fe complex (located at one site of twofold axial symmetry), one perchlorate counterion and ½ lattice THF solvent molecule (located at one site of twofold axial symmetry). The structure is partly disordered. The ClO_4^- counterion is disordered over two orientations, and the occupancy factor of the major component of the disorder refines to 0.658(6). The lattice THF solvent molecule is also disordered as it is found at one site of twofold axial symmetry, and its occupancy factor was constrained to be 0.5.

Table S3. Crystallographic data for ^{Me}**6**$^{2+}$.

Crystal data	
Chemical formula	$C_{24}H_{30}FeN_6·2(ClO_4)·C_4H_8O$
M_r	729.39
Crystal system, space group	Monoclinic, *I*2/*a*
Temperature (K)	193
a, *b*, *c* (Å)	10.90252 (16), 19.2002 (3), 15.8091 (2)
β (°)	92.3780 (13)
V (Å^3)	3306.48 (8)
Z	4
Radiation type	Cu *K*α
μ (mm^{-1})	5.66
Crystal size (mm)	0.37 × 0.26 × 0.14
Data collection	
Diffractometer	SuperNova, Dual, Cu at zero, Atlas
Absorption correction	Analytical *CrysAlis PRO* 1.171.42.49 (Rigaku Oxford Diffraction, 2022) Analytical numeric absorption correction using a multifaceted crystal model based on expressions derived by R.C. Clark & J.S. Reid. (Clark, R. C. & Reid, J. S. (1995). Acta Cryst. A51, 887-897) Empirical absorption correction using spherical harmonics, implemented in SCALE3 ABSPACK scaling algorithm.
T_{min}, T_{max}	0.310, 0.600
No. of measured, independent and observed [$I > 2\sigma(I)$] reflections	13234, 3247, 3055
R_{int}	0.020
$(\sin \theta/\lambda)_{max}$ (Å^{-1})	0.616
Refinement	
$R[F^2 > 2\sigma(F^2)]$, $wR(F^2)$, S	0.042, 0.125, 1.07
No. of reflections	3247
No. of parameters	289
No. of restraints	180
H-atom treatment	H atoms treated by a mixture of independent and constrained refinement
$\Delta\rho_{max}$, $\Delta\rho_{min}$ (e Å^{-3})	0.44, -0.35

3.6. $^{Me}8H_2^{2+}$

In a N_2 filled glovebox, 4,5-dimethyl-1,2-phenylenediamine (408 mg, 3 mmol) was charged in a 30 mL vial and dissolved in 12 mL CH_3CN, then $[Fe(H_2O)_6](ClO_4)_2$ (360 mg, 1 mmol) was added as solid. Then the pale brown solution was transferred out the glovebox, reacting with oxygen in air. The color of the solution rapidly turned navy blue in 10 seconds, then slowly turned purple in 1 hour. During the reaction, NMR was taken to monitor the formation of $^{Me}8H_2^{2+}$. Upon completion, the reaction was quenched by removal of the solvent under vacuum to obtain the crude product (usually the crude contains ~5% $^{Me}6^{2+}$ due to over-oxidation). In a N_2-filled glovebox, the crude product was crystallized by layering hexane on top of a THF solution to afford dark crystals suitable for SC-XRD characterization (40% yield).

^{1}H-NMR (CD_3CN): 2.16 (s, 6H, C**H**$_3$), 2.21 (s, 6H, C**H**$_3$), 2.25 (s, 6H, C**H**$_3$), 4.14 (d, 2H, N**H**$_2$), 5.14 (d, 2H, N**H**$_2$), 6.74 (s, 2H, Ph-**H**), 7.04 (s, 2H, Ph-**H**), 7.16 (s, 2H, Ph-**H**), 10.44 (s, 2H, N**H**), 12.20 (s, 2H, N**H**).

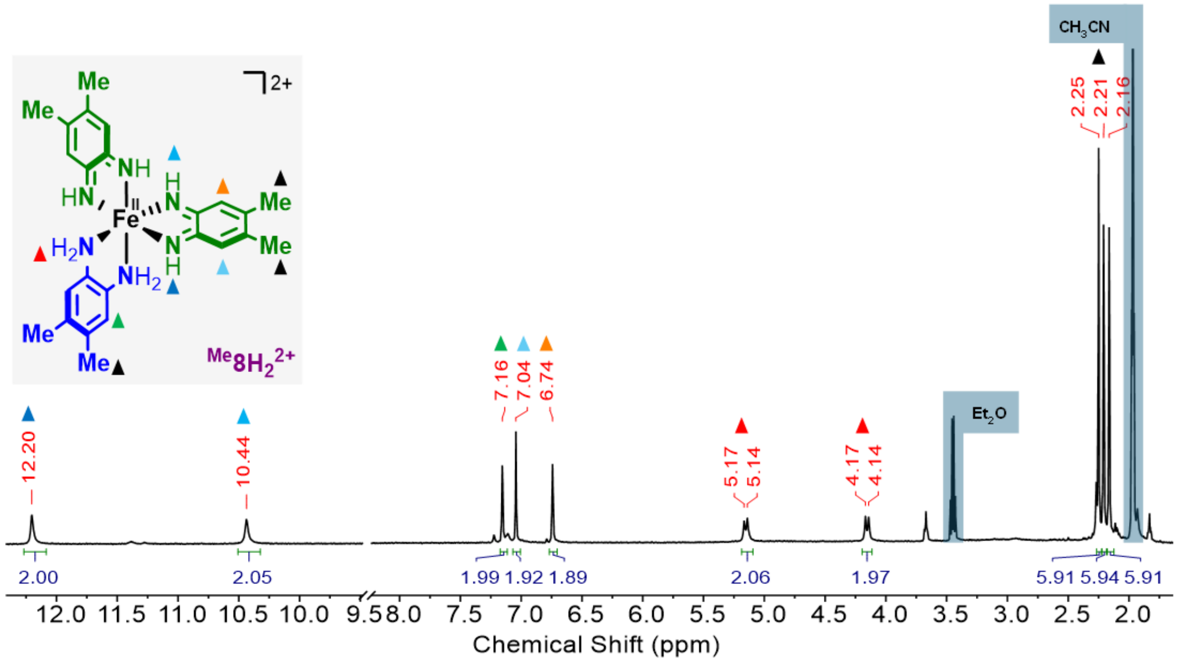

Figure S10. ^{1}H-NMR spectrum of $^{Me}8H_2^{2+}$ in CD_3CN.

Elemental analysis: Chemical Formula: ($C_{24}H_{32}FeN_6Cl_2O_8$ x 2THF x 0.5 hexane). Calc: C (49.66%); H (6.55%); N (9.93%). Exp: C (49.18%); H (6.27%); N (9.96%).

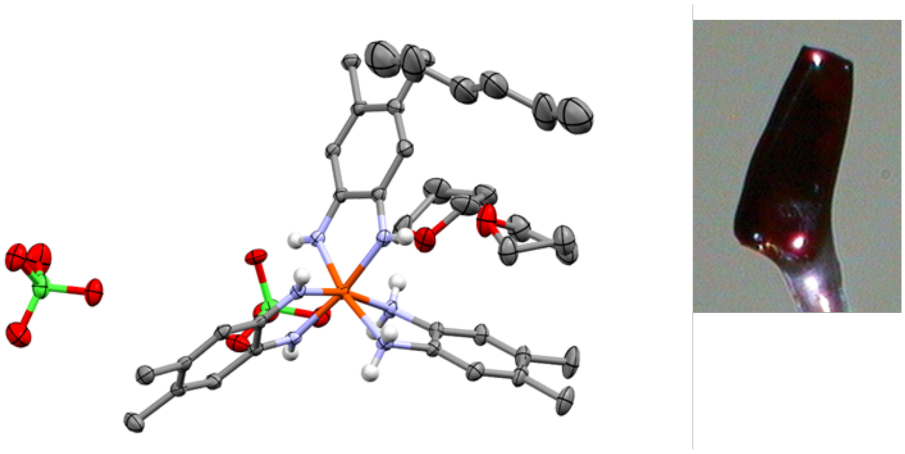

Figure S11. Displacement ellipsoid plot (50% probability level) of $^{\mathbf{Me}}\mathbf{8H_2^{2+}}$ at 110 (K). Selected H atoms were removed for clarity.

All reflection intensities were measured at 110(2) K using a SuperNova diffractometer (equipped with Atlas detector) with Mo *K*α radiation (λ = 0.71073 Å) under the program CrysAlisPro (Version CrysAlisPro 1.171.42.49, Rigaku OD, 2022). The same program was used to refine the cell dimensions and for data reduction. The structure was solved with the program SHELXS-2018/3 (Sheldrick, 2018) and was refined on F^2 with SHELXL-2018/3 (Sheldrick, 2018). Numerical absorption correction based on gaussian integration over a multifaceted crystal model was applied using CrysAlisPro. The temperature of the data collection was controlled using the system Cryojet (manufactured by Oxford Instruments). The H atoms were placed at calculated positions (unless otherwise specified) using the instructions AFIX 23, AFIX 43 or AFIX 137 with isotropic displacement parameters having values 1.2 or 1.5 U_{eq} of the attached C atoms. The H atoms attached to N1X and N2X (X = A-C) were found from difference Fourier maps, and their coordinates were refined pseudofreely using the DFIX instruction in order to keep the N–H bond distances within an acceptable range.

The structure is partly disordered. The two lattice THF solvent molecules are disordered over two orientations, and the occupancy factors of the major components of the disorder refine to 0.596(8) and 0.557(7). One lattice hexane solvent molecule is found at one site of inversion symmetry, and thus only one half is found to be crystallographically independent. The occupancy factor was refined freely and its value is 0.820(7) (*i.e.*, there is 0.41 lattice hexane solvent molecule per asymmetric unit).

Table S4. Crystallographic data for $^{Me}\mathbf{8H_2}^{2+}$.

Crystal data	
Chemical formula	$C_{24}H_{32}FeN_6·0.41(C_6H_{14})·2(ClO_4)·2(C_4H_8O)$
M_r	838.84
Crystal system, space group	Triclinic, *P*-1
Temperature (K)	110
a, *b*, *c* (Å)	11.1123 (2), 12.1645 (4), 17.0805 (5)
α, β, γ (°)	105.761 (3), 94.858 (2), 110.305 (2)
V (Å^3)	2042.61 (10)
Z	2
Radiation type	Mo *K*α
μ (mm^{-1})	0.56
Crystal size (mm)	0.35 × 0.12 × 0.11
Data collection	
Diffractometer	SuperNova, Dual, Cu at zero, Atlas
Absorption correction	Gaussian *CrysAlis PRO* 1.171.42.49 (Rigaku Oxford Diffraction, 2022) Numerical absorption correction based on gaussian integration over a multifaceted crystal model Empirical absorption correction using spherical harmonics, implemented in SCALE3 ABSPACK scaling algorithm.
T_{min}, T_{max}	0.481, 1.000
No. of measured, independent and observed [$I > 2\sigma(I)$] reflections	47108, 9397, 7953
R_{int}	0.036
$(\sin \theta/\lambda)_{max}$ (Å^{-1})	0.650
Refinement	
$R[F^2 > 2\sigma(F^2)]$, $wR(F^2)$, S	0.042, 0.113, 1.06
No. of reflections	9397
No. of parameters	611
No. of restraints	328
H-atom treatment	H atoms treated by a mixture of independent and constrained refinement
$\Delta\rho_{max}$, $\Delta\rho_{min}$ (e Å^{-3})	0.95, -0.49

3.7. ^{Me}10H$_4^{2+}$

In a N_2 filled glovebox, 4,5-methyl-1,2-phenylenediamine (205 mg, 1.5 mmol) was charged in a 30 mL vial and dissolved in 12 mL CH_3CN, then $[Fe(H_2O)_6](ClO_4)_2$ (180 mg, 0.5 mmol) was added as solid. Then the pale brown solution was transferred out the glovebox, reacting with 5.6 mL oxygen stored in a gas-tight syringe. The reaction was stopped 5 minutes after full consumption of O_2. After removal of solvent in vacuum, the crude solid was transferred into glovebox. The resulting solid was analzed by NMR and UV-vis spectroscopy (see below).

^{1}H-NMR (CD_3CN-d_3): 2.19 (s, 18H, C**H**$_3$), 6.78 (s, 6H, Ph-**H**), 11.26 (s, 6H, N**H**).

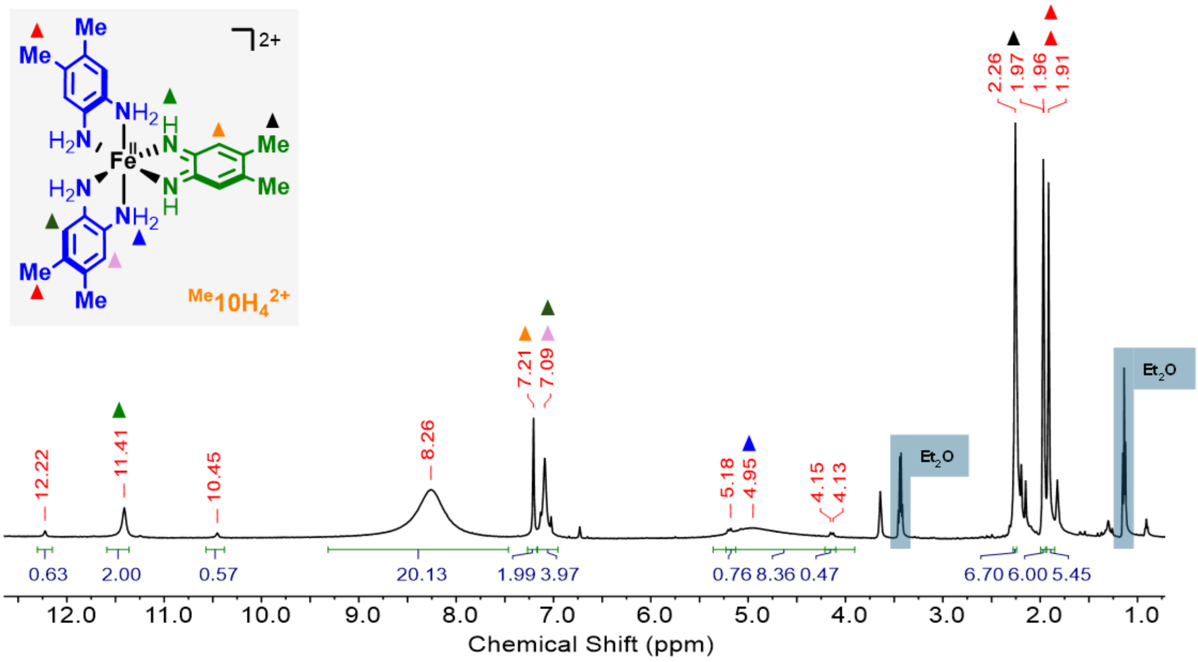

Figure S12. ^{1}H-NMR spectrum of **^{Me}10H$_4^{2+}$** in CD_3CN. Unlabeled peaks can be assigned to **^{Me}12H$_6^{2+}$** (Figure S13) and **^{Me}8H$_2^{2+}$** (Figure S10) because of the disproportionation of **^{Me}10H$_4^{2+}$.**

3.8. $^{Me}12H_6^{2+}$

In a N_2 filled glovebox, n-Hexane/THF mixed solvent (v:v = 2:1) (10mL) was layered onto a colorless THF solution (5 mL) of $[Fe(H_2O)_6](ClO_4)_2$ (545 mg, 1.5 mmol). Then, n-hexane/THF solution (15 mL) of 4,5-dimethyl-1,2-phenylenediamine (612 mg, 44.5 mmol) was layered on this solution. The mixture was stored for 4-5 days at room temperature until crystal suitable for SC-XRD characterization was obtained. After removal of the solvent, the product was washed with THF (2 mL x 2) and dried in vacuum to afford colorless crystals in 45% yield.

^{1}H-NMR (CD_3CN): 10.20 (s, 18H, C**H**$_3$), 17.86 (s, 6H, Ph-**H**).

Elemental analysis: Chemical Formula: ($C_{24}H_{36}FeN_6Cl_2O_8$ x THF) Calc: C (45.73%); H (6.03%); N (11.43%). Exp: C (45.72%); H (6.06%); N (11.41%)

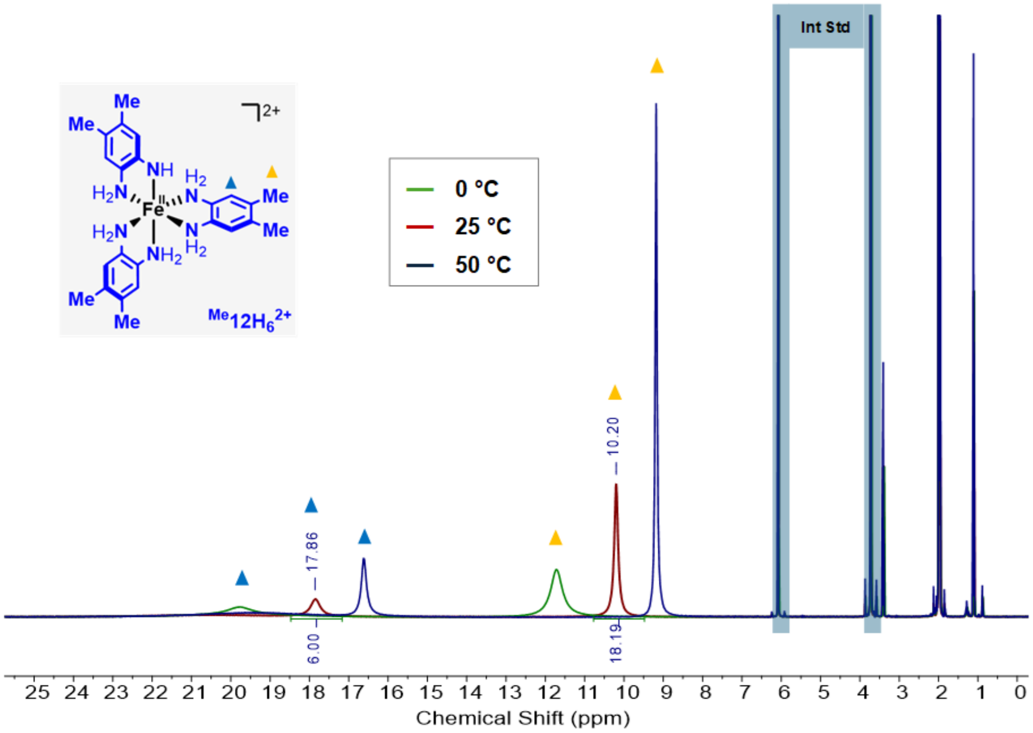

Figure S13. ^{1}H-NMR spectra of high-spin paramagnetic ^{Me}**12H**$_6^{2+}$ in CD_3CN at different temperatures.

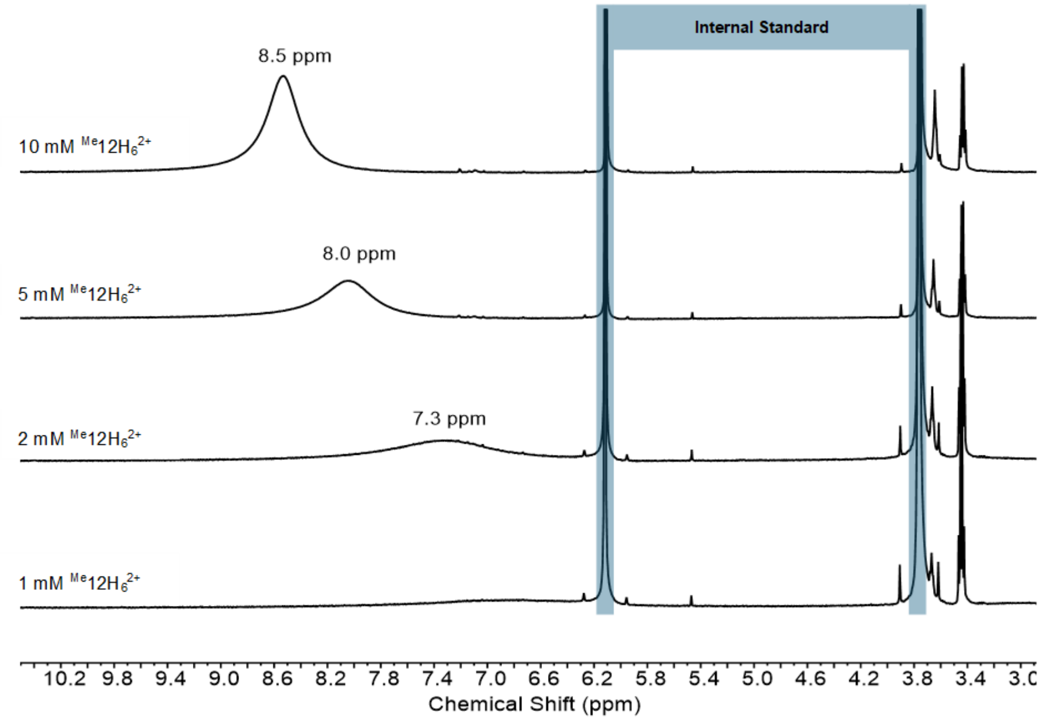

Figure S14. ^{1}H-NMR spectra of high-spin paramagnetic ^{Me}**12H**$_6^{2+}$ in CD_3CN at different concentration. Note: magnetic susceptibility measurements were carried out using the Evans method. The effective magnetic moment measured, μ_{eff} = 5.9 μB, is consistent with an high-spin iron(II) complex (4 unpaired electrons).

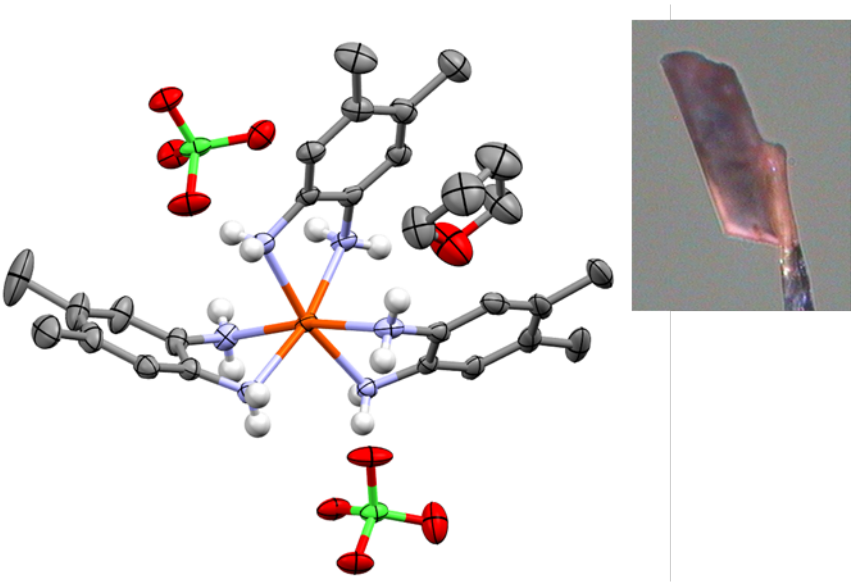

Figure S15. Displacement ellipsoid plot (50% probability level) of $^{\mathbf{Me}}\mathbf{12H_6}^{2+}$ at 110 (K). Selected H atoms were removed for clarity.

All reflection intensities were measured at 110(2) K using a SuperNova diffractometer (equipped with Atlas detector) with Mo *K*α radiation (λ = 0.71073 Å) under the program CrysAlisPro (Version CrysAlisPro 1.171.42.49, Rigaku OD, 2022). The same program was used to refine the cell dimensions and for data reduction. The structure was solved with the program SHELXS-2018/3 (Sheldrick, 2018) and was refined on F^2 with SHELXL-2018/3 (Sheldrick, 2018). Numerical absorption correction based on gaussian integration over a multifaceted crystal model was applied using CrysAlisPro. The temperature of the data collection was controlled using the system Cryojet (manufactured by Oxford Instruments). The H atoms were placed at calculated positions (unless otherwise specified) using the instructions AFIX 43 or AFIX 137 with isotropic displacement parameters having values 1.2 or 1.5 U_{eq} of the attached C atoms. The H atoms attached to N1X and N2X (X = A-C) were found from difference Fourier maps, and their coordinates were refined pseudofreely using the DFIX instruction in order to keep the N–H bond distances within an acceptable range.

The structure is partly disordered. The two ClO_4^- counterions and the lattice THF solvent molecule were found to be disordered over two orientations, and the occupancy factors of the major components of the disorder refine to 0.886(3), 0.900(4) and 0.790(4), respectively.

Table S5. Crystallographic data for **^{Me}12H$_6^{2+}$**.

Crystal data	
Chemical formula	$C_{24}H_{36}FeN_6 \cdot 2(ClO_4) \cdot C_4H_8O$
M_r	735.44
Crystal system, space group	Orthorhombic, *Pbcn*
Temperature (K)	110
a, *b*, *c* (Å)	28.0228 (9), 16.9478 (4), 14.0636 (3)
V (Å^3)	6679.2 (3)
Z	8
Radiation type	Mo $K\alpha$
μ (mm^{-1})	0.67
Crystal size (mm)	0.51 × 0.20 × 0.07
Data collection	
Diffractometer	SuperNova, Dual, Cu at zero, Atlas
Absorption correction	Gaussian<br>*CrysAlis PRO* 1.171.42.49 (Rigaku Oxford Diffraction, 2022) Numerical absorption correction based on gaussian integration over a multifaceted crystal model Empirical absorption correction using spherical harmonics, implemented in SCALE3 ABSPACK scaling algorithm.
T_{min}, T_{max}	0.342, 1.000
No. of measured, independent and observed [$I > 2\sigma(I)$] reflections	61828, 7676, 6307
R_{int}	0.041
$(\sin \theta/\lambda)_{max}$ (Å^{-1})	0.650
Refinement	
$R[F^2 > 2\sigma(F^2)]$, $wR(F^2)$, S	0.044, 0.120, 1.06
No. of reflections	7676
No. of parameters	568
No. of restraints	490
H-atom treatment	H atoms treated by a mixture of independent and constrained refinement
$\Delta\rho_{max}$, $\Delta\rho_{min}$ (e Å^{-3})	0.59, -0.58

3.9. $^{MeO}6^{2+}$

In a N_2 filled glovebox, 4,5-dimethoxyl-1,2-phenylenediamine (505 mg, 3 mmol) was charged in a 30 mL vial and dissolved in 12 mL CH_3CN, then $[Fe(H_2O)_6](ClO_4)_2$ (360 mg, 1 mmol) was added as solid. Then the pale brown solution was transferred out of the glovebox and reacted with oxygen with an O_2 balloon fitted on top of the vial. The solution rapidly turned navy blue the moment it was brought outside the glovebox and in contact with air. The reaction takes about 6 h for completion. It was monitored by NMR and stopped when there was only $^{MeO}6^{2+}$ in the solution. This could also be monitored visually – a bright green solution is indicative of the end point. The solution was filtered to get rid of molecular sieves. The solvent was removed under vacuum. The crude product was crystallized by layering Et_2O on top of a concentrated CH_3CN solution of $^{MeO}6^{2+}$ to afford crystal suitable for SC-XRD characterization (70% yield).

^{1}H-NMR (CD_3CN): 3.87 (s, 18H, O-C**H**$_3$), 6.37 (s, 6H, Ph-**H**), 10.60 (s, 6H, N**H**).

Elemental analysis: Chemical Formula: ($C_{24}H_{30}FeN_6O_6Cl_2O_8$) calc. C (38.27%); H (4.01%); N (11.16%). Exp: C (38.31%); H (4.17%); N (11.51%)

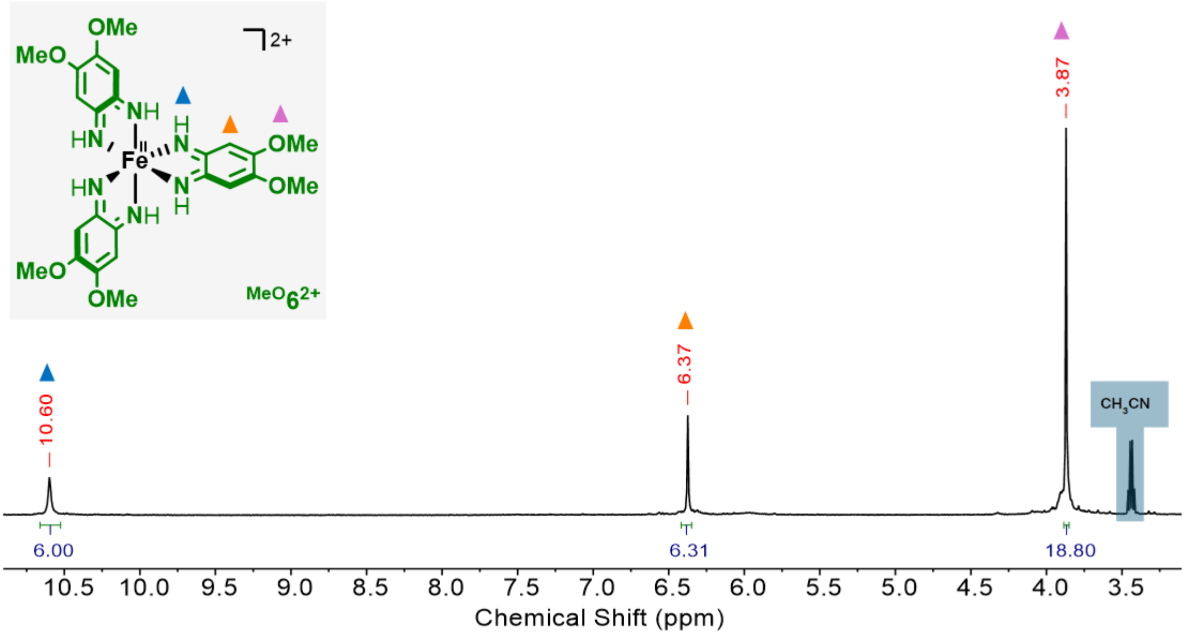

Figure S16. ^{1}H-NMR spectrum of $^{MeO}6^{2+}$ in CD_3CN.

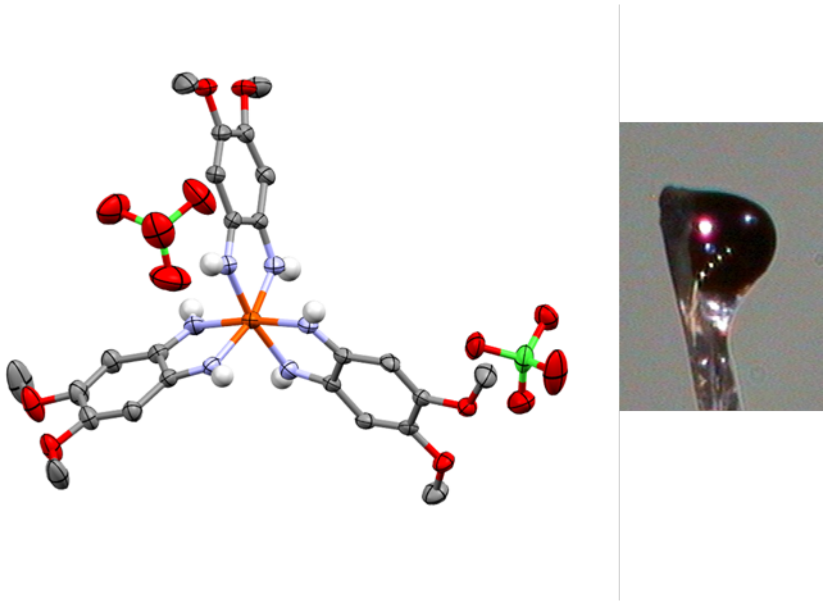

Figure S17. Displacement ellipsoid plot (50% probability level) of $^{\mathbf{MeO}}\mathbf{6}^{2+}$ at 110 (K). Selected H atoms were removed for clarity.

All reflection intensities were measured at 110(2) K using a SuperNova diffractometer (equipped with Atlas detector) with Cu *K*α radiation (λ = 1.54178 Å) under the program CrysAlisPro (Version CrysAlisPro 1.171.42.49, Rigaku OD, 2022). The same program was used to refine the cell dimensions and for data reduction. The structure was solved with the program SHELXS-2018/3 (Sheldrick, 2018) and was refined on F^2 with SHELXL-2018/3 (Sheldrick, 2018). Analytical numeric absorption correction using a multifaceted crystal model was applied using CrysAlisPro. The temperature of the data collection was controlled using the system Cryojet (manufactured by Oxford Instruments). The H atoms were placed at calculated positions (unless otherwise specified) using the instructions AFIX 43 or AFIX 137 with isotropic displacement parameters having values 1.2 or 1.5 U_{eq} of the attached C atoms. The H atoms attached to N1X and N2X (X = A-C) were found from difference Fourier maps, and their coordinates were refined pseudofreely using the DFIX instruction in order to keep the N–H bond distances within an acceptable range.

The structure is partly disordered. One ClO_4^- counterion is disordered over three orientations, and the sum of the three occupancy factors (0.520(3), 0.212(3), 0.268(3)) were constrained to be 1 using the SUMP instruction. The asymmetric unit also contains some amount of partially occupied and disordered lattice solvent molecules (MeCN), and their contribution was removed from the final refinement using the SQUEEZE procedure in Platon (Spek, 2009).

Table S6. Crystallographic data for $^{MeO}\mathbf{6}^{2+}$.

Crystal data	
Chemical formula	$C_{24}H_{30}FeN_6O_6 \cdot 2(ClO_4)$
M_r	753.29
Crystal system, space group	Monoclinic, $P2_1/c$
Temperature (K)	110
a, *b*, *c* (Å)	14.9428 (4), 14.8679 (4), 16.6635 (5)
β (°)	95.999 (3)
V (Å^3)	3681.82 (18)
Z	4
Radiation type	Cu $K\alpha$
μ (mm^{-1})	5.21
Crystal size (mm)	0.15 × 0.14 × 0.08
Data collection	
Diffractometer	SuperNova, Dual, Cu at zero, Atlas
Absorption correction	Analytical<br>*CrysAlis PRO* 1.171.42.49 (Rigaku Oxford Diffraction, 2022) Analytical numeric absorption correction using a multifaceted crystal model based on expressions derived by R.C. Clark & J.S. Reid. (Clark, R. C. & Reid, J. S. (1995). Acta Cryst. A51, 887-897) Empirical absorption correction using spherical harmonics, implemented in SCALE3 ABSPACK scaling algorithm.
T_{min}, T_{max}	0.574, 0.702
No. of measured, independent and observed [$I > 2\sigma(I)$] reflections	45876, 10034, 5798
R_{int}	0.062
$(\sin \theta/\lambda)_{max}$ (Å^{-1})	0.616
Refinement	
$R[F^2 > 2\sigma(F^2)]$, $wR(F^2)$, S	0.041, 0.084, 0.75
No. of reflections	10034
No. of parameters	542
No. of restraints	469
H-atom treatment	H atoms treated by a mixture of independent and constrained refinement
$\Delta\rho_{max}$, $\Delta\rho_{min}$ (e Å^{-3})	0.58, -0.33

3.10. $^{MeO}8H_2^{2+}$

In a N_2 filled glovebox, 4,5-dimethoxyl-1,2-phenylenediamine (505 mg, 3 mmol) was charged in a 30 mL vial and dissolved in 12 mL CH_3CN, then $[Fe(H_2O)_6](ClO_4)_2$ (360 mg, 1 mmol) was added as solid. Then the pale brown solution was transferred out of the glovebox, reacting with oxygen in air. The color of the solution rapidly turned navy blue in 10 seconds, then slowly turned purple in 1 hour. During the reaction, NMR was taken to monitor the full formation of $^{MeO}8H_2^{2+}$. Upon completion (deep purple solution), the reaction was quenched by removal of the solvent under vacuum to obtain the crude product (90% yield, usually the crude contains ~5% $^{MeO}6^{2+}$ due to over-oxidation). In a N_2-filled glovebox, the crude product was crystallized by layering Et_2O on top of a CH_3CN solution of $^{MeO}8H_2^{2+}$ to afford dark crystals suitable for SC-XRD character-ization.

^{1}H-NMR (CD_3CN-d_3): 3.79 (s, 6H, O-C**H**$_3$), 3.83 (s, 6H, O-C**H**$_3$), 3.92 (s, 6H, O-C**H**$_3$), 3.97 (d, 2H, N**H**$_2$), 4.97 (d, 2H, N**H**$_2$), 6.36 (s, 2H, Ph-**H**), 6.73 (s, 2H, Ph-**H**), 6.81 (s, 2H, Ph-**H**), 9.83 (s, 2H, N**H**), 11.48 (s, 2H, N**H**).

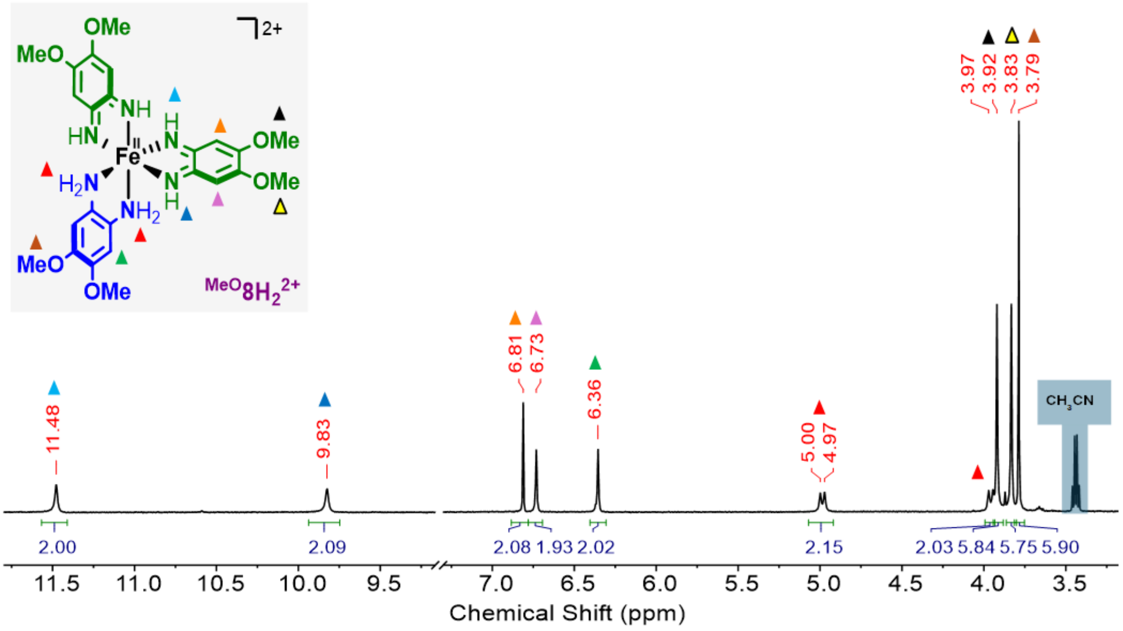

Figure S18. ^{1}H-NMR spectrum of $^{MeO}8H_2^{2+}$ in CD_3CN.

Elemental analysis: Chemical Formula: ($C_{24}H_{32}FeN_6O_6Cl_2O_8$ x$1.5H_2O$). calc: C (36.85%); H (4.51%); N (10.70%). exp: C (36.65%); H (4.12%); N (10.25%).

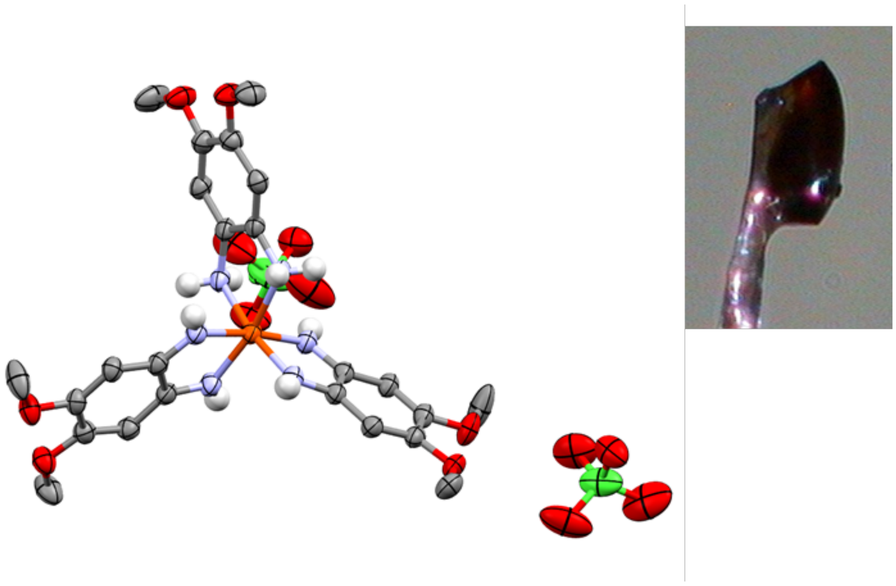

Figure S19. Displacement ellipsoid plot (50% probability level) of ^{MeO}**8H$_2^{2+}$** at 110 (K). Selected H atoms were removed for clarity.

All reflection intensities were measured at 110(2) K using a SuperNova diffractometer (equipped with Atlas detector) with Cu *K*α radiation (λ = 1.54178 Å) under the program CrysAlisPro (Version CrysAlisPro 1.171.42.49, Rigaku OD, 2022). The same program was used to refine the cell dimensions and for data reduction. The structure was solved with the program SHELXS-2018/3 (Sheldrick, 2018) and was refined on F^2 with SHELXL-2018/3 (Sheldrick, 2018). Analytical numeric absorption correction using a multifaceted crystal model was applied using CrysAlisPro. The temperature of the data collection was controlled using the system Cryojet (manufactured by Oxford Instruments). The H atoms were placed at calculated positions (unless otherwise specified) using the instructions AFIX 43 or AFIX 137 with isotropic displacement parameters having values 1.2 or 1.5 U_{eq} of the attached C atoms. The H atoms attached to N1X and N2X (X = A-C) were found from difference Fourier maps, and their coordinates were refined pseudofreely using the DFIX instruction in order to keep the N–H bond distances within an acceptable range.

The structure is partly disordered. The two ClO_4^- counterions are disordered over two orientations, and the occupancy factors of the major components of the disorder refine to 0.538(8) and 0.799(3). The asymmetric unit also contains some amount of very disordered lattice solvent molecule (MeCN), and their contribution was removed from the final refinement using the SQUEEZE procedure in Platon (Spek, 2009).

Table S7. Crystallographic data for ^{MeO}**8H$_2$$^{2+}$**.

Crystal data	
Chemical formula	$C_{24}H_{32}FeN_6O_6 \cdot 2(ClO_4)$
M_r	755.30
Crystal system, space group	Triclinic, *P*-1
Temperature (K)	110
a, *b*, *c* (Å)	11.7193 (3), 14.3141 (3), 15.0715 (3)
α, β, γ (°)	113.329 (2), 110.556 (2), 90.0749 (17)
V (Å^3)	2144.59 (9)
Z	2
Radiation type	Cu *K*α
μ (mm^{-1})	4.47
Crystal size (mm)	0.23 × 0.12 × 0.11
Data collection	
Diffractometer	SuperNova, Dual, Cu at zero, Atlas
Absorption correction	Analytical<br>*CrysAlis PRO* 1.171.42.49 (Rigaku Oxford Diffraction, 2022) Analytical numeric absorption correction using a multifaceted crystal model based on expressions derived by R.C. Clark & J.S. Reid. (Clark, R. C. & Reid, J. S. (1995). Acta Cryst. A51, 887-897) Empirical absorption correction using spherical harmonics, implemented in SCALE3 ABSPACK scaling algorithm.
T_{min}, T_{max}	0.498, 0.677
No. of measured, independent and observed [$I > 2\sigma(I)$] reflections	29025, 8378, 7462
R_{int}	0.031
$(\sin \theta/\lambda)_{max}$ (Å^{-1})	0.616
Refinement	
$R[F^2 > 2\sigma(F^2)]$, $wR(F^2)$, S	0.051, 0.148, 1.08
No. of reflections	8378
No. of parameters	546
No. of restraints	298
H-atom treatment	H atoms treated by a mixture of independent and constrained refinement
$\Delta\rho_{max}$, $\Delta\rho_{min}$ (e Å^{-3})	0.49, -0.59

3.11. $^{MeO}12H_6^{2+}$

In a N_2 filled glovebox, 4,5-dimethoxyl-1,2-phenylenediamine (252 mg, 1.5 mmol) was charged in a 30 mL vial and dissolved in 5 mL THF, then $[Fe(H_2O)_6](ClO_4)_2$ (180 mg, 0.5 mmol) was added as solid. White precipitate started forming upon addition of Fe source. The reaction was kept stirring for 30 minutes, after which the solvent was removed by filtration. The obtained white solid was dried in vacuum and then was crystallized by layering Et_2O on top of a CH_3CN solution to afford clear crystals suitable for SC-XRD characterization. After removal of the solvent, the product was washed with Et_2O (5 mL x 2) and dried in vacuum to afford colorless crystals in 80% yield.

^{1}H-NMR (CD_3CN): 6.12 (s, 18H, O-C**H**$_3$), 14.33 (bs, 12H, N**H**$_2$), 17.29 (bs, 6H, Ph-**H**).

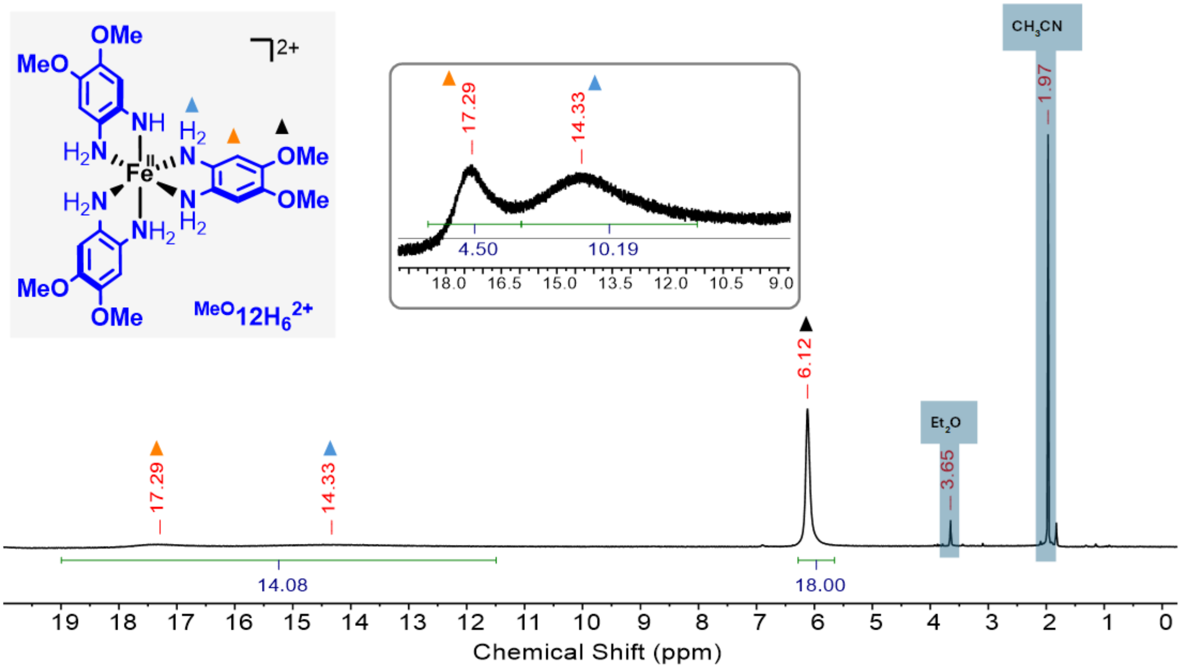

Figure S20. ^{1}H-NMR spectrum of ^{MeO}**12H$_6^{2+}$** in CD_3CN. The inset shows the broad singlets corresponding to aromatic and alkyl protons in the paramagnetic complex. Note: magnetic susceptibility measurements were carried out using the Evans method. The effective magnetic moment measured, μ_{eff} = 5.6 μB, is consistent with an high-spin iron(II) complex (4 unpaired electrons).

Elemental analysis: Chemical Formula: ($C_{24}H_{36}FeN_6O_6Cl_2O_8$ x2.5CH_3CN x0.5H_2O). calc: C (39.62%); H (5.32%); N (13.86%). exp: C (39.11%); H (4.93%); N (14.28%).

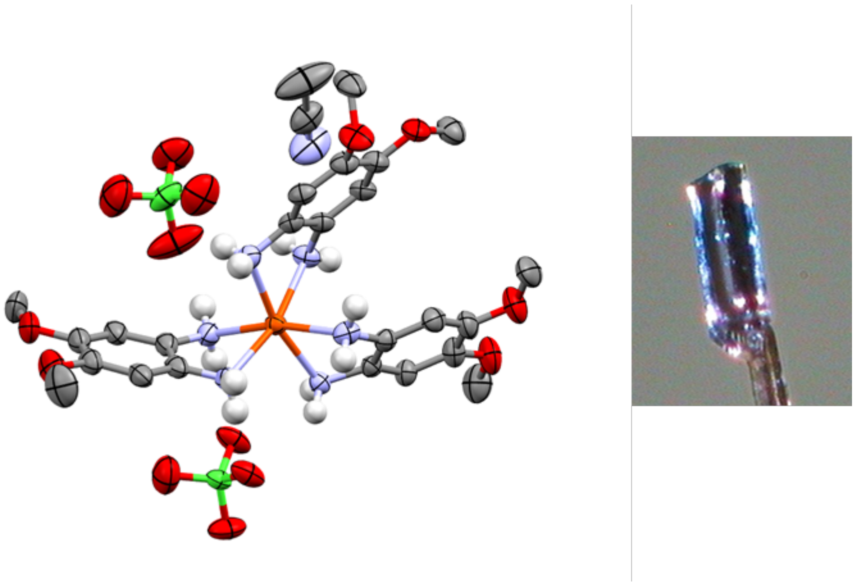

Figure S21. Displacement ellipsoid plot (50% probability level) of $^{\mathbf{MeO}}\mathbf{12H_6^{2+}}$ at 193 (K). Selected H atoms were removed for clarity.

All reflection intensities were measured at 193(2) K* using a SuperNova diffractometer (equipped with Atlas detector) with Cu *K*α radiation (λ = 1.54178 Å) under the program CrysAlisPro (Version CrysAlisPro 1.171.42.49, Rigaku OD, 2022). The same program was used to refine the cell dimensions and for data reduction. The structure was solved with the program SHELXS-2018/3 (Sheldrick, 2018) and was refined on F^2 with SHELXL-2018/3 (Sheldrick, 2018). Analytical numeric absorption correction using a multifaceted crystal model was applied using CrysAlisPro. The temperature of the data collection was controlled using the system Cryojet (manufactured by Oxford Instruments). The H atoms were placed at calculated positions (unless otherwise specified) using the instructions AFIX 43 or AFIX 137 with isotropic displacement parameters having values 1.2 or 1.5 U_{eq} of the attached C atoms. The H atoms attached to N1X and N2X (X = A-E) were found from difference Fourier maps, and their coordinates were refined pseudofreely using the DFIX instruction in order to keep the N–H bond distances within an acceptable range.

*Initially, data were collected at 110 K, but additional weak reflections were observed, suggesting a more complicated phase at low temperature. The temperature was raised to 193 K, and the diffraction pattern did not exhibit any extra unindexed reflections.

The structure is partly disordered. The moieties C1A→C8A, C1C→C8C and one of the two ClO_4^- counterions were found to be disordered over two orientations, and the occupancy factors of the major components of the disorder refine to 0.545(8), 0.528(16) and 0.825(5), respectively. The structure was refined as an inversion twin, and the Flack and Hooft parameters refine to 0.424(4) and 0.425(1), respectively.

Table S8. Crystallographic data for ^{MeO}**12H$_6^{2+}$**.

Crystal data	
Chemical formula	$C_{24}H_{36}FeN_6O_6 \cdot 2(ClO_4) \cdot C_2H_3N$
M_r	800.39
Crystal system, space group	Orthorhombic, $P2_12_12_1$
Temperature (K)	193
a, *b*, *c* (Å)	8.11251 (10), 14.1187 (2), 30.8626 (4)
V (Å^3)	3534.94 (8)
Z	4
Radiation type	Cu $K\alpha$
μ (mm^{-1})	5.47
Crystal size (mm)	0.36 × 0.12 × 0.07
Data collection	
Diffractometer	SuperNova, Dual, Cu at zero, Atlas
Absorption correction	Analytical<br>*CrysAlis PRO* 1.171.42.49 (Rigaku Oxford Diffraction, 2022) Analytical numeric absorption correction using a multifaceted crystal model based on expressions derived by R.C. Clark & J.S. Reid. (Clark, R. C. & Reid, J. S. (1995). Acta Cryst. A51, 887-897) Empirical absorption correction using spherical harmonics, implemented in SCALE3 ABSPACK scaling algorithm.
T_{min}, T_{max}	0.373, 0.750
No. of measured, independent and observed [$I > 2\sigma(I)$] reflections	23692, 6906, 6586
R_{int}	0.026
$(\sin \theta/\lambda)_{max}$ (Å^{-1})	0.616
Refinement	
$R[F^2 > 2\sigma(F^2)]$, $wR(F^2)$, S	0.029, 0.077, 1.03
No. of reflections	6906
No. of parameters	626
No. of restraints	966
H-atom treatment	H atoms treated by a mixture of independent and constrained refinement
$\Delta\rho_{max}$, $\Delta\rho_{min}$ (e Å^{-3})	0.28, -0.25
Absolute structure	Refined as an inversion twin.
Absolute structure parameter	0.424 (4)

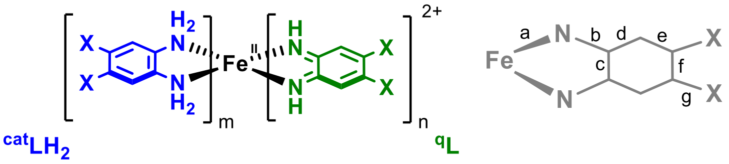

Table S9. Summary of all bond distances measured from the XRD data.[2]

X = H	Ligand	a	b	c	d	e	f
$^{H}\mathbf{6}^{2+}$	^{q}L	1.91	1.30	1.46	1.43	1.35	1.44
$^{H}\mathbf{8H_2}^{2+}$	^{q}L	1.90	1.31	1.45	1.43	1.36	1.44
	$^{cat}LH_2$	2.01	1.45	1.39	1.38	1.39	1.38
$^{H}\mathbf{12H_6}^{2+}$	$^{cat}LH_2$	2.22	1.44	1.40	1.38	1.38	1.38

X = Me	Ligand	a	b	c	d	e	f	g
$^{Me}\mathbf{6}^{2+}$	^{q}L	1.92	1.30	1.46	1.42	1.35	1.45	1.51
$^{Me}\mathbf{8H_2}^{2+}$	^{q}L	1.91	1.31	1.45	1.43	1.36	1.46	1.50
	$^{cat}LH_2$	2.01	1.45	1.37	1.38	1.39	1.39	1.51
$^{Me}\mathbf{12H_6}^{2+}$	$^{cat}LH_2$	2.22	1.44	1.38	1.38	1.38	1.39	1.51

X = MeO	Ligand	a	b	c	d	e	f	g
$^{MeO}\mathbf{6}^{2+}$	^{q}L	1.91	1.30	1.46	1.43	1.35	1.46	1.34
$^{MeO}\mathbf{8H_2}^{2+}$	^{q}L	1.91	1.32	1.45	1.42	1.36	1.46	1.35
	$^{cat}LH_2$	2.01	1.45	1.38	1.39	1.37	1.42	1.35
$^{MeO}\mathbf{12H_6}^{2+}$	$^{cat}LH_2$	2.22	1.44	1.38	1.39	1.38	1.39	1.36

4. Electrochemical and spectroscopic characterization

4.1. Cyclic voltammetry of $^{X}6^{2+}$

General procedure: 3 mL of a CH_3CN solution of the Fe complexes (1 mM) containing 0.1 M of $[NBu_4]PF_6$ was prepared in the glovebox and were transferred to an electrochemical cell outside the glovebox, which was purged with Ar for 5 minutes (note: a conventional three-electrode cell was used with a glassy carbon working electrode, an $Ag/AgNO_3$ (0.01 M) and platinum wire as the counter electrode). The potentials were measured with respect to the $Ag/AgNO_3$ reference electrode and converted to $Fc^{0/+}$ ($Fc^{0/+}$ potential measured under the same experimental conditions). Cyclic voltammograms were obtained at 100 mV/s scan rate. All electrochemical measurements were carried out under Ar atmosphere.

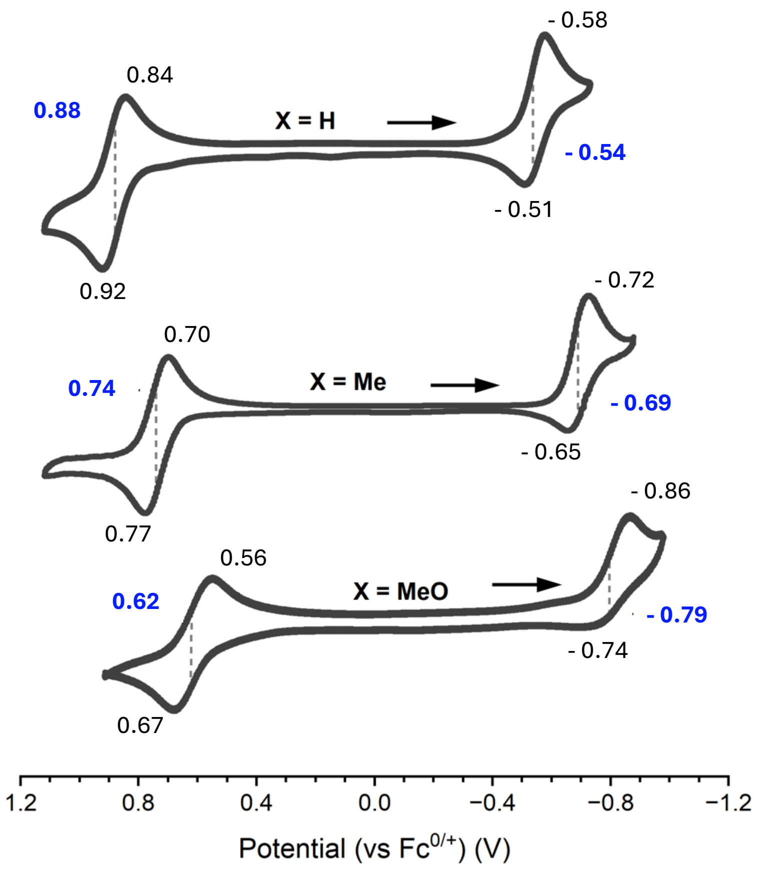

Figure S22. Cyclic voltammogram of $^{X}6^{2+}$ in CH_3CN. Note: the reduction of $^{MeO}6^{2+}$ was irreversible (a small anodic peak observed, with the cathodic peak at -0.86 V vs. $Fc^{0/+}$). For $^{MeO}6^{2+}$, the dotted line at -0.79V indicates the $E_{1/2}$ (potential that would be recorded if that redox event was reversible).

4.2 UV-vis spectroscopy

General procedure: In a typical experiment, 3 mL of a solution of Fe complexes (0.125 mM) in CH_3CN was prepared in a 10 mm path quartz cell that was capped with a rubber septum inside a N_2 purged glovebox. The quartz cell was purged with Ar while recording the spectra to prevent arial oxidation of the samples.

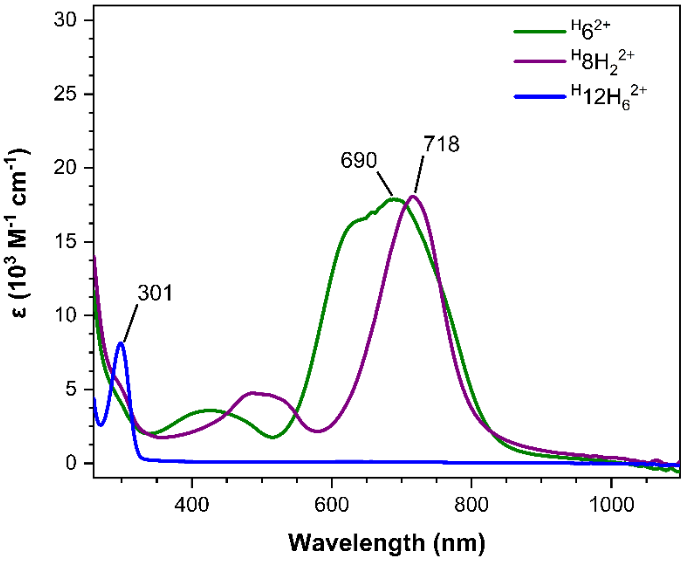

Figure S23. UV-vis characterization of the H-substituted complexes in CH_3CN.

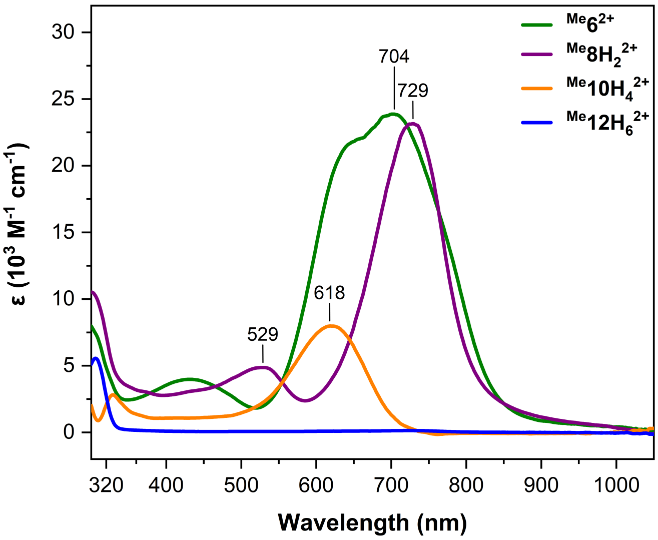

Figure S24. UV-vis characterization of the Me-substituted complexes in CH_3CN.

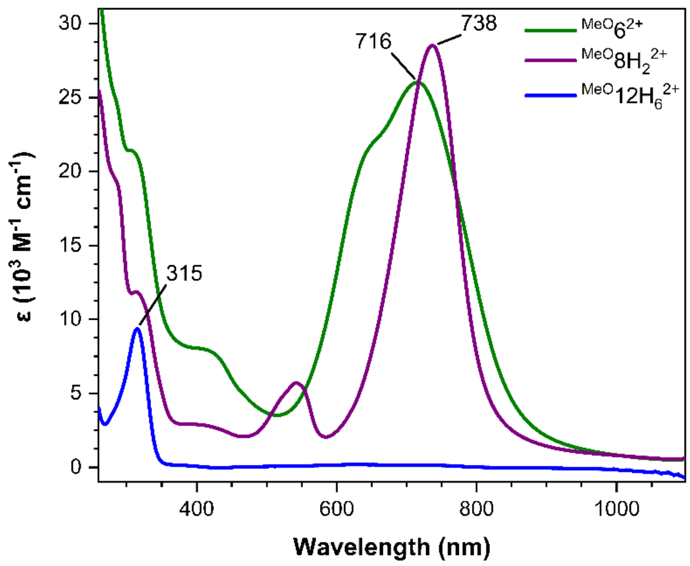

Figure S25. UV-vis characterization of the MeO-substituted complexes in CH_3CN.

4.3 Mössbauer Spectroscopy

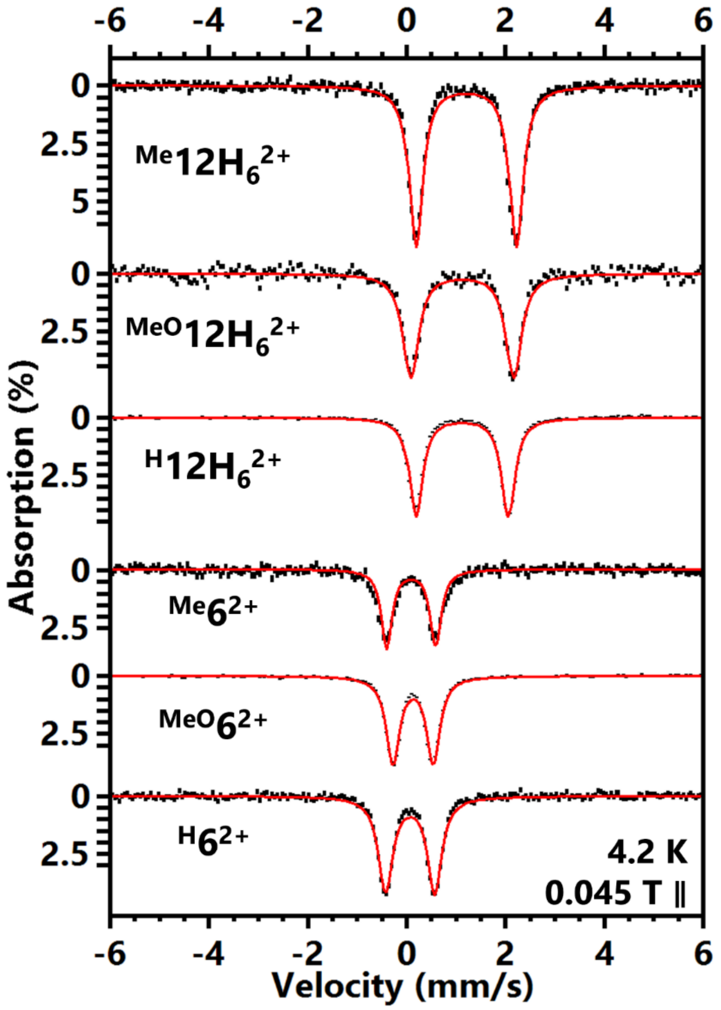

Figure S26. ^{57}Fe Mössbauer spectra of ^{X}**12H$_6$$^{2+}$** and ^{X}**6^{2+}** (X = Me, MeO and H) at 4.2 K, and an external field of 0.045 T parallel to the γ-radiation. Black bars represent experimental uncertainties, while rede lines represent the simulations. Mössbauer parameters (in the form [δ,|ΔE_Q|] in mm/s): [1.21, 2.01] for ^{Me}**12H$_6$$^{2+}$**, [1.12, 2.09] for ^{MeO}**12H$_6$$^{2+}$**, [1.13, 1.86] for ^{H}**12H$_6$$^{2+}$**, [0.09, 1.00] for ^{Me}**6^{2+}**, [0.13, 0.80] for ^{MeO}**6^{2+}**, and [0.08, 1.00] for ^{H}**6^{2+}**.

5. Oxidative deprotonation and reductive protonation of the Fe-based ECPBs.

NMR experiments: In the glovebox, stock solutions of appropriate concentrations of the complexes, 1,3,5-trimethoxybenzene (Internal Standard, Int. Std.) and relevant PCET reagents were prepared in CD_3CN. In a 7-inch, 5-mm o.d. NMR tube, a 0.9 mL CD_3CN solution containing desired concentrations of the complex (1-10 mM, unless otherwise noted) and Int. Std. (4-10 mM, unless otherwise noted) was recorded as the blank solution for each reactivity study. Following the blank spectrum, 0.1 mL (unless otherwise noted) of PCET reagents of desired concentration was added (5-30 mM, unless otherwise noted). Subsequently, the NMR tube was capped and sealed with Teflon tape to inhibit any unanticipated oxidation by O_2 during reaction time scale. Multiple NMR spectra over time revealed the reactivity of the systems under study.

The internal standard peak at 6.12 ppm (Ph-H, 3H) was used as reference for quantification of all components of the reactions. Complexes $^{\mathrm{X}}\mathbf{6}^{2+}$ were quantified using the integration values for singlet N-H peaks corresponding to 6 protons at around 11 ppm. Complexes $^{\mathrm{X}}\mathbf{8H_2}^{2+}$ were quantified using the average integrations of singlet N-H peaks corresponding to 2 protons each at the 10-13 ppm window and doublet N-H_2 peaks corresponding to 2 protons each at the 4-6 ppm window. Complexes $^{\mathrm{X}}\mathbf{10H_4}^{2+}$, which were observed *in-situ*, were quantified using the average integrations of a singlet N-H peak corresponding to 2 protons at around 11 ppm and a broad N-H_2 peak corresponding to 8 protons each at the 4-6 ppm window. Complexes $^{\mathrm{X}}\mathbf{12H_6}^{2+}$, which were inherently high-spin and paramagnetic, were challenging to quantify in the usual diamagnetic window. The broadness and chemical shifts of the signals would change depending on the concentration (being almost undetectable at < 2mM conc), and in general, yields erroneous quantification as seen in the decrease of mass balances. For quantification of $^{\mathrm{X}}\mathbf{12H_6}^{2+}$ at the end of some reactions, 30 mM bipyridine (bpy) was added to the reaction mixture. Bpy displaces the reduced ligands and ends up forming diamagnetic (low-spin) $Fe^{II}(bpy)_3$. This gives two handles to quantify the amounts of $^{\mathrm{X}}\mathbf{12H_6}^{2+}$ – concentrations of free ligand (= 3 x [$^{\mathrm{X}}\mathbf{12H_6}^{2+}$]) and $Fe^{II}(bpy)_3$ (= [$^{\mathrm{X}}\mathbf{12H_6}^{2+}$]).

The $6H^+/6e^-$ yield was based on the formation of oxidized products versus the initial concentration of the substrate. The ECPB mas balance was calculated from the total concentration of the resultant complexes and the of the PCET reagent product (if any) versus their initial concentration of the starting complex and starting PCET reagent (if any) respectively.

5.1 Reactivity towards O_2

5.1.1 $^{Me}12H_6^{2+}$ to $^{Me}6^{2+}$

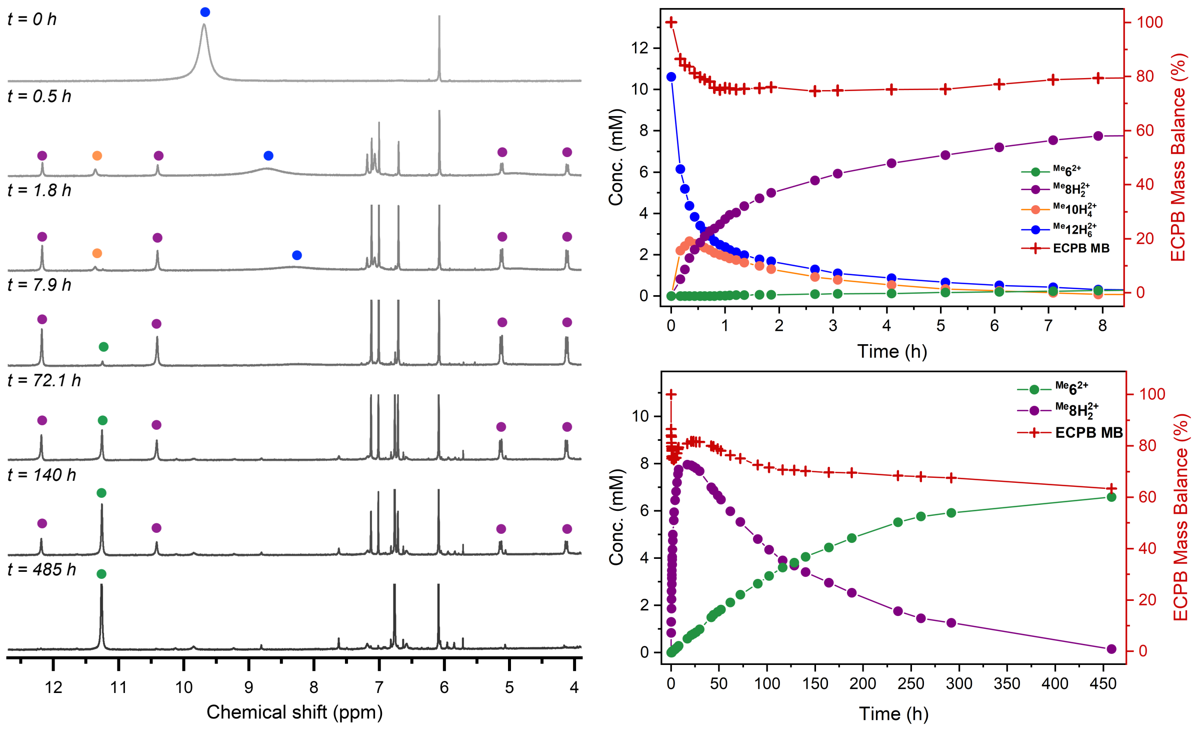

Figure S27. Reaction of ^{Me}**12H**$_6^{2+}$ with O_2 followed by NMR spectroscopy. The reaction of ^{Me}**12H**$_6^{2+}$ (blue dots) led to the stepwise formation of ^{Me}**10H**$_4^{2+}$, ^{Me}**8H**$_2^{2+}$ and ^{Me}**6**$^{2+}$ (orange, purple and green dots, respectively) with good ECPB mass balance (red crosses).

5.1.2 $^{H}12H_6^{2+}$ to $^{H}6^{2+}$

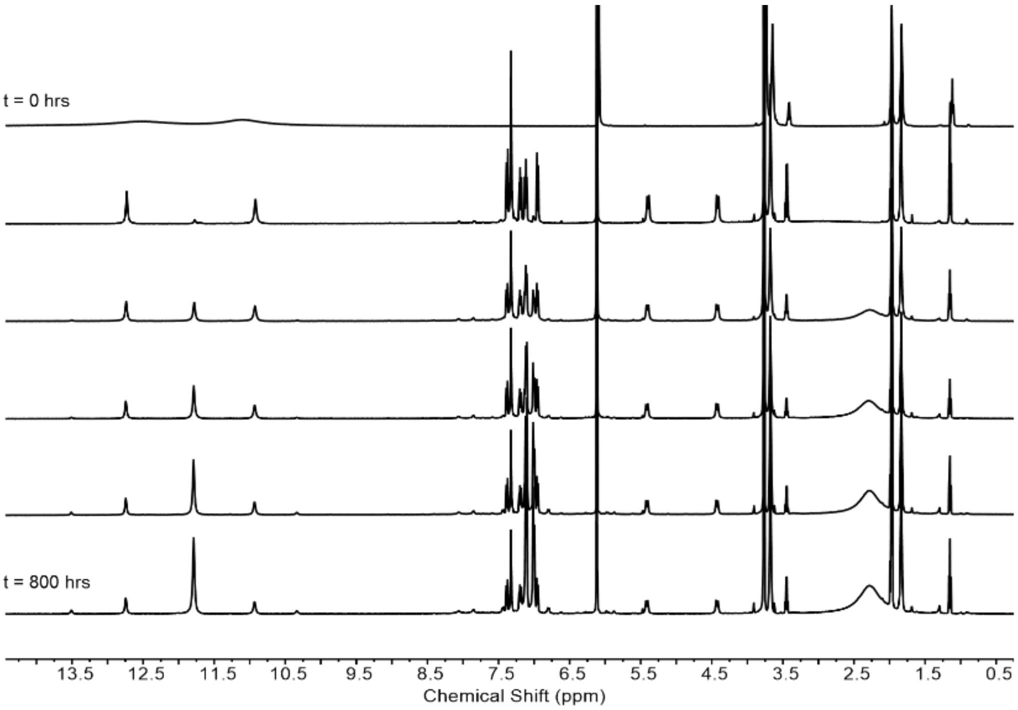

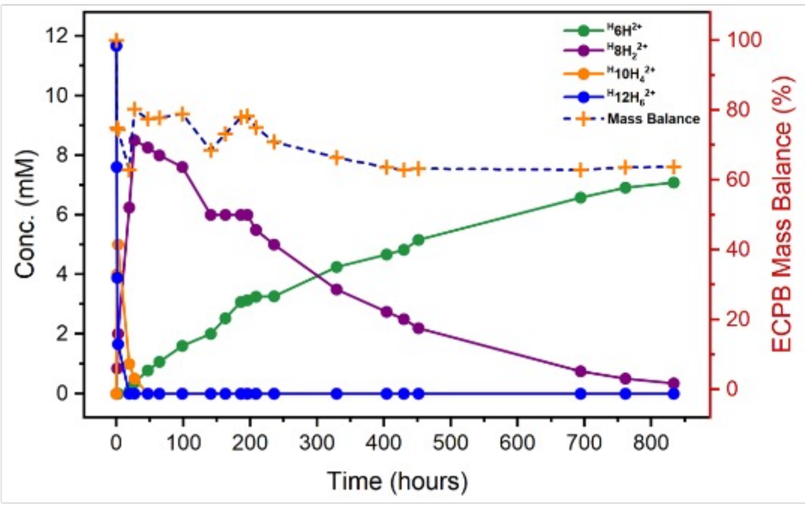

Figure S28. ^{1}H-NMR spectra in CD_3CN for the reaction between **$^{H}12H_6^{2+}$** and O_2 (top), and mass balance analysis (bottom). Note: In the aerobic oxidation of **$^{H}12H_6^{2+}$** we observe the formation of additional peaks in the 6.5 to 14 ppm region, which might arise from the oligomerization of opda.[2]

5.1.2 $^{MeO}12H_6^{2+}$ to $^{MeO}6^{2+}$

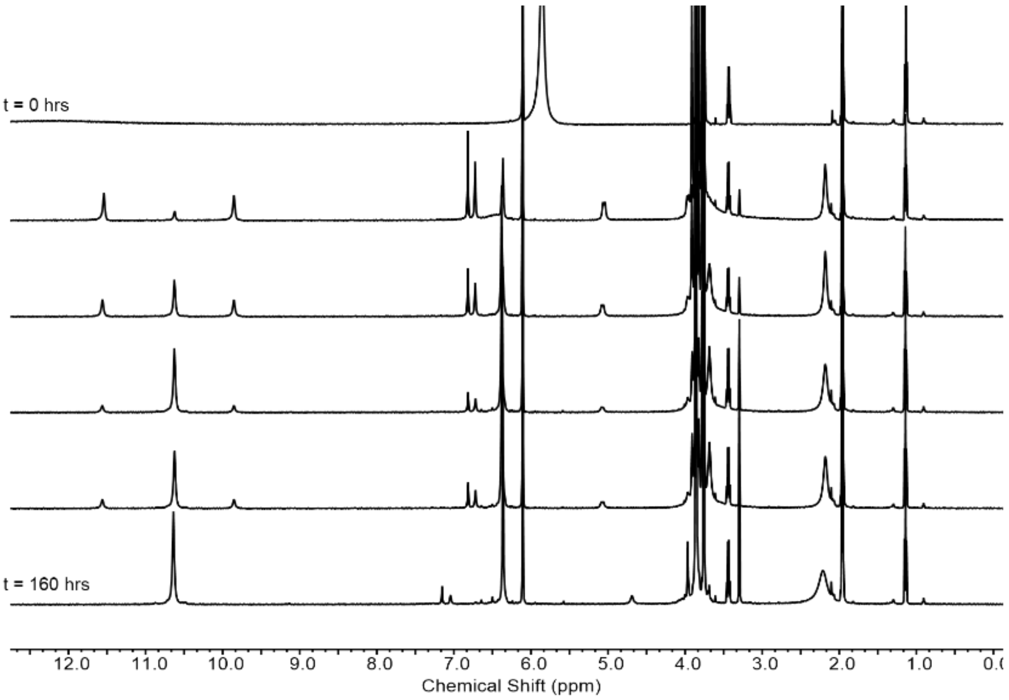

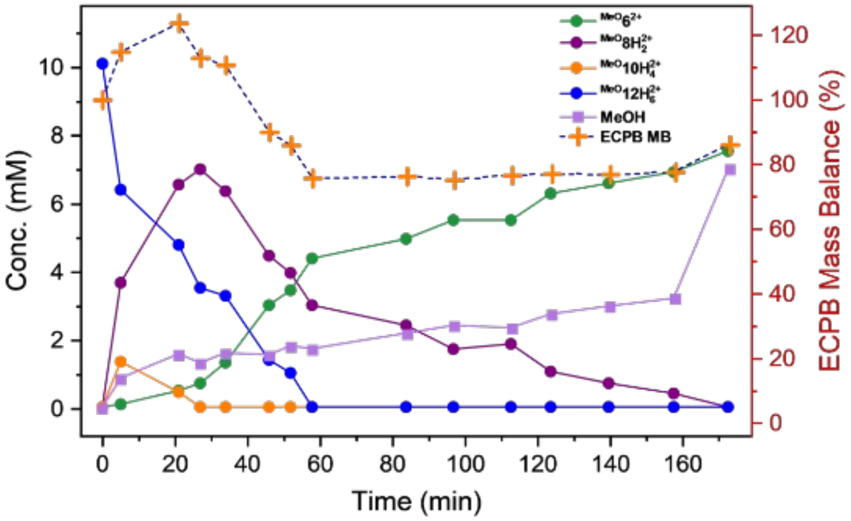

Figure S29: ^{1}H-NMR spectra in CD_3CN for the reaction between $^{MeO}\mathbf{12H_6}^{2+}$ and O_2 (top), and mass balance analysis (bottom). Note: In the aerobic oxidation of $^{MeO}\mathbf{12H_6}^{2+}$, we observed the formation of methanol derived from the degradation of the substituted opda ligand.[1]

5.2 Reactivity towards hydrazine

5.2.1 $^{Me}6^{2+}$ to $^{Me}12H_6^{2+}$

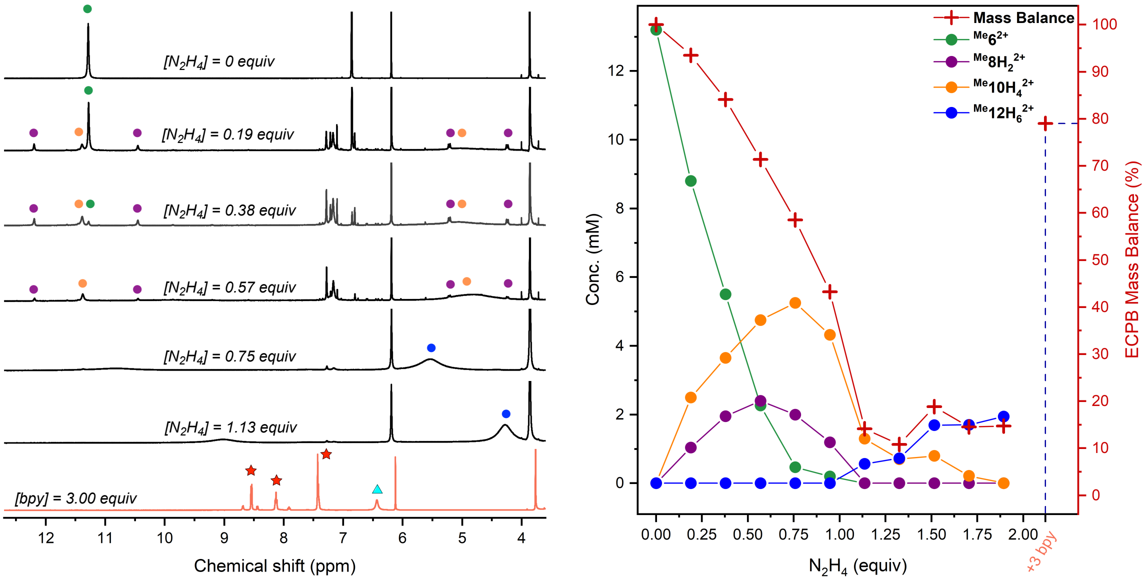

Figure S30. Reductive protonation of ^{Me}**6**$^{2+}$to ^{Me}**12H**$_6^{2+}$ using N_2H_4 followed by ^{1}H-NMR. Consecutive additions of substoichiometric amounts of N_2H_4 led to the stepwise reduction of ^{Me}**6**$^{2+}$ to ^{Me}**8H**$_2^{2+}$, ^{Me}**10H**$_4^{2+}$, and ^{Me}**12H**$_6^{2+}$. The peaks corresponding to the high-spin complex ^{Me}**12H**$_6^{2+}$ were shifted and broadened in the presence of hydrazine, which precluded their quantification. The addition of 3 equiv of bipyridine led to the formation of the low-spin $[Fe(bpy)_3]^{2+}$, which allowed to quantify the 4,5-Me_2-opda ligand produced in the reduction of ^{Me}**6**$^{2+}$ and determine the ECPB mass balance.

5.2.2 $^{H}6^{2+}$ to $^{H}12H_6^{2+}$

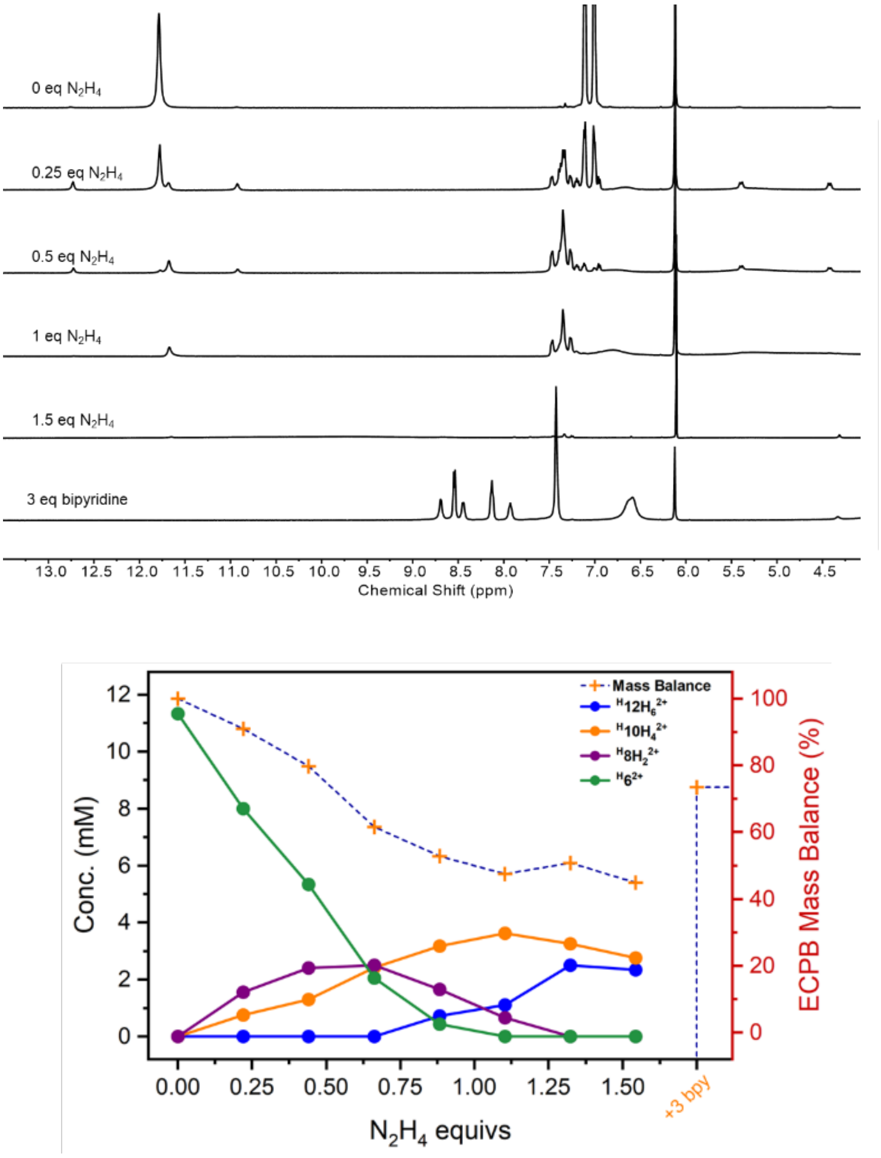

Figure S31. ^{1}H-NMR spectra in CD_3CN for the reaction between **$^{H}6^{2+}$** and N_2H_4 (top), and mass balance analysis (bottom).

5.2.3 $^{MeO}6^{2+}$ to $^{MeO}12H_6^{2+}$

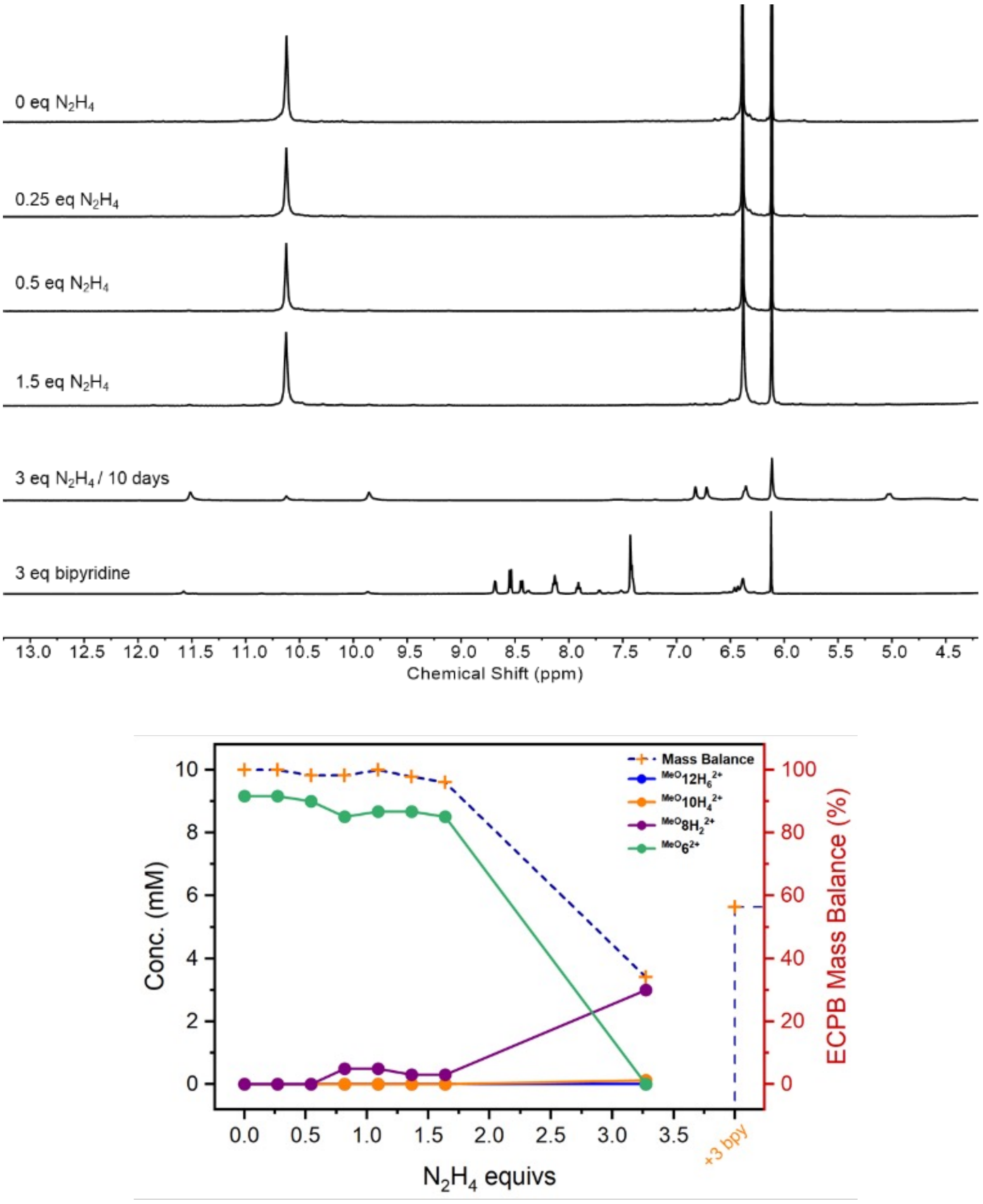

Figure S32. ^{1}H-NMR spectra in CD_3CN for the reaction between ^{MeO}**6**$^{2+}$ and N_2H_4 (top), and mass balance analysis (bottom). Note: additional equivalents and long reaction times were required to fully reduce ^{MeO}**6**$^{2+}$.

5.2.4 Addition of N_2H_4 to $^{Me}12H_6^{2+}$

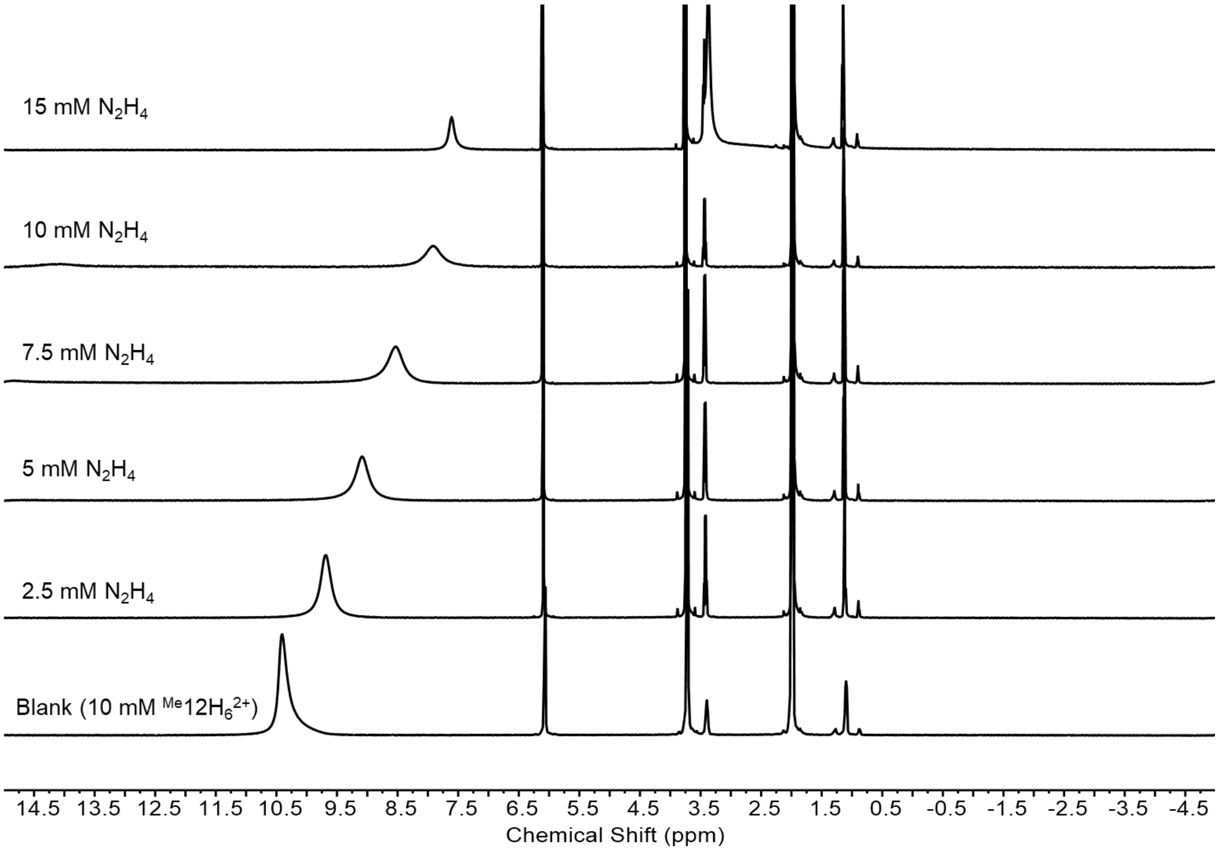

Figure S33. ^{1}H-NMR spectra in CD_3CN for the reaction between $^{Me}\mathbf{12H_6^{2+}}$ and N_2H_4. Note: Like in the reduction of $^{Me}\mathbf{6^{2+}}$ with N_2H_4, the addition of N_2H_4 to $^{Me}\mathbf{12H_6^{2+}}$ leads to shifting, broadening and decrease of the paramagnetic peaks.

5.2.5 Addition of bpy to $^{Me}6^{2+}$ and to $^{Me}8H_2^{2+}$

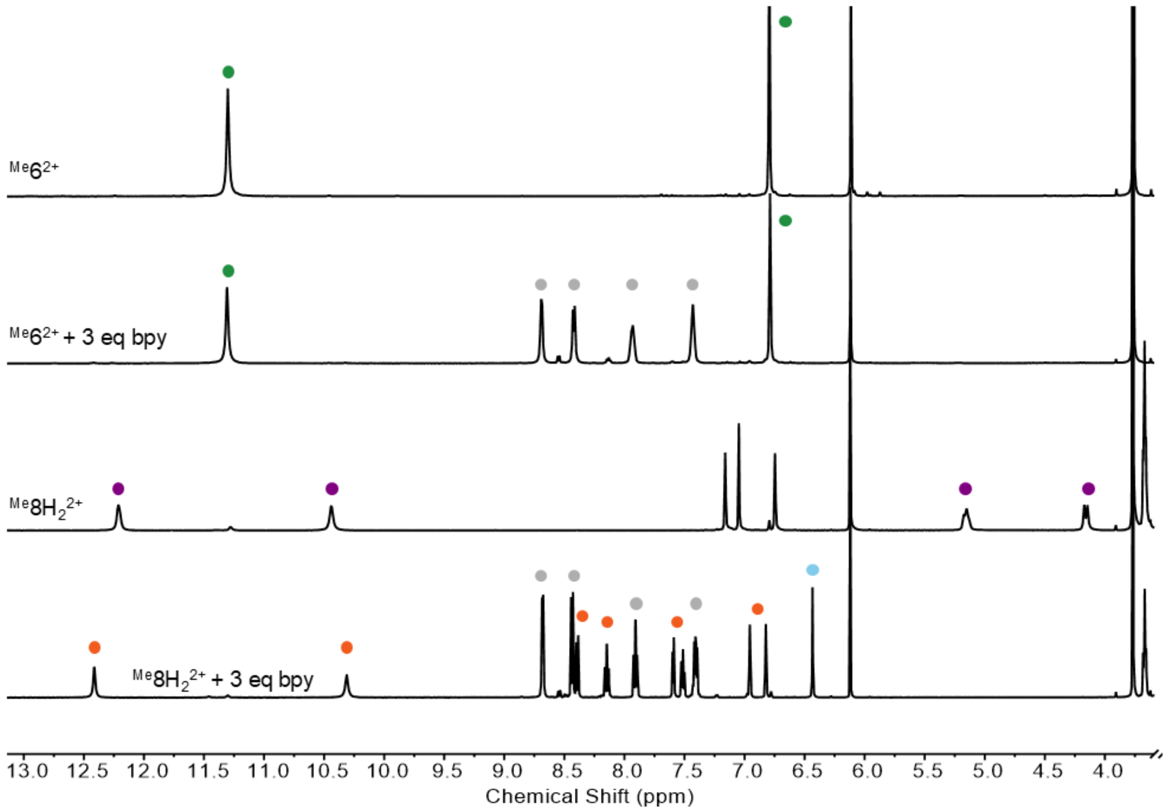

Figure S34. ^{1}H-NMR spectra in CD_3CN for the reaction between $^{Me}\mathbf{6}^{2+}$ and 3 equiv of bpy (top two spectra) and reaction between $^{Me}\mathbf{8H_2}^{2+}$ and 3 equiv of bpy (bottom two spectra). In the reaction between $^{Me}\mathbf{6}^{2+}$ and 3 equiv of bpy we did not observe any spectral changes. In the reaction between $^{Me}\mathbf{8H_2}^{2+}$ and 3 equiv of bpy we observed the formation of an heteroleptic complex $[Fe(^{q}L)_2(bpy)]^{2+}$ (orange dots) and with concomitant formation of 1 equiv of opda ligand (blue dots). Note: peaks corresponding to free bypiridine are observed in both reactions (grey dots).

6. Thermochemistry

6.1 Reactivity using PCET reagents

The reactions in this section were conducted by first taking a blank NMR of the complex ($^{X}\mathbf{6}^{2+}$, 1 mM) with an internal standard (1,3,5-trimethoxybenzene, 4 mM) in CD_3CN. Then various PCET reagents (10 mM) were added to the blank NMR sample and the changes were monitored over time. For complexes with $BDFE_{avg}$ close to a PCET reagent, we observed equilibration. For complexes with $BDFE_{avg}$ higher than the PCET reagent, formation of $^{X}\mathbf{12H_6}^{2+}$ and quinone, and the consumption of $^{X}\mathbf{6}^{2+}$ and hydroquinone was observed. For complexes with $BDFE_{avg}$ lower than the PCET reagent, there was no reaction.

Table S10. Summary of all reactions of $^{X}\mathbf{6}^{2+}$ complexes with different PCET reagents.

**PCET Substrate**	**BDFE (kcal/mol)**	$^{H}\mathbf{6}^{2+}$	$^{Me}\mathbf{6}^{2+}$	$^{MeO}\mathbf{6}^{2+}$
2,6-Cl_2-H_2Q	68.9	NR*	NR*	NR*
1,4-H_2Q	67.3	Equilibrium	NR*	NR*
2,6-Me_2-1,4-H_2Q	64.6	$^{H}\mathbf{12H_6}^{2+}$	Equilibrium	NR*
2,6-Me_2O-1,4-H_2Q	62.8	$^{H}\mathbf{12H_6}^{2+}$	$^{Me}\mathbf{12H_6}^{2+}$	Equilibrium
PhNHNHPh	60.9	$^{H}\mathbf{12H_6}^{2+}$	$^{Me}\mathbf{12H_6}^{2+}$	$^{MeO}\mathbf{12H_6}^{2+}$

NR* = No Reaction, there was no change in the NMR spectrum for 7 days.

6.1.1 $^{H}6^{2+}$ + PCET reagents:

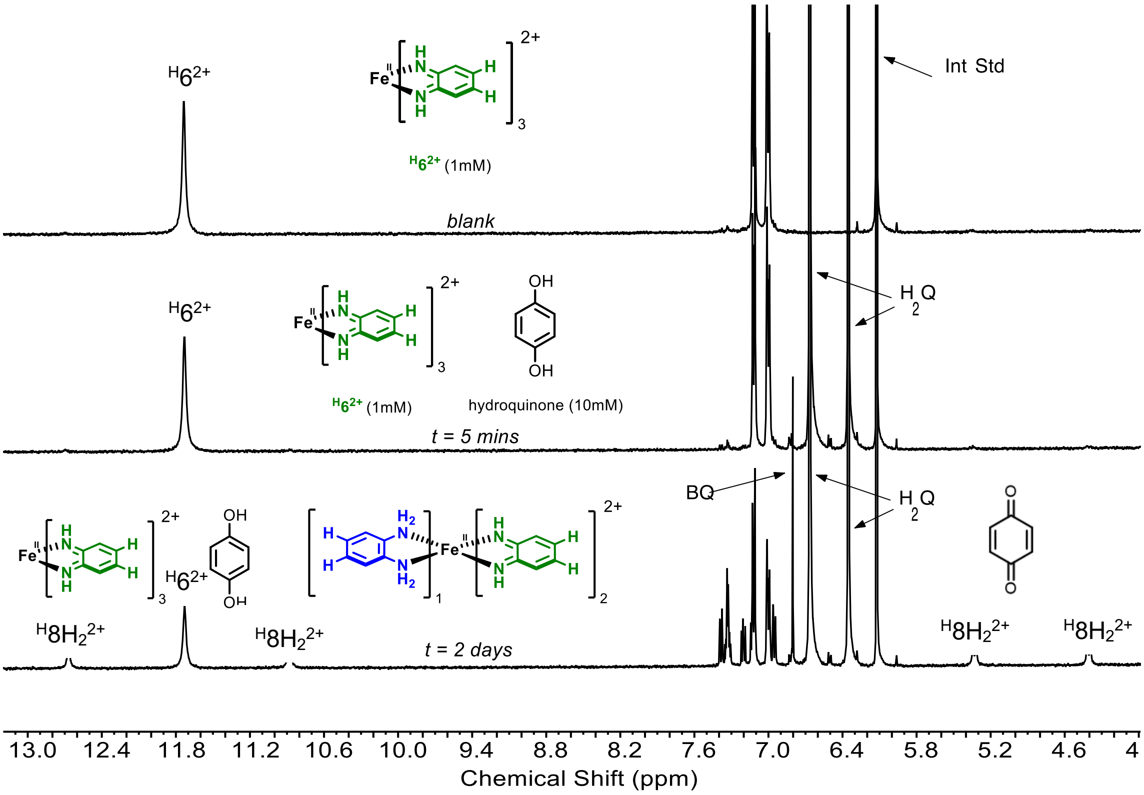

Figure S35. ^{1}H-NMR spectra in CD_3CN for the reaction: **$^{H}6^{2+}$** + 1,4-H_2Q**.**

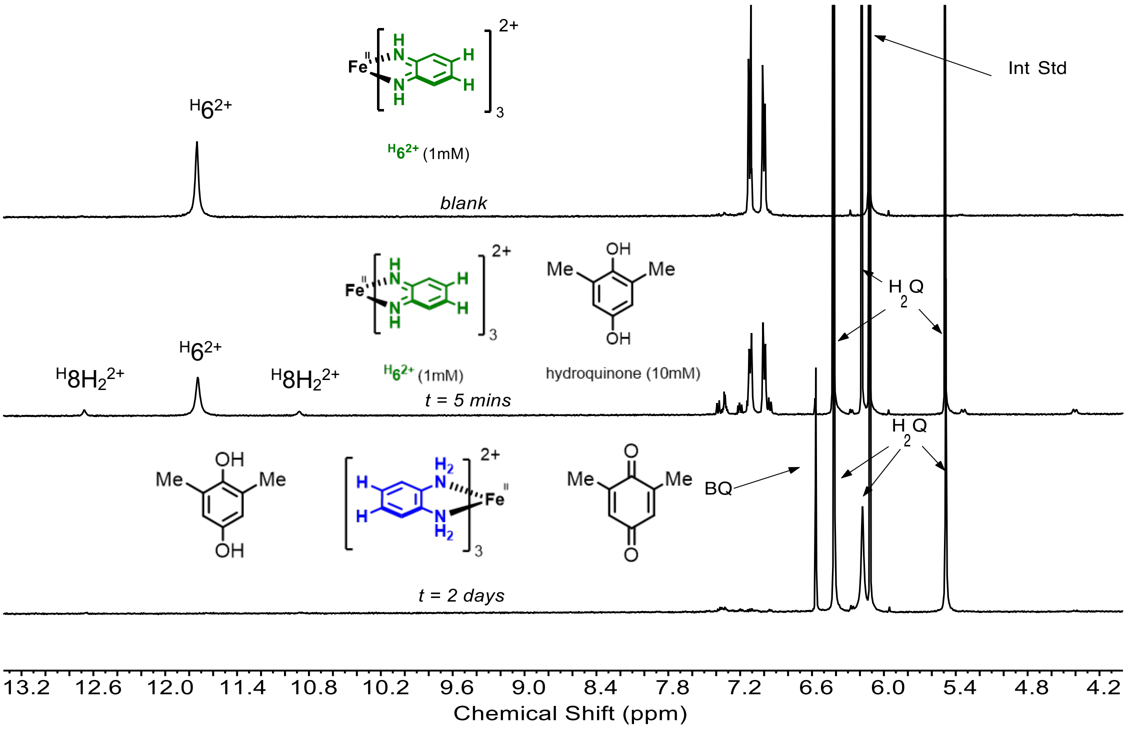

Figure S36. ^{1}H-NMR spectra in CD_3CN for the reaction: **$^{H}6^{2+}$** + 2,6-Me_2-1,4-H_2Q**.**

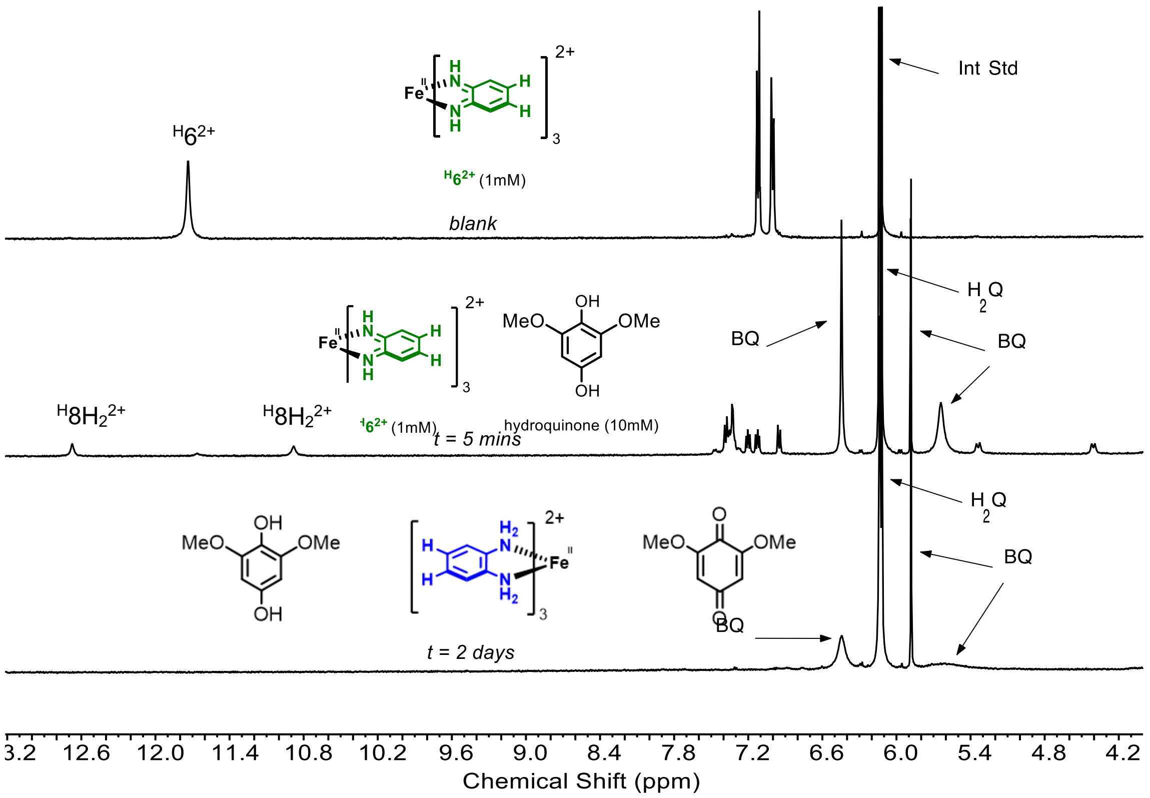

Figure S37. ^{1}H-NMR spectra in CD_3CN-d_3 for the reaction: $\mathbf{^{H}6^{2+}}$ + 2,6-$(MeO)_2$-1,4-H_2Q.

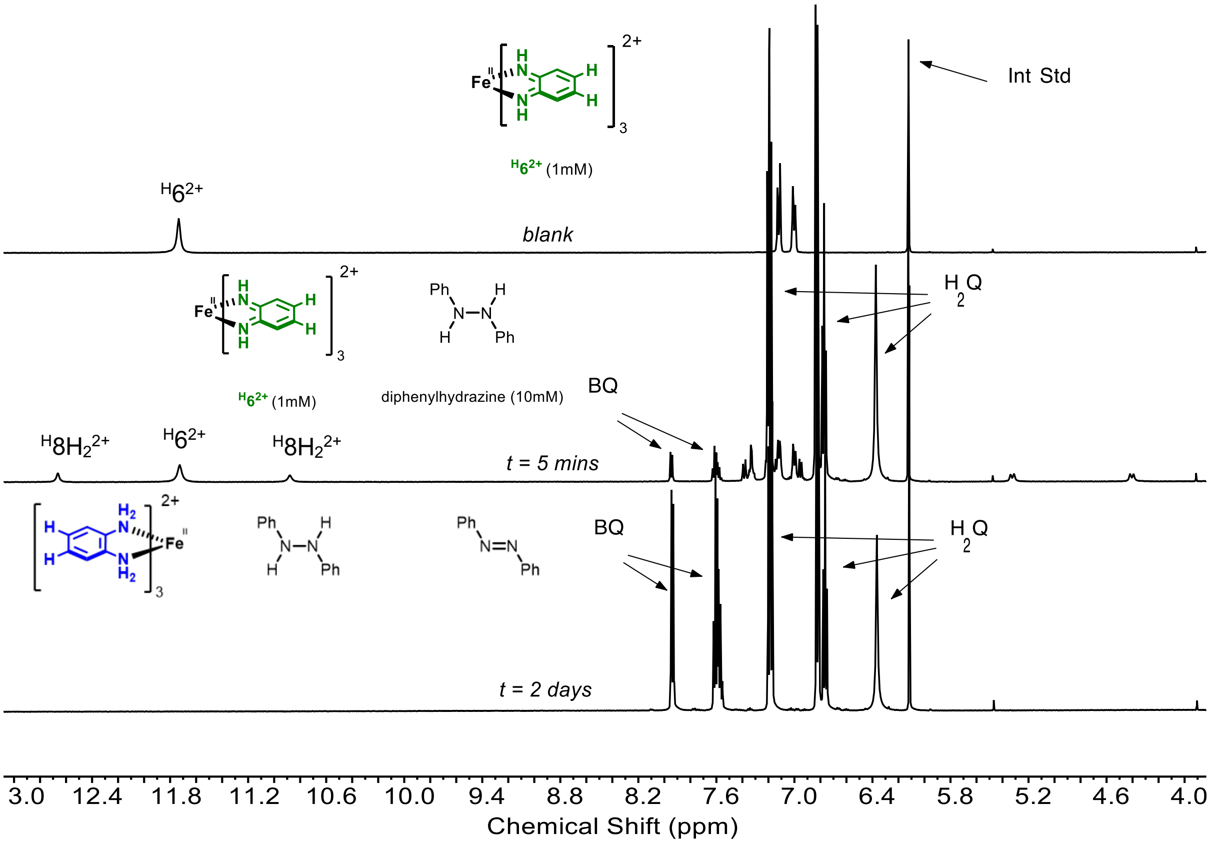

Figure S38. ^{1}H-NMR spectra in CD_3CN for the reaction: $\mathbf{^{H}6^{2+}}$ + DPH.

6.1.2 $^{Me}6^{2+}$ + PCET reagents:

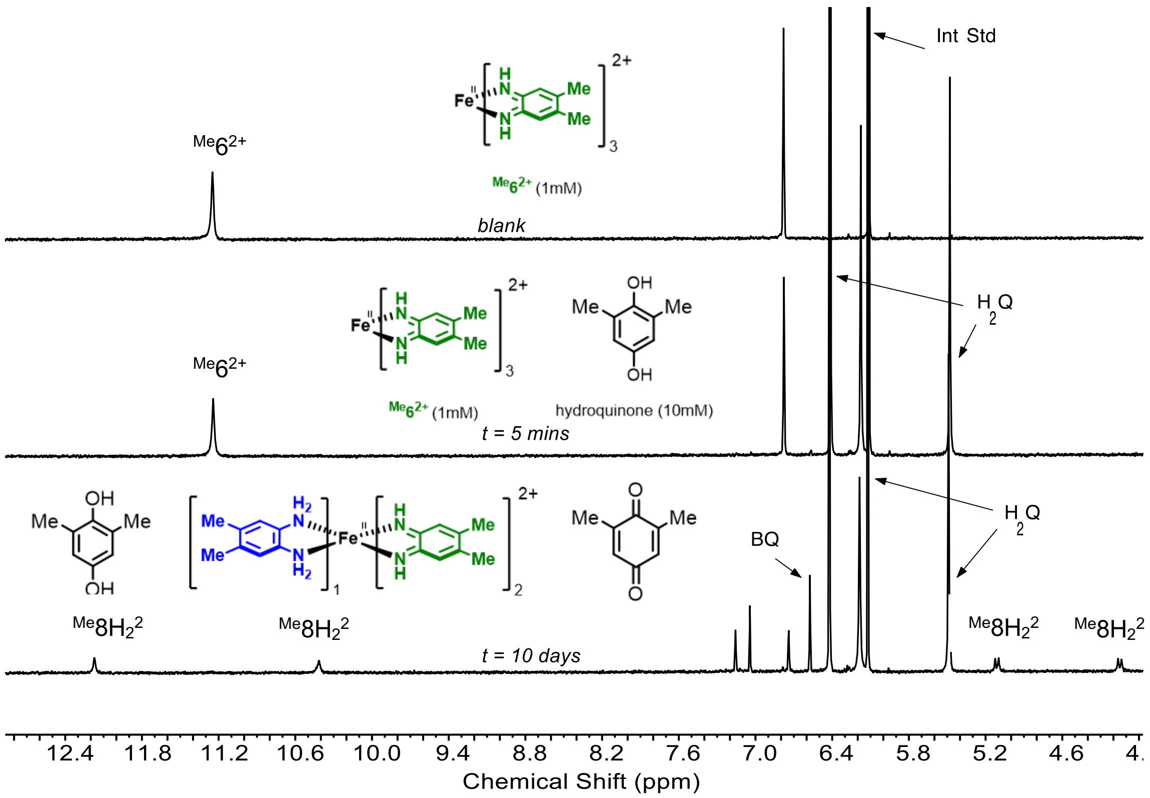

Figure S39. ^{1}H-NMR spectra in CD_3CN for the reaction: $^{Me}\mathbf{6}^{2+}$ + 2,6-Me_2-1,4-H_2Q.

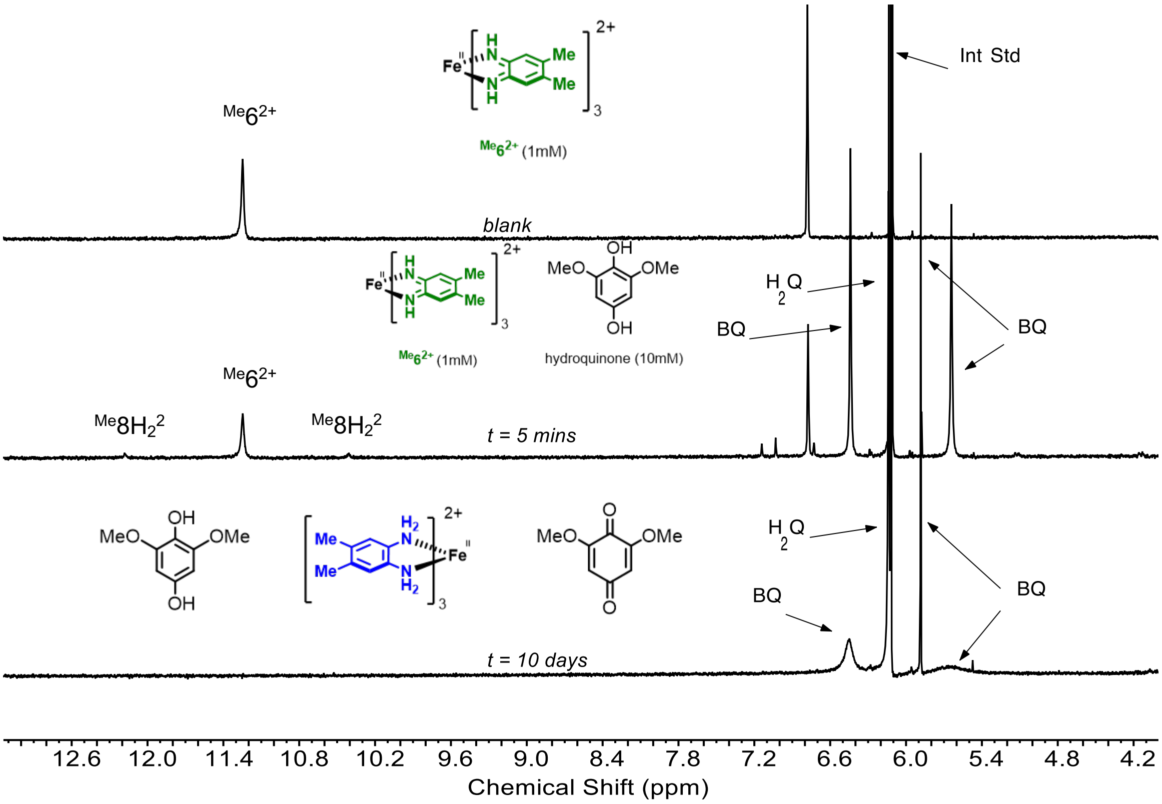

Figure S40. ^{1}H-NMR spectra in CD_3CN for the reaction: $^{Me}\mathbf{6}^{2+}$ + 2,6-$(MeO)_2$-1,4-H_2Q.

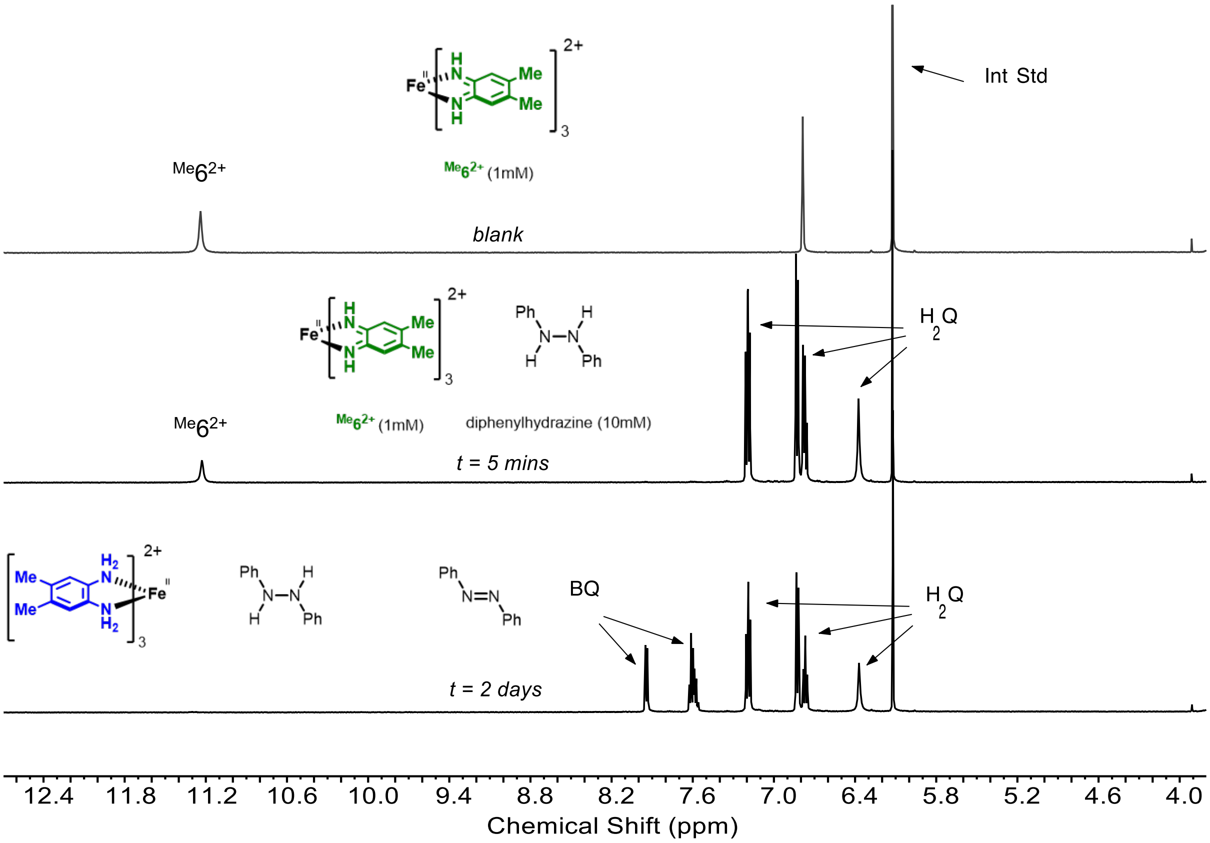

Figure S41. ^{1}H-NMR spectra in CD_3CN for the reaction: **$^{Me}6^{2+}$** + DPH.

6.1.3 $^{MeO}6^{2+}$ + PCET reagents:

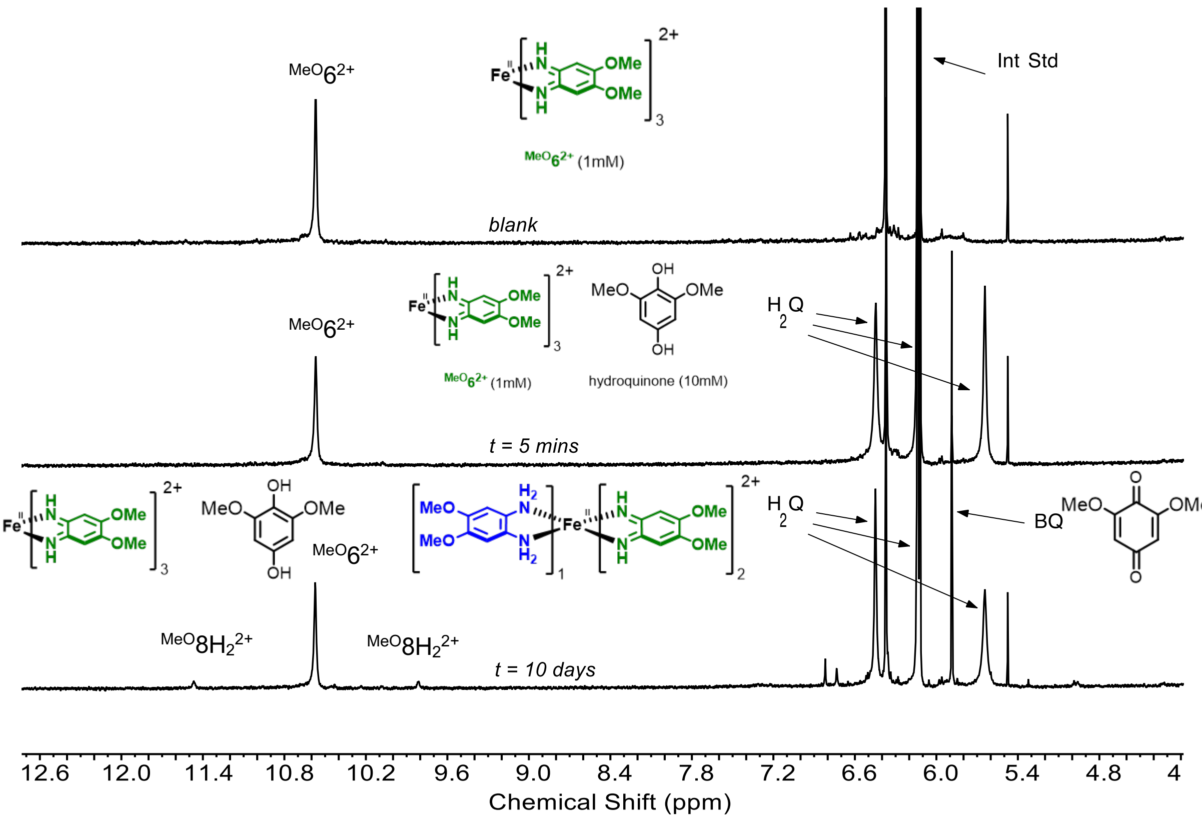

Figure S42. ^{1}H-NMR spectra in CD_3CN for the reaction: $^{MeO}\mathbf{6}^{2+}$ + 2,6-$(MeO)_2$-1,4-H_2Q.

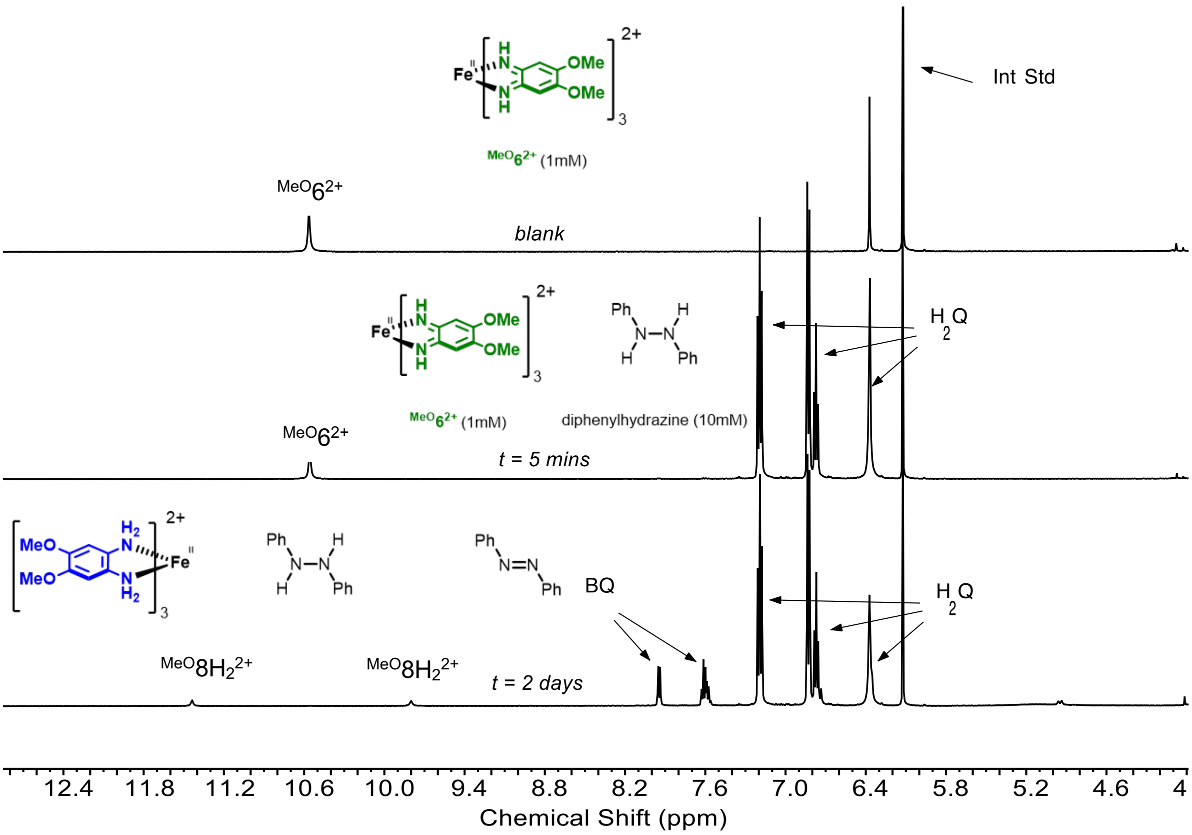

Figure S43. ^{1}H-NMR spectra in CD_3CN for the reaction: $^{MeO}\mathbf{6}^{2+}$ + DPH.

6.2 BDFE Calculations from comproportionation reactions

The comproportionation reactions were performed by preparing equimolar (10 mM) 1 mL solutions of $^{X}\mathbf{6}^{2+}$ and $^{X}\mathbf{12H_{12}}^{2+}$ in CD_3CN-d_3 inside a N_2 filled glovebox. These solutions were transferred into 7-inch, 5-mm o.d. NMR tube along of an internal standard (1,3,5-trimethoxybenzene, 10 mM). The concentration of all 4 species of the respective family of the ECPB was closely monitored over time using NMR to calculate equilibrium constants (K_{eq}). The K_{eq} values were then used to calculate the $BDFE_{avg}$. The set of all possible equilibration reactions and their corresponding K_{eq} are shown below.

Scheme S1. Thermochemistry of the ligand-exchange reactions involved in the $^{X}\mathbf{6}^{2+}$/$^{X}\mathbf{12H_6}^{2+}$ systems.

$$^{X}8H_2^{2+} \xrightarrow[BDFE_{avg1,2}]{} {}^{X}6^{2+} + 2\,H\bullet \qquad (1)$$

$$\Delta G^0_{react} = 2\,(BDFE_{avg1,2})$$

$$^{X}10H_4^{2+} \xrightarrow[BDFE_{avg3,4}]{} {}^{X}8H_2^{2+} + 2\,H\bullet \qquad (2)$$

$$\Delta G^0_{react} = 2\,(BDFE_{avg3,4})$$

$$^{X}12H_6^{2+} \xrightarrow[BDFE_{avg5,6}]{} {}^{X}10H_4^{2+} + 2\,H\bullet \qquad (3)$$

$$\Delta G^0_{react} = 2\,(BDFE_{avg5,6})$$

$$^{X}6^{2+} + {}^{X}12H_6^{2+} \underset{}{\overset{K_{eq1}}{\rightleftharpoons}} {}^{X}8H_2^{2+} + {}^{X}10H_4^{2+} \qquad (4)$$

$$\Delta G^0_{react} = -RT\ln K_{eq1}$$

$$\Delta G^0_{react} = -2\,(BDFE_{avg1,2} - BDFE_{avg5,6})$$

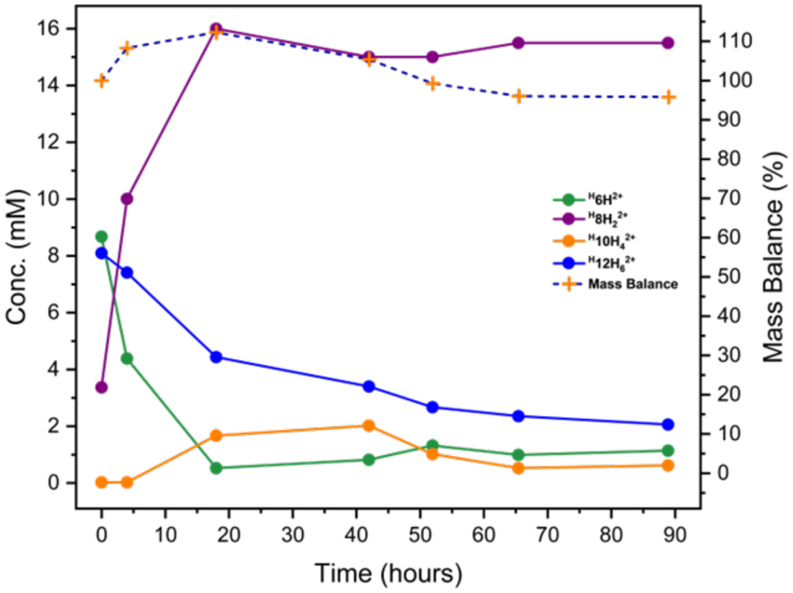

Figure S44. Mass balance analysis of the comproportionation reaction between ^{H}**6**$^{2+}$ (~10 mM) and ^{H}**12H**$_{6}$$^{2+}$ (~10 mM) Final concentrations: ^{H}**6**$^{2+}$**:** 0.23 mM, ^{H}**8H**$_{2}$$^{2+}$: 14.4 mM, ^{H}**10H**$_{4}$$^{2+}$: 4.0 mM, ^{H}**12**$_{6}$$^{2+}$: 2.75 mM.

Example of BDFEs calculation ($^{H}6^{2+}$/$^{H}12H_6^{2+}$ system):

$$^{X}\mathbf{6}^{2+} + {}^{X}\mathbf{12H_6}^{2+} \xrightleftharpoons{K_{eq1}} {}^{X}\mathbf{8H_2}^{2+} + {}^{X}\mathbf{10H_4}^{2+}$$

$$K_{eq1} = \frac{[^{X}8H_2{}^{2+}][^{X}10H_4{}^{2+}]}{[^{X}6^{2+}][^{X}12H_6{}^{2+}]} \qquad \Delta G°_1 = -RT\,lnK_{eq1} = 2\,(BDFE_{5,6} - BDFE_{1,2})$$

	$^{H}\mathbf{6}^{2+}$ (mM)	$^{H}\mathbf{8H_2}^{2+}$ (mM)	$^{H}\mathbf{10H_4}^{2+}$ (mM)	$^{H}\mathbf{12H_6}^{2+}$ (mM)	Total Fe (mM)	K_{eq1}
Initial	8.5	3.3	0.5	7.9	20.2	0.02457
Final	0.23	14.4	4	2.75	21.4	91.067

ΔG^0_1	$BDFE_{avg1,2}$ (OCP)	$BDFE_{avg3,4}$ (reactivity)	$BDFE_{avg5,6}$ (comproportionation)	$BDFE_{avg1,6}$
-2.671	66.2	~65	64.9	~65

Note: The $BDFE_{avg3,4}$ was estimated from the reactivity experiments, in which we found that complex $^{H}\mathbf{8H_2}^{2+}$ did not react with H_2Q ($BDFE_{avg}$ = 67.3 kcal/mol in CH_3CN) but $^{H}\mathbf{6}^{2+}$ did, indicating the $BDFE_{avg}$ of the $^{H}\mathbf{6}^{2+}$/$^{H}\mathbf{8H_2}^{2+}$ is higher than the $BDFE_{avg}$ of the $^{H}\mathbf{8H_2}^{2+}$/$^{H}\mathbf{10H_4}^{2+}$ couple (i.e., $BDFE_{avg1,2} > BDFE_{avg3,4}$). We also observed that $^{H}\mathbf{8H_2}^{2+}$ reacted with 2,6-Me_2-H_2Q ($BDFE_{avg}$ = 64.6 kcal/mol in CH_3CN), suggesting that the $BDFE_{avg}$ of the $^{H}\mathbf{8H_2}^{2+}$/$^{H}\mathbf{10H_4}^{2+}$ couple is higher than 64.6 kcal/mol. With all these data, we can estimate the $BDFE_{avg}$ of the $^{H}\mathbf{8H_2}^{2+}$/$^{H}\mathbf{10H_4}^{2+}$ couple ($BDFE_{avg3,4}$ ~ 65 kcal/mol) as well as the overall $BDFE_{avg}$ ($BDFE_{avg1,6}$ ~ 65 kcal/mol).

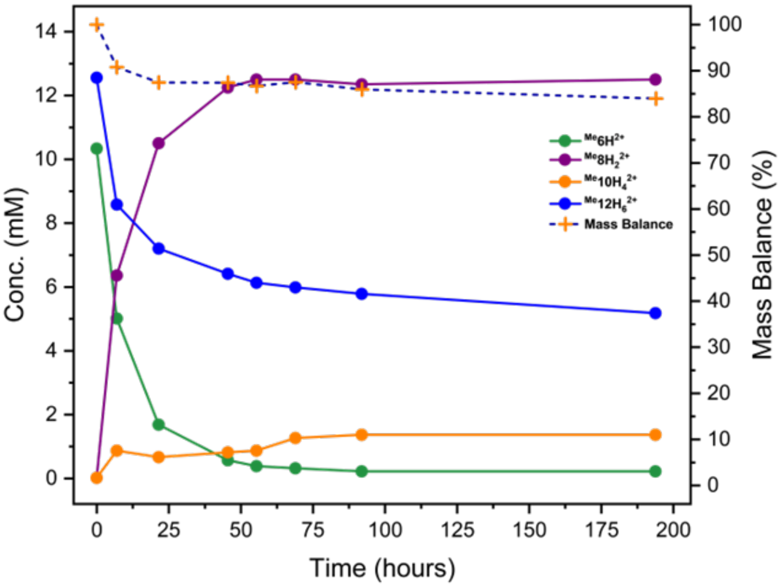

Figure S45. Mass balance analysis of the comproportionation reaction between $^{Me}\mathbf{6}^{2+}$ (~10 mM) and $^{Me}\mathbf{12H_6}^{2+}$ (~10 mM) Final concentrations: $^{Me}\mathbf{6}^{2+}$**:** 0.2 mM, $^{Me}\mathbf{8H_2}^{2+}$: 12.5 mM, $^{Me}\mathbf{10H_4}^{2+}$: 1.4 mM, $^{Me}\mathbf{12_6}^{2+}$: 5.2 mM.

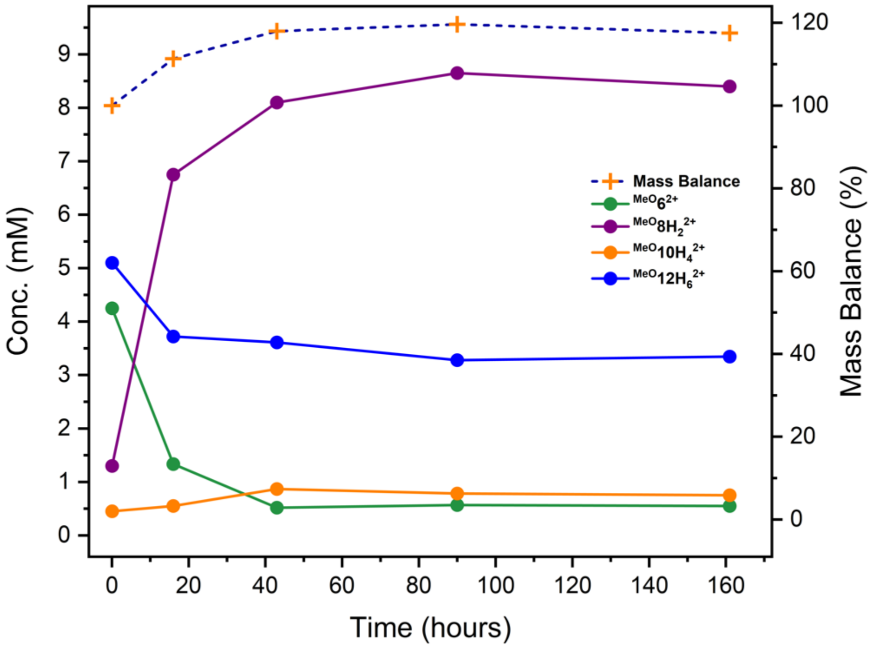

Figure S46. Mass balance analysis of the comproportionation reaction between $^{MeO}\mathbf{6}^{2+}$ (~5 mM) and $^{MeO}\mathbf{12H_6}^{2+}$ (~ 5mM). Final concentrations: $^{MeO}\mathbf{6}^{2+}$**:** 0.55 mM, $^{MeO}\mathbf{8H_2}^{2+}$: 8.5 mM, $^{MeO}\mathbf{10H_4}^{2+}$: 0.75 mM, $^{MeO}\mathbf{12_6}^{2+}$: 3.3 mM.

Comproportionation reactions: $^{H}6^{2+}$+ $^{MeO}12H_6{}^{2+}$; $^{MeO}6^{2+}$ + $^{H}12H_6{}^{2+}$; $^{H}6^{2+}$ + $^{H}12H_6{}^{2+}$: and $^{MeO}6^{2+}$ + $^{MeO}12H_6{}^{2+}$:

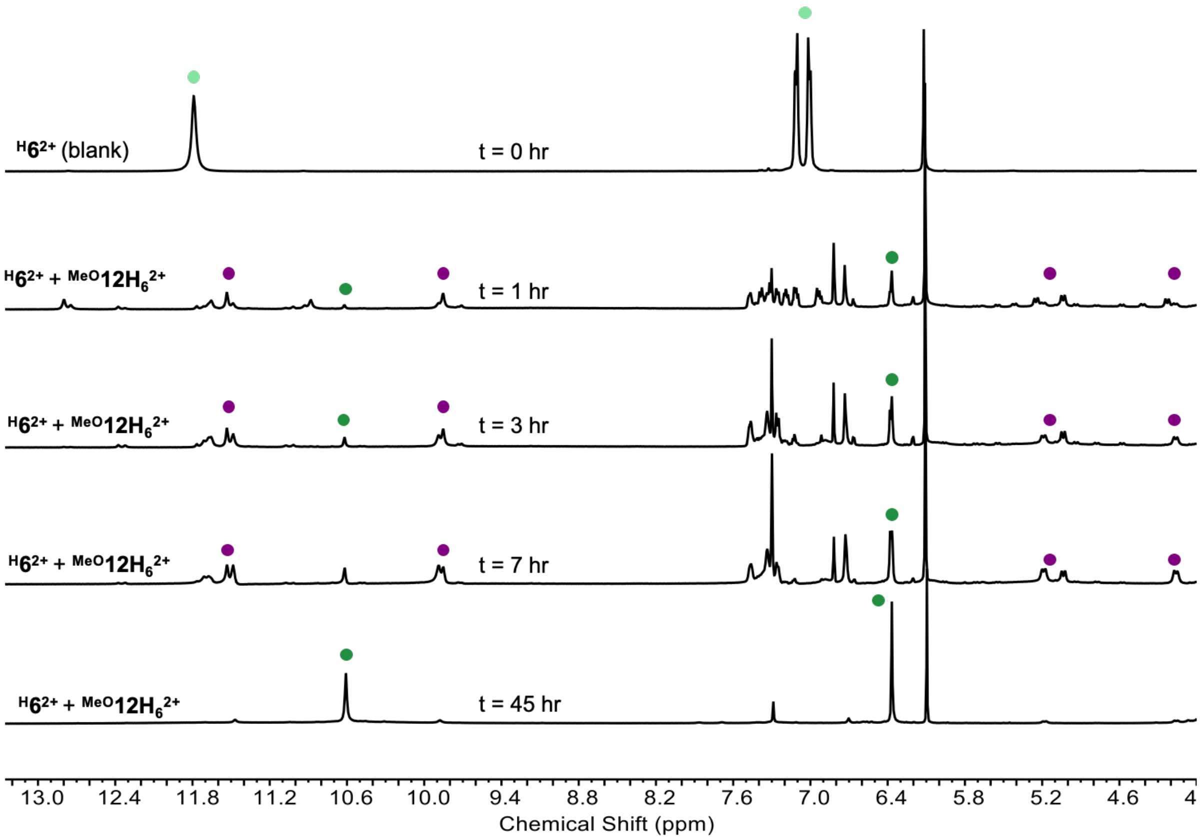

Figure S47. ^{1}H-NMR spectra in CD_3CN for the reaction: **$^{H}6^{2+}$** + **$^{MeO}12H_6{}^{2+}$.** The reaction produced **$^{H}8H_2{}^{2+}$** (purple dots) and a mixture of $^{X}8H_2{}^{2+}$-like species (see additional peaks around the peaks corresponding to **$^{H}8H_2{}^{2+}$**) until the full formation of **$^{MeO}6^{2+}$** (see green dots at time: 45 hours).

Scheme S2. Thermochemistry of the reaction between $^{H}\mathbf{6}^{2+}$ and $^{MeO}\mathbf{12H_6}^{2+}$; reaction between $^{H}\mathbf{6}^{2+}$ and $^{H}\mathbf{12H_6}^{2+}$; reaction between $^{MeO}\mathbf{6}^{2+}$ and $^{MeO}\mathbf{12H_6}^{2+}$; and reaction between $^{MeO}\mathbf{6}^{2+}$ and $^{MeO}\mathbf{12H_6}^{2+}$.

$$^{H}6^{2+} + {}^{MeO}12H_6^{2+} \longrightarrow {}^{H}8H_2^{2+} + {}^{MeO}10H_4^{2+}$$

$\Delta G^0_{react} = 2\,(\mathrm{BDFE}_{avg5,6}{}^{MeO} - \mathrm{BDFE}_{avg1,2}{}^{H})$

$\Delta G^0_{react} = 2\,(62.4 - 66.2) = -7.6$ kcal/mol

$$^{H}6^{2+} + {}^{H}12H_6^{2+} \longrightarrow {}^{H}8H_2^{2+} + {}^{H}10H_4^{2+}$$

$\Delta G^0_{react} = 2\,(\mathrm{BDFE}_{avg5,6}{}^{H} - \mathrm{BDFE}_{avg1,2}{}^{H})$

$\Delta G^0_{react} = 2\,(64.9 - 66.2) = -2.6$ kcal/mol

$$^{MeO}6^{2+} + {}^{MeO}12H_6^{2+} \longrightarrow {}^{MeO}8H_2^{2+} + {}^{MeO}10H_4^{2+}$$

$\Delta G^0_{react} = 2\,(\mathrm{BDFE}_{avg5,6}{}^{MeO} - \mathrm{BDFE}_{avg1,2}{}^{MeO})$

$\Delta G^0_{react} = 2\,(62.4 - 62.8) = -0.8$ kcal/mol

$$^{MeO}6^{2+} + {}^{H}12H_6^{2+} \longrightarrow {}^{MeO}8H_2^{2+} + {}^{H}10H_4^{2+}$$

$\Delta G^0_{react} = 2\,(\mathrm{BDFE}_{avg5,6}{}^{H} - \mathrm{BDFE}_{avg1,2}{}^{MeO})$

$\Delta G^0_{react} = 2\,(62.8 - 64.9) = 4.2$ kcal/mol

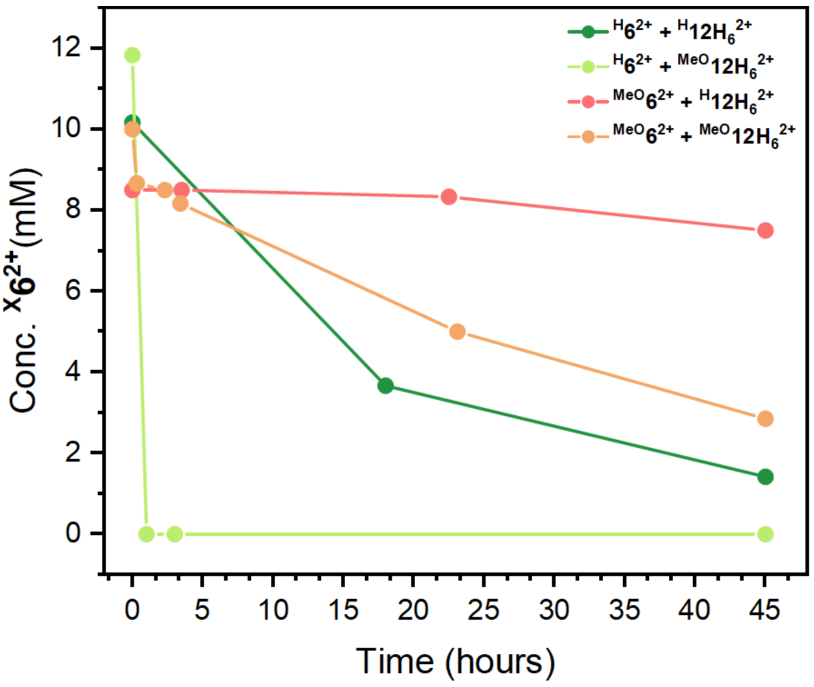

Figure S48. Kinetic traces for the reaction between $^{H}\mathbf{6}^{2+}$ and $^{MeO}\mathbf{12H_6}^{2+}$; reaction between $^{H}\mathbf{6}^{2+}$ and $^{H}\mathbf{12H_6}^{2+}$; reaction between $^{MeO}\mathbf{6}^{2+}$ and $^{MeO}\mathbf{12H_6}^{2+}$; and reaction between $^{MeO}\mathbf{6}^{2+}$ and $^{MeO}\mathbf{12H_6}^{2+}$.

6.3 Open-circuit potential (OCP) measurements

General procedure (adapted from protocol reported by Mayer and coworkers[4]): In the glovebox, 3 mL of CH_3CN solution containing 100 mM $[Bu_4N]PF_6$, 50 mM $Pyr.H^+$/Pyr. buffer was prepared, after which desired amount of substrates (0.75 µm of ^{X}**6**$^{2+}$ and 1.5 µm of ^{X}**8H**$_2^{2+}$, X = H, Me, and MeO) was added. For each substrate, open circuit potential measurements were collected at several ratios of the oxidized: reduced form (^{X}**6**$^{2+}$: ^{X}**8H**$_2^{2+}$), ranging between 0.5:1 and 2:1. Under Ar flow, OCP was recorded every second for 5–10 min, or until the potential has stabilized such that it changed less than 1.5 mV over 5 min. After each OCP measurement, Fc (in CH_3CN) was added as reference before a cyclic voltammetry (CV) measurement was carried out.

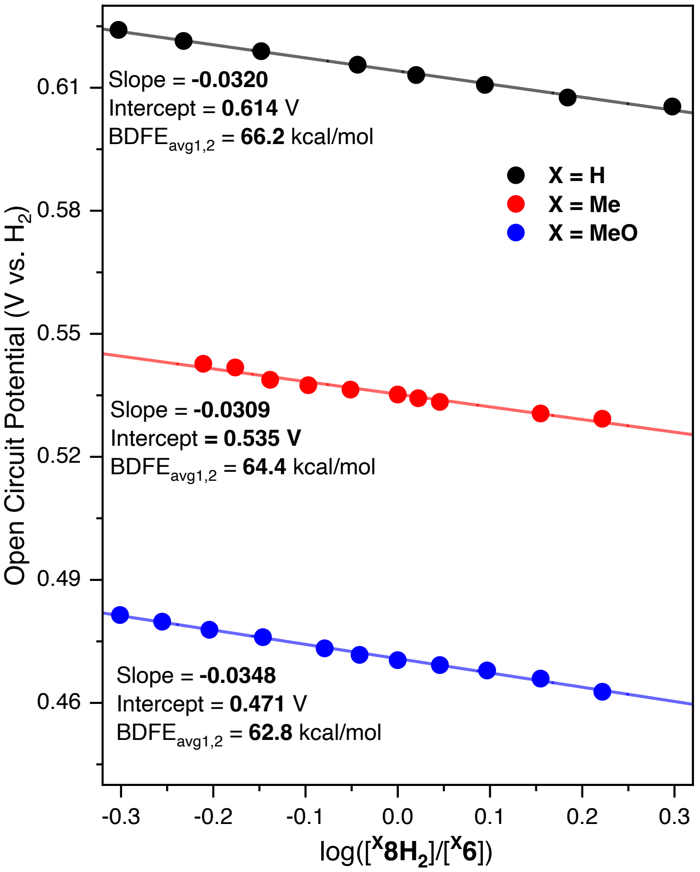

Figure S49. Overlaid open-circuit potential at different ratios of ^{H}**6**$^{2+}$/^{H}**8H**$_2^{2+}$ (black), ^{H}**6**$^{2+}$/^{H}**8H**$_2^{2+}$ (red), and ^{H}**6**$^{2+}$/^{H}**8H**$_2^{2+}$ (blue), plotted against the log ratios of the substrates. Colored scatter: original data, colored line: fitted curve.

The average BDFE for each $2H^+/2e^-$ transfer from ^{X}**6**$^{2+}$ to ^{X}**8H**$_2^{2+}$ can be directly calculated from the intercepts of each fitting curves:

$$BDFE_{avg}(^{H}\mathbf{6}^{2+}/^{H}\mathbf{8H}_2^{2+}) = 23.06 * 0.614 + 52.0$$

$$= 66.2 \text{ kcal/mol}$$

$$\text{BDFE}_{\text{avg}}(^{\mathbf{Me}}\mathbf{6}^{2+}/^{\mathbf{Me}}\mathbf{8H_2}^{2+}) = 23.06 * 0.535 + 52.0$$

$$= 64.4 \text{ kcal/mol}$$

$$\text{BDFE}_{\text{avg}}(^{\mathbf{MeO}}\mathbf{6}^{2+}/^{\mathbf{MeO}}\mathbf{8H_2}^{2+}) = 23.06 * 0.471 + 52.0$$

$$= 62.8 \text{ kcal/mol}$$

OCP measurements of other multiple H^+/e^- transfer involving $^{x}\mathbf{10H_4}^{2+}$ were not performed due to the instability of $^{x}\mathbf{10H_4}^{2+}$. OCP measurements of $^{x}\mathbf{6}^{2+}/^{x}\mathbf{12H_2}^{2+}$ were not successful because of the comproportionation reaction between $^{x}\mathbf{6}^{2+}/^{x}\mathbf{12H_2}^{2+}$.

7. Decoupled oxidation of diphenylhydrazine.

General procedure: In the glovebox, a 1 mL CD_3CN-d_3 solution containing 10 mM 1,3,5-trimethoxybenzene and ~ 10 mM $^{MeO}\mathbf{6}^{2+}$ was prepared. After the first ^{1}H-NMR spectrum (blank) was recorded, ~ 30 mM DPH was added to the NMR tube and the reaction was monitored over time. After full consumption of $^{MeO}\mathbf{6}^{2+}$, O_2 was bubbled into the solution for 30 seconds before each subsequent NMR measurement. The complete consumption of $^{MeO}\mathbf{12H_6}^{2+}$ and the consequent formation of $^{MeO}\mathbf{10H_2}^{2+}$, $^{MeO}\mathbf{8H_4}^{2+}$ and finally $^{MeO}\mathbf{6}^{2+}$ mark the completion of the first cycle. After the first cycle, the solution was degassed and purged with N_2, transferred into the glovebox and reacted with another 3 equiv of DPH calculated based on recovered ECPB. This strategy was repeated for one more cycle and the results are shown below.

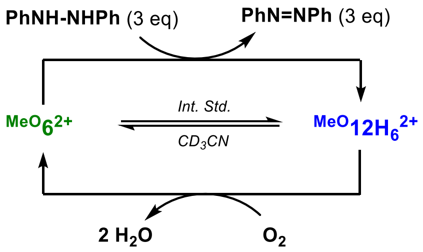

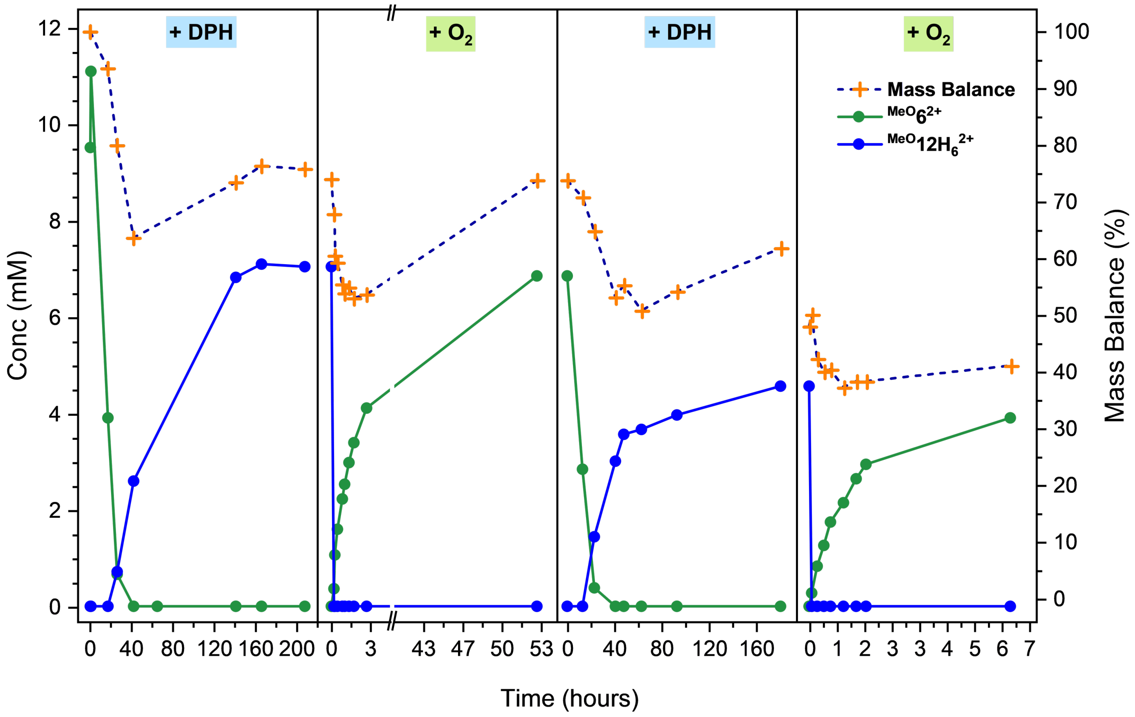

Figure S50. Mass balance analysis of the decoupled oxidation of PhNHNHPh using O_2 as oxidant and $^{MeO}\mathbf{6}^{2+}$ as ECPB.

Table S11. Summary of concentrations (in mM) of ECPBs at the end of each half-cycle of the 6H$^+$/6e$^-$ decoupled oxidation of DPH. The "Reduction/oxidation" in the table refers to the reduction/oxidation of the ECPB.

**Cycle**	[^{MeO}**6**$^{2+}$]	[^{MeO}**8H$_2$**$^{2+}$]	[^{MeO}**10H$_4$**$^{2+}$]	[^{MeO}**12H$_6$**$^{2+}$]	**Mass Balance** (%)
Start	9.53	0.00	0.00	0.00	100.0
Reduction1	0.00	0.00	0.16	7.22	75.8
Oxidation 1	6.87	0.18	0.00	0.00	73.8
Reduction2	0.00	1.25	0.06	4.58	61.8
Oxidation2	3.92	0.00	0.00	0.00	41.1

8. Buffering experiments

General procedure: In the glovebox, a 1 mL CD_3CN solution containing 10 mM 1,3,5-trimethoxybenzene and equimolar amounts of $^{\mathbf{X}}\mathbf{6}^{2+}$ (~10 mM) and $^{\mathbf{X}}\mathbf{12H_6}^{2+}$ (~10 mM) was prepared and transferred into an NMR tube. ^{1}H-NMR spectra (blank) were recorded over time until no changes in concentration were observed, i.e. until the ECPB system equilibrated. After the solution equilibrated, ~5 mM PCET regent (either 1,4-Benzoquinone (BQ) or 4,5-MeO-1,4-hydroquinone ($(MeO)_2$-H_2Q)) was added to the NMR tube and the reaction was again monitored over time to observe the shifts in equilibria. The experiments were performed at room temperature.

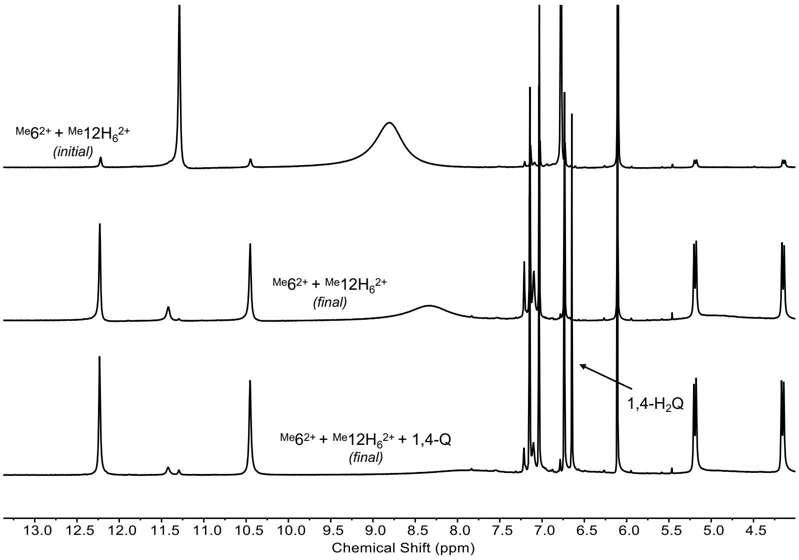

Figure S51. ^{1}H-NMR spectra in CD_3CN for the reaction buffering reaction $^{\mathbf{Me}}\mathbf{6}^{2+}$/ $^{\mathbf{Me}}\mathbf{12H_6}^{2+}$ + 1,4-BQ.

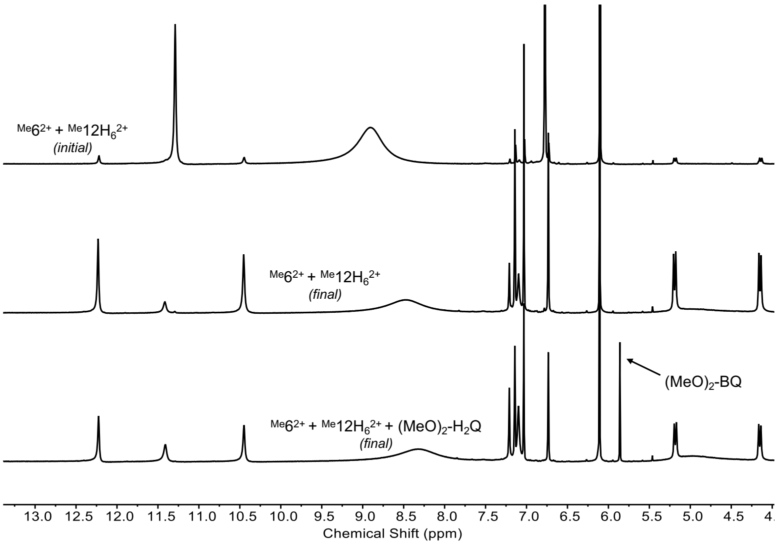

Figure S52. ^{1}H-NMR spectra in CD_3CN for the reaction buffering reaction $^{\mathbf{Me}}\mathbf{6}^{2+}$/ $^{\mathbf{Me}}\mathbf{12H_6}^{2+}$ + 4,5-MeO-1,4-hydroquinone.

9. Kinetics

General procedure: A 1 mL CD_3CN solution containing 10 mM 1,3,5-trimethoxybenzene and ~ 2 mM $^{Me,H}\mathbf{5}^{+}$ (Cu) / $^{Me}\mathbf{6}^{2+}$ (Fe) was prepared inside a glovebox. After the first ^{1}H-NMR spectrum (blank) was recorded, 20 mM DPH was added to each sample in order to study pseudo first order kinetics. The reaction (consumption of the complex and formation of DPD) was monitored over time. The experiments were performed at room temperature.

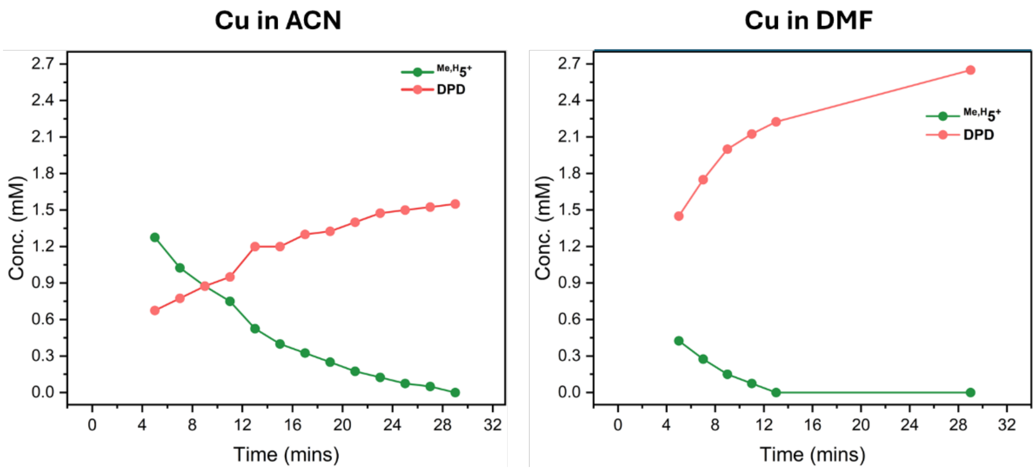

Figure S53. Kinetic profiles for the reaction between $^{Me,H}\mathbf{5}^{+}$ and PhNHNHPh (DPH) followed by NMR in CD_3CN and DMF-d_7. Note: these reactions produced $^{Me,H}\mathbf{1H_4}^{+}$ and PhN=NPh (DPD).

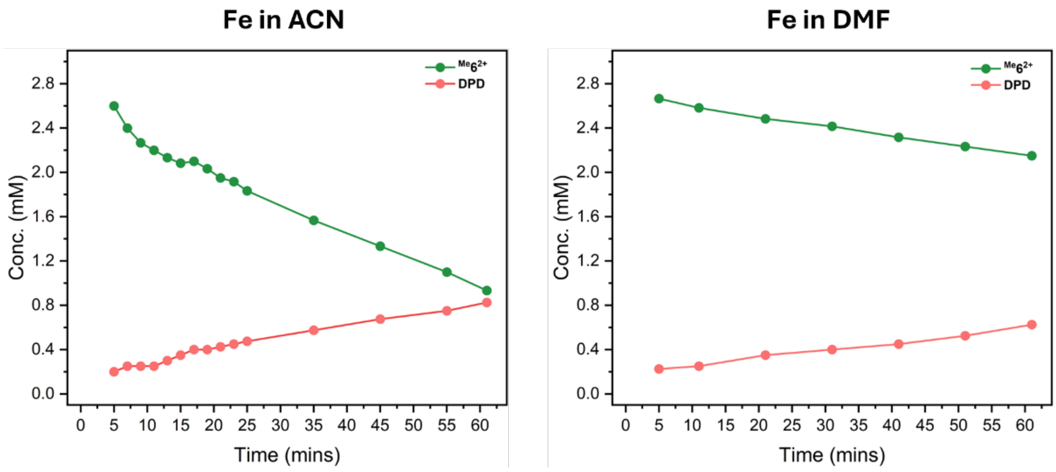

Figure S54. Kinetic profiles for the reaction between $^{Me}\mathbf{6}^{2+}$ and PhNHNHPh (DPH) followed by NMR in CD_3CN and DMF-d_7. Note: these reactions produced $^{Me}\mathbf{8H_2}^{2+}$ and PhN=NPh (DPD).

10. References

(1) Wu, T.; Puri, A.; Qiu, Y. L.; Ye, D.; Sarma, R.; Wang, Y.; Kowalewski, T.; Siegler, M. A.; Swart, M.; Garcia-Bosch, I. Tuning the Thermochemistry and Reactivity of a Series of Cu-Based 4H+/4e– Electron-Coupled-Proton Buffers. *Inorg. Chem.* **2024**, *63* (20), 9014-9025.
(2) Matsumoto, T.; Chang, H.-C.; Wakizaka, M.; Ueno, S.; Kobayashi, A.; Nakayama, A.; Taketsugu, T.; Kato, M. Nonprecious-Metal-Assisted Photochemical Hydrogen Production from ortho-Phenylenediamine. *J. Am. Chem. Soc.* **2013**, *135* (23), 8646-8654.
(3) Matsumoto, T.; Yamamoto, R.; Wakizaka, M.; Nakada, A.; Chang, H.-C. Molecular Insights into the Ligand-Based Six-Proton- and Six-Electron-Transfer Processes Between Tris-ortho-Phenylenediamines and Tris-ortho-Benzoquinodiimines. *Chem.-Eur. J.* **2020**, *26* (43), 9609-9619.
(4) Wise, C. F.; Agarwal, R. G.; Mayer, J. M. Determining Proton-Coupled Standard Potentials and X–H Bond Dissociation Free Energies in Nonaqueous Solvents Using Open-Circuit Potential Measurements. *J. Am. Chem. Soc.* **2020**, *142* (24), 10681-10691.
